# Synthesis of Quinazoline Derivatives and Mechanistic Approaches in Lung and Breast Cancers

**DOI:** 10.3390/molecules31183163

**Published:** 2026-09-08

**Authors:** Aybüke Züleyha Kaya, Beyzanur Tutuş, Şevval Karaca Arpa, Asaf Evrim Evren, Gülşen Akalin Çiftçi, Halide Edip Temel, Leyla Yurttaş

**Affiliations:** 1 Department of Pharmaceutical Chemistry, Faculty of Pharmacy, Anadolu University, Eskişehir 26470, Türkiye; 2 Department of Pharmaceutical Chemistry, Graduate School, Anadolu University, Eskişehir 26470, Türkiye; 3 Kırıkhan Vocational School, Hatay Mustafa Kemal University, Hatay 31440, Türkiye; 4 Department of Biochemistry, Graduate School, Anadolu University, Eskişehir 26470, Türkiye; 5 Department of Biochemistry, Faculty of Pharmacy, Anadolu University, Eskişehir 26470, Türkiye

**Keywords:** quinazolinone, cytotoxicity, apoptosis, caspase-3 activation, cell cycle analysis, EGFR inhibition

## Abstract

The quinazoline/quinazolinone ring is known as a unique scaffold, and its derivatives possess a broad biological activity profile, including antibacterial, antifungal, anticonvulsant, anti-inflammatory, anti-HIV, and analgesic activity, primarily focusing on anticancer activity. In this study, the synthesis of 2-[[4-oxo-3-(substituted phenyl)-3,4-dihydro-(substituted quinazolin-2-yl)]thio]-*N*′-(aryl/heteroaryl methylene)acetohydrazide (**4a**–**4x**) derivatives and their potential anticancer activities were investigated on the lung cancer A549 cell line, the breast cancer MCF-7 cell line, and healthy fibroblast L929 cell line. Compounds **4c**, **4i**, **4m**, and **4u** were identified as the most cytotoxic and selective molecules on the A549 cell line (IC_50_: 44.75–78.13 µM), while **4i**, **4l**, **4m**, and **4u** were identified as the most cytotoxic and selective molecules on the MCF-7 cell line (IC_50_: 14.43–29.39 µM). The mechanisms of action of their anticancer activities were examined and studied. It was determined that these compounds induced strong apoptosis and significantly activated caspase-3 activation in both cell types and that they interrupted the cell cycle in the pre-G (sub G0) phase. Compounds **4m** and **4u** exhibited EGFR inhibition (IC_50_: 4.80 µM, IC_50_: 5.40 µM, respectively) at a level similar to the standard drug gefitinib (IC_50_: 1.86 ± 0.43 µM). Based on the results of the biological activity assays, molecular docking, and molecular dynamics simulation studies, the 4-quinazolinone–acetyl hydrazone scaffold can be considered a promising structural framework with potential anticancer activity. More specifically, the findings of this study indicate that the acetyl moiety may function as an important pharmacophoric group, while the trisubstituted quinazolinone core may represent a favorable structural feature for caspase-3 activation. However, the same structural framework appears to be less favorable for EGFR inhibition, possibly due to steric constraints within the EGFR binding pockets.

## 1. Introduction

**Figure 1:**
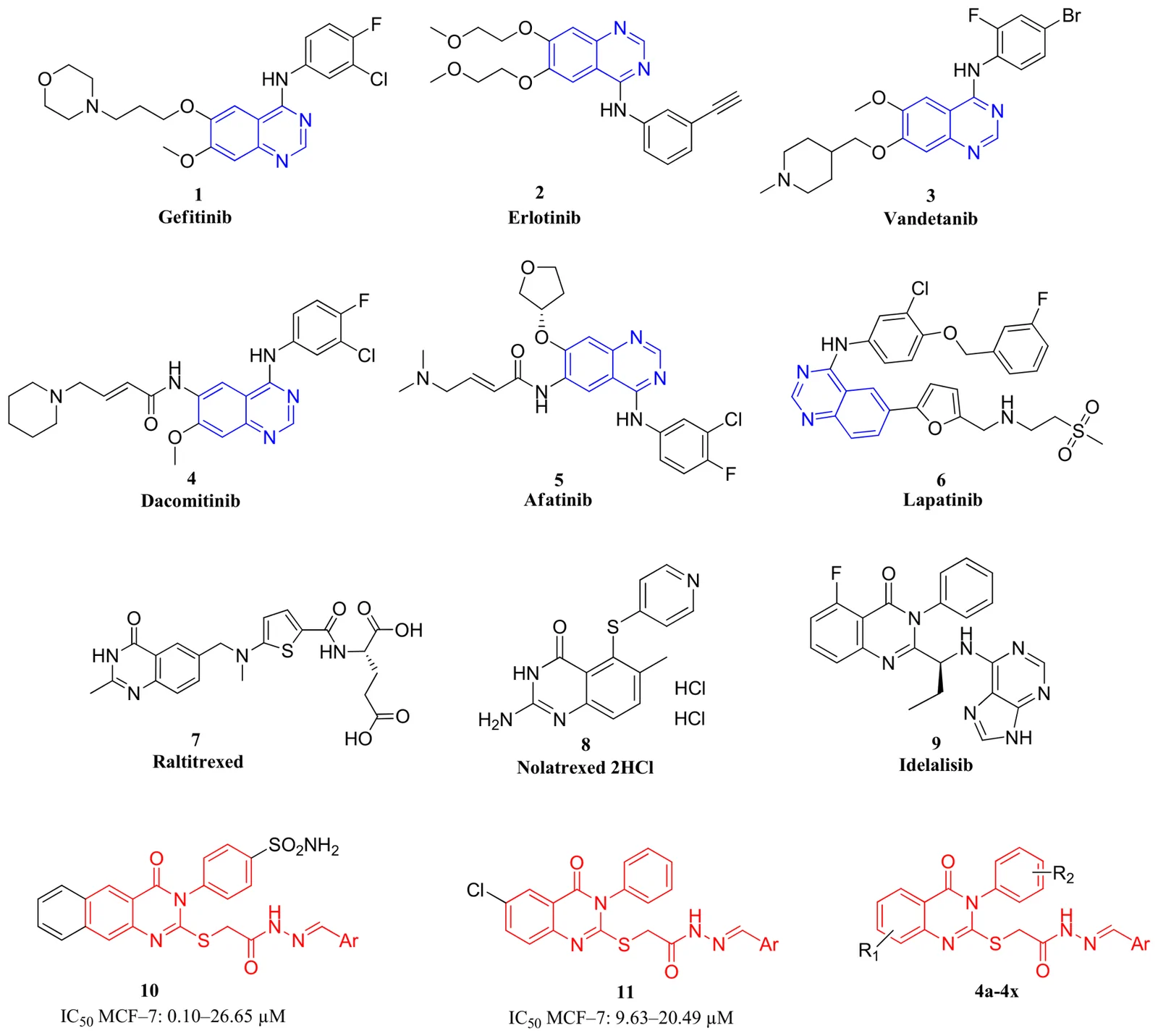
Quinazoline/quinazolinone-based anticancer drugs, some reported molecules (**10**, **11**) and the synthesized compounds in this study (**4a**–**4x**).

Nitrogen-containing heterocyclics are a valuable source of pharmacophores for the development of novel active molecules, and more than 75% of molecules approved by the US Food and Drug Administration (FDA) contain *N*-heterocyclic moieties in their structures. Molecules bearing nitrogen-containing heterocyclics are valuable scaffolds for the development of novel inhibitors of various cellular signaling biomolecules [1]. Quinoline/quinazolinone derivatives possess many biological properties, such as antimicrobial [2], antimalarial [3], anti-inflammatory [4], anticonvulsant [5], antihypertensive [6], antidiabetic [7], antioxidant [8], antitubercular [9], antiviral [10], CFTR inhibitory [11], anti-HIV [12], and anticancer [13,14]. They have come to the forefront in the discovery of new anticancer drugs, especially in drug design studies, as they are a very promising core in the research of various malignancies in cancer treatments. Among many heterocyclic scaffolds, quinazoline and quinazolinone derivatives are frequently reported with an anticancer effect profile. The anticancer activity of quinazoline-based cytotoxic agents is mostly associated with changes in cell cycle progression [15], induction of apoptosis [16], and modification of autophagy [17]. Molecules with anticancer activity, mostly of the 4-anilinoquinazoline type, inhibit tumor growth by inhibiting DNA replication and transcription through protein kinase inhibition [18]. In addition, some of these anticancer derivatives overcome breast cancer resistance by inhibiting breast cancer resistance proteins [19]. The US Food and Drug Administration (FDA) has approved more than 20 drugs containing a quinazoline or quinazolinone core as anticancer agents in the last two decades. One of the most important examples is Dacomitinib, newly approved in 2018 for the treatment of non-small cell lung carcinoma [20]. These drugs exert their effects through different mechanisms of action to prevent the spread of cancer cell progression, including inhibition of different kinases [21], tubulin [22], kinesin spindle protein [23], and similar proteins [24]. Among the quinazoline-derived anticancer drugs in clinical use approved by the FDA are gefitinib, erlotinib, lapatinib, afatinib, dacomitinib and vandetanib (Figure 1(**1**–**6**)). Gefitinib (Iressa^®^) was approved by the FDA in 2003 for the treatment of locally advanced or metastatic non-small cell lung cancer (NSCLC) after failure of both platinum-based and docetaxel chemotherapy. In 2004, erlotinib (Tarceva^®^) was approved by the FDA for the treatment of NSCLC. Furthermore, in 2005, the FDA approved erlotinib in combination with gemcitabine for the treatment of locally advanced, metastatic pancreatic cancer.

The pathways involved in the anticancer activity of quinazolinones include tyrosine kinase inhibition, apoptosis induction, and caspase activation [25,26], as well as mitochondrial membrane depolarization [27] and MMP-9 inhibition [28]. There are also other enzymes inhibited by anticancer quinazolinones, such as thymidylate synthase [29], poly ADP-ribose polymerase-1 (PARP) [30], and topoisomerase [31]. Therefore, quinazolinones exert their chemotherapeutic activity through various molecular interactions and mechanisms of action [24]. Due to the intense interest in quinazoline derivatives, studies on potential anticancer precursor molecules are ongoing [32]. High cytotoxic effects have been detected in compounds synthesized from 2-thioxoquinazoline-4-one derivatives [33]. A series of substances containing quinazolinone and hydrazine/hydrazone have also been determined to exhibit antiproliferative effects and induce apoptosis [34]. Idelalisib, with a quinazolinone-4-one structure, is a highly effective drug and a selective PI3Kδ inhibitor (IC_50_ = 2.5 nM). It is an FDA-approved antineoplastic drug for the treatment of hematological malignancies and is used as a second-line treatment option for patients with relapsed chronic lymphocytic leukemia [35]. Another drug, raltitrexed, with a quinazolinone-4-one structure, exhibits anticancer effects through the inhibition of thymidylate synthesis. Nolatrexed HCl similarly exhibits anticancer effects by inhibiting thymidylate synthesis (Figure 1(**7**–**9**)) [36]. In 2017, the anticancer efficacy of a group of quinazolinone-chalcone derivatives synthesized was investigated on several cancer cells, including A549 and MCF-7. Compounds exhibiting high cytotoxicity have been determined to cause apoptosis and loss of membrane polarization [37]. Low-inhibition concentrations against MCF-7 cancer cells have been reported in compounds carrying the 2-[(substituted-4-oxo-3-phenyl-3,4-dihydroquinazolin-2-yl)thio]-*N*′-(2-substituted benzylidene)-acetohydrazide backbone (Figure 1(**10**–**11**)) [33,38]. In addition, many researchers have reported anticancer effects of quinazolinones with various variations containing sulfur bridges at the second position and aryl groups at the third position [33,39,40,41,42,43].

Based on this literature review and our previous studies [44,45,46], novel quinazolinone derivatives were synthesized in this study, and their effects on lung and breast cancer were investigated in terms of their mechanism of action.

## 2. Results

### 2.1. Chemistry

**Scheme 1:**
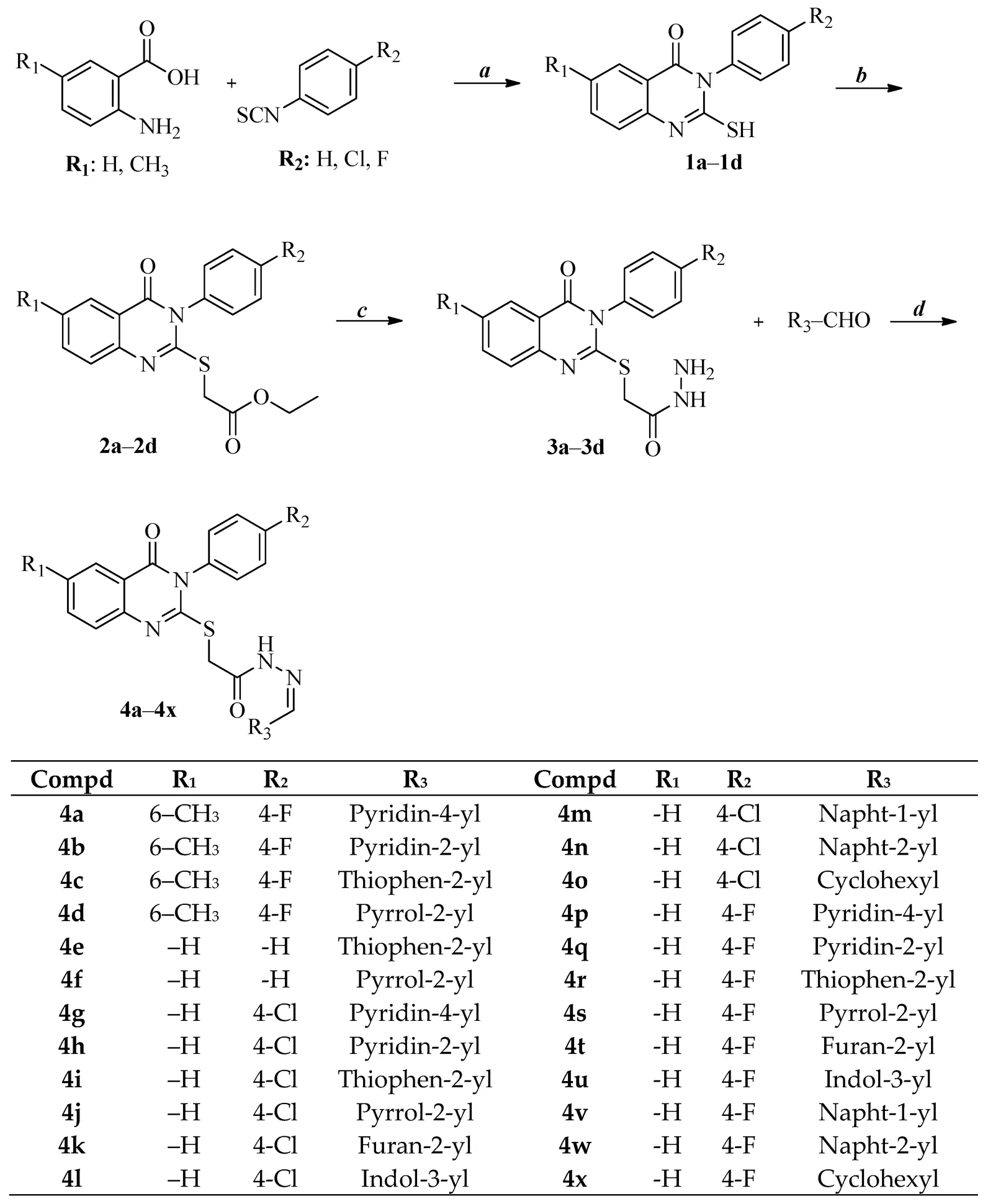
Synthesis pathway of target molecules (**4a**–**4x**). Reaction conditions: (**a**) absolute EtOH, TEA, reflux, 2 h; (**b**) ethyl iodoacetate, acetone, K_2_CO_3_, reflux, 6 h; (**c**) hydrazine hydrate, EtOH, reflux, overnight; (**d**) EtOH, reflux, 1 h.

This study aimed to obtain 2-[[4-oxo-3-(substituted phenyl)-3,4-dihydro-(substituted quinazolin-2-yl)]thio]-*N*′-(aryl/heteroaryl methylene)acetohydrazide (**4a**–**4x**) derivatives. The intermediates 2-mercapto-3-(substituted phenyl)-3*H*-substituted quinazolin-4-one (**1a**–**1d**) were obtained by dissolving suitable anthranilic acid derivatives, suitable isothiocyanate derivatives, and triethylamine (TEA) in absolute ethanol. The obtained intermediates (**1a**–**1d**) and ethyl iodoacetate were refluxed in acetone with a catalytic amount of K_2_CO_3_ to obtain derivatives of [4-oxo-3-(substituted phenyl)-3,4-dihydro-(substituted quinazolin-2-yl)thio]-acetic acid ethyl ester (**2a**–**2d**). Reaction of **2a**–**2d** with hydrazine hydrate in ethanol resulted in the formation of 2-[[4-oxo-3-(substituted phenyl)-3,4-dihydro-(substituted quinazolin-2-yl)]thio]acetohydrazide (**3a**–**3d**) derivatives. In the final step of the synthesis, the hydrazide intermediates (**3a**–**3d**) were treated with various aldehydes under reflux in ethanol to obtain the final products (**4a**–**4x**) as shown Scheme 1. The structure of synthesized compounds was confirmed by IR, HRMS, ^1^H- and ^13^NMR spectroscopic analysis. The IR spectra of compounds **4n**, **4o**, and **4x** exhibited characteristic stretching bands corresponding to C=N and C=O functionalities, along with deformation bands attributable to the 1,4-disubstituted benzene ring. In addition, characteristic aromatic and aliphatic C–H stretching bands were observed. HRMS analysis of **4a**–**4x** showed that the observed molecular weights were consistent with the calculated values. The final compounds exhibit E/Z isomerism. Analysis of the NMR spectra revealed duplicate singlet signals, indicating the presence of E and Z isomers in solution. S-CH_2_ bond is common in all final compounds and observed in the 3.98–4.61 ppm range in the ^1^H-NMR spectra. Also, **4a**–**4d** contains methyl groups, and it is clearly detected at both proton and carbon spectra at 2.39–2.47 ppm and 21.8–21.9, respectively. The amino group of hydrazone moiety was detected at 11.32–12.07 ppm as two singlets and singlets. The carbonyl group was attached to the hydrazone group, observed between 168.2 and 169.7 ppm in the carbon NMR spectra. The aromatic hydrogen area of proton NMR spectra mostly overlapped and was observed as multiplet peaks. Spectroscopic analyses confirmed the successful synthesis of the target compounds (Figures S1–S72).

### 2.2. Biochemistry

#### 2.2.1. Cytotoxicity

Twenty-four synthesized (**4a**–**4x**) and structurally validated compounds were tested on A549 lung cancer, MCF-7 breast cancer, and L929 non-cancerous control cells; cisplatin and gefitinib were used as positive controls, and the results are presented in micromolar units in Table 1. The IC_50_ values of the compounds were determined to be between 14.43 µM and >500 µM. The most active compounds against the A549 cell line were identified as compound **4m** with 44.75 µM, compound **4u** with 58.69 µM, compound **4i** with 62.13 µM, and compound **4c** with 78.13 µM. These four compounds were evaluated selectively because they showed high-dose cytotoxicity against the L929 cell line. The IC_50_ value of standard drugs like cisplatin was found to be 14.00 µM against the A549 cell line, while gefitinib’s IC_50_ value was 35.58 µM. Although none of the compounds reached these values, **4m** compound (IC_50_: 44.75 µM) stands out as the most selective and effective compound on A549 cells because it did not exhibit cytotoxicity even at 500 µM against L929. Compound **4h** exhibited an inhibitory concentration of 89.62 µM but also showed cytotoxicity at a lower dose against the L929 cell line (IC_50_: 78.18 µM); therefore, this compound was considered toxic. Among the compounds, **4a**, **4e**, **4h**, **4l**, **4r**, and **4x** showed moderate to weak antiproliferative activity, with **4e**, **4h** and **4l** exhibiting a non-selective profile. All other compounds tested failed to demonstrate sufficient inhibition against the A549 cell line, even at the highest dose tested, 500 µM.

The IC_50_ values of the compounds on the MCF-7 breast cancer cell line were found to be variable, and it was determined that the molecules exhibited non-selective and higher potency compared to standard drugs cisplatin and gefitinib. The IC_50_ value of the standard drug cisplatin was found to be 139.55 µM and the IC_50_ value of gefitinib was found to be 465.32 µM, and the IC_50_ values of the four most active compounds were determined to be between 14.43 and 29.39 µM. According to these values, compound **4m,** the most active compound with an IC_50_ value of 14.43 µM, showed approximately ten-times higher activity than the standard drug cisplatin; compound **4u,** with an IC_50_ value of 26.15 µM, showed approximately six-times higher activity; compound **4i,** with an IC_50_ value of 27.59 µM, showed approximately five-times higher activity; and compound **4l,** with an IC_50_ value of 29.39 µM, showed approximately four-times higher activity. These four compounds were considered selective against the MCF-7 cell line because they exhibited higher toxicity to L929 at higher doses. Compounds **4b** and **4x** exhibited non-selective antiproliferative activity against the MCF-7 cell line, with IC_50_ values of 284.69 and 128.05 µM, respectively, similar to standard drugs. Compounds **4c**, **4e**, and **4h**, although showing cytotoxicity, exhibited this in a non-selective manner and were, therefore, considered inactive along with the remaining compounds.

#### 2.2.2. Apoptosis

**Figure 2:**
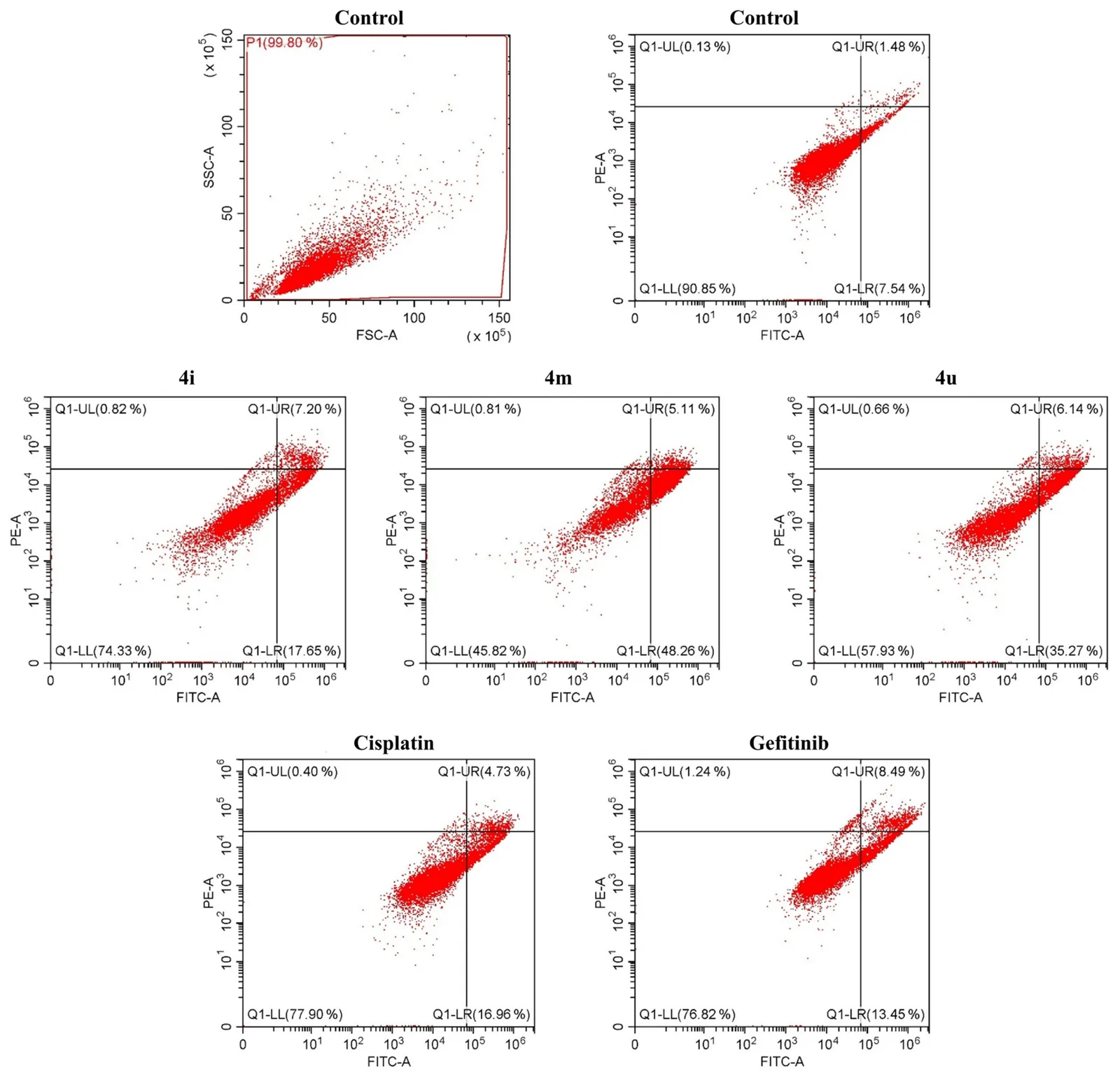
Flow cytometric analysis of compounds **4i**, **4m**, **4u**, cisplatin, and gefitinib at IC_50_ concentrations on A549 cells. Q1: necrotic cells, Q2: late apoptotic cells, Q3: live cells, Q4: early apoptotic cells.

Compounds **4i**, **4m**, and **4u**, which showed the highest cytotoxic activity against the A549 cell line, were used in apoptosis studies with the standard drugs cisplatin and gefitinib. The compounds were tested via flow cytometry according to the Annexin/FITC propidium iodide staining method, and the results are presented as percentages in Table 2 and Figure 2. Compounds **4i**, **4m**, **4u**, cisplatin, and gefitinib were tested using IC_50_ doses (62.13, 44.75, 58.69, 14.00, and 35.58 µM), with at least 10.000 A549 cells applied to quadrant analysis for each sample. Flow cytometry analysis of the compounds determined the percentages of early apoptotic, late apoptotic, viable, and necrotic cells. Accordingly, the percentage of living cells in control cells was 90.85%, which is lower in all test compounds. Apoptosis is programmed cell death, a death mechanism intended to lead cancer cells to death. Accordingly, the percentage of early apoptotic cells caused by test compounds (**4i**, **4m**, and **4u**) is significantly higher than the 16.96% induced by cisplatin and the 13.45% induced by gefitinib. The percentages of late apoptotic cells caused by test compounds were found to be higher when compared with the standard drug cisplatin (4.73%), and even lower when compared even with gefinib (8.49%). When the percentage of necrotic cells caused by the test compounds was examined, none of the test compounds induced as much necrotic cells as the standard drug gefitinib, which resulted in a necrotic cell percentage of 1.24%.When the total apoplectic population (early + late apoptosis) induced by the compounds and standard drugs was calculated, cisplatin and gefitinib induced similar levels of apoptosis, with percentages of 21.69% and 21.94%, respectively. Compound **4i** exceeded the total apoptosis percentage, of 24.85%, respectively, surpassing the standard drugs. Compounds **4m** (53.37%) and **4u** (41.41%), on the other hand, induced higher levels of total apoptosis, approximately doubling or exceeding the apoptosis rates produced by the positive controls, exhibiting an excellent rate of apoptosis. It is clearly seen that compounds **4m** and **4u** promoted A549 cell death of A549 cells by inducing apoptosis.

**Figure 3:**
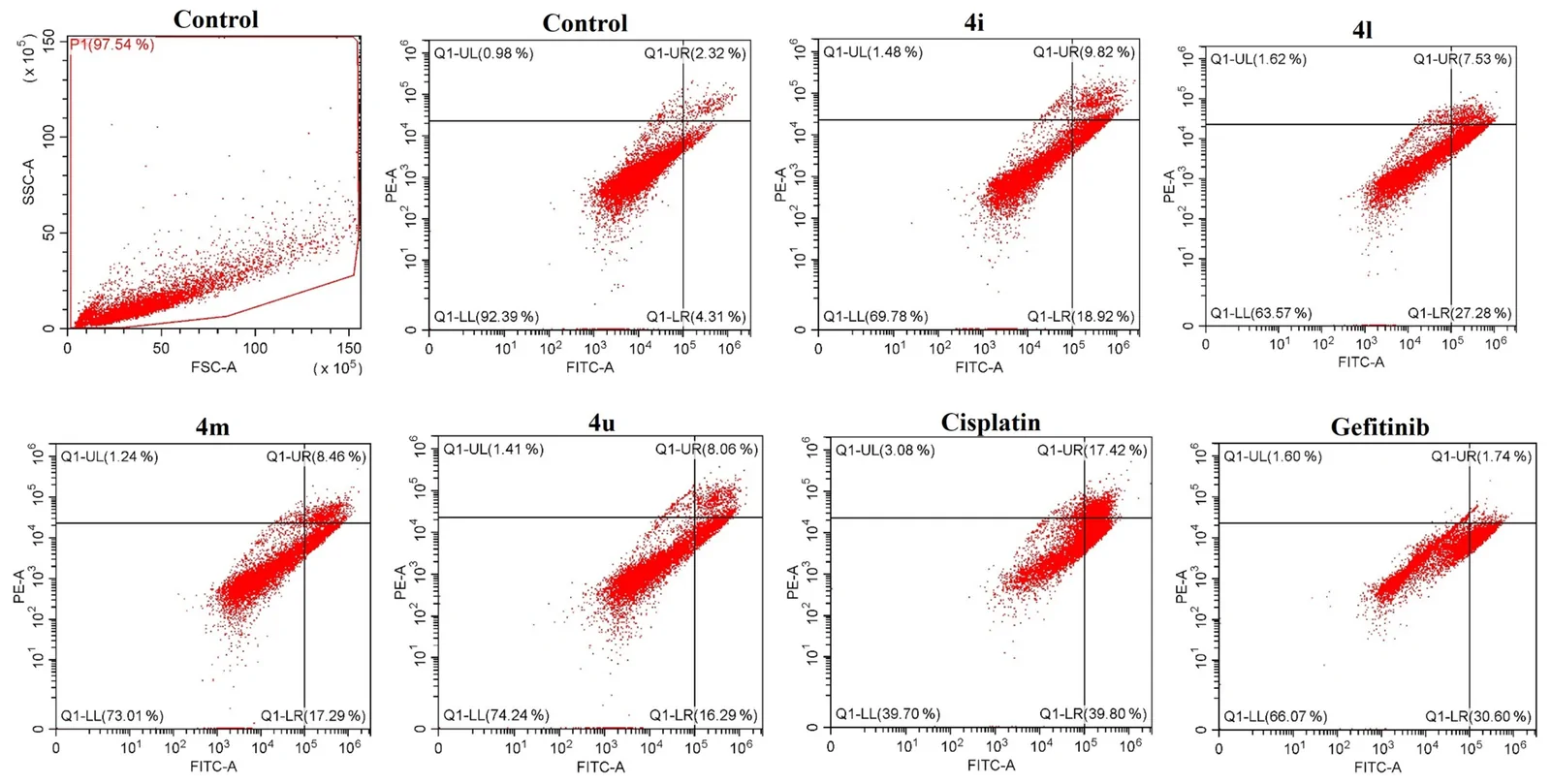
Flow cytometric analysis of compounds **4i**, **4l**, **4m**, **4u** cisplatin and gefitinib at IC_50_ concentrations on MCF-7 cells. Q1: necrotic cells, Q2: late apoptotic cells, Q3: live cells, Q4: early apoptotic cells.

Compounds **4i**, **4l**, **4m**, and **4u** were tested at IC_50_ doses of cisplatin and gefitinib (27.59, 29.39, 14.43, 26.15, 139.55, and 465.32 µM), with at least 10.000 MCF-7 cells per sample subjected to quadrant analysis. When the compounds were evaluated for their effect on apoptosis and the percentages of viable and necrotic cells in the MCF-7 cell line, none of the compounds induced total apoptosis cell percentages that were as high as those observed with cisplatin treatment (57.22%), as can be seen in Table 3 and Figure 3. Regarding necrotic cell percentages, all compounds induced less necrosis than cisplatin, and either less or equal to gefitinib. When compared to the total apoptosis percentage caused by gefitinib, only compound **4l**, with 34.81%, caused more apoptosis induction than the standard drug. Of the other compounds, compound **4i** exhibited apoptosis induction at 28.74%, **4m** at 25.75%, and **4u** at 24.35%. Although these values were lower than that observed for cisplatin, they were comparable to the apoptosis induction level observed for gefitinib. It was also determined that tested compounds exhibited a percentage at least four-times higher than the percentage of late apoptotic cells induced by gefitinib (1.74%). All of the compounds were found to induce apoptosis in MCF-7 cells; however, the extent of apoptosis induction was lower than that observed for cisplatin.

#### 2.2.3. Caspase-3 Activation Analysis

**Figure 4:**
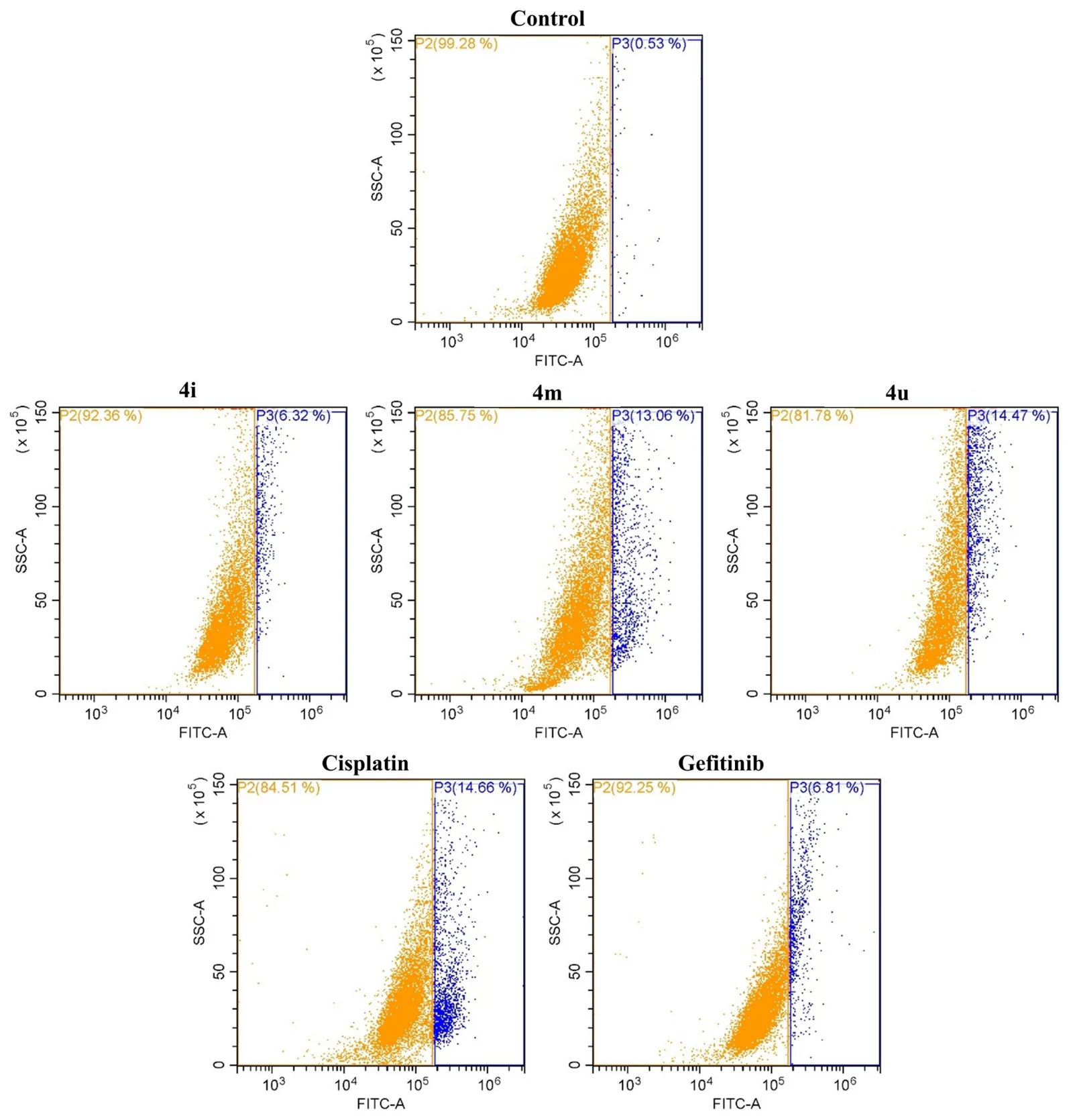
Flow cytometry quadrants analysis of caspase-3 activity in A549 cells treated with compounds **4i**, **4m**, **4u**, cisplatin, and gefitinib. P1: caspase-3-positive cells, P2: caspase-3-negative cells.

To investigate caspase-3 activation as an indicator of early-phase apoptosis, A549 cells were incubated with compounds **4i**, **4m**, **4u**, cisplatin, and gefitinib for 24 h. Cell percentages and quadrants determined by flow cytometry analysis are illustrated in Table 4 and Figure 4.

Flow cytometry analysis revealed that treatment with compounds **4i**, **4m**, and **4u** of cisplatin and gefitinib resulted in caspase-3-positive A549 cell percentages of 6.32%, 13.06%, 14.47%, 14.66%, and 6.81%, respectively, while the percentages of caspase-3-negative cells of these compounds were 92.36%, 85.75%, 81.78%, 84.51%, and 92.25%, respectively. Based on these results, the compounds induced caspase-3 activation in A549 cells compared to the control group (0.53%). Furthermore, compounds **4m** (13.06%) and **4u** (14.47%) induced caspase-3 activation at rates close to those caused by cisplatin (14.66%). While compound **4i** (6.32%) did not exhibit caspase-3 activation as high or close to cisplatin on A549 cells, it showed similar rates to gefitinib (6.81%), another standard compound. Therefore, all compounds exhibited caspase-3 activation comparable to standard drugs, and it was determined that they induce early-phase apoptosis early and via the caspase-3 pathway. This finding is supported by the high percentage of early apoptosis observed in apoptosis studies.

**Figure 5:**
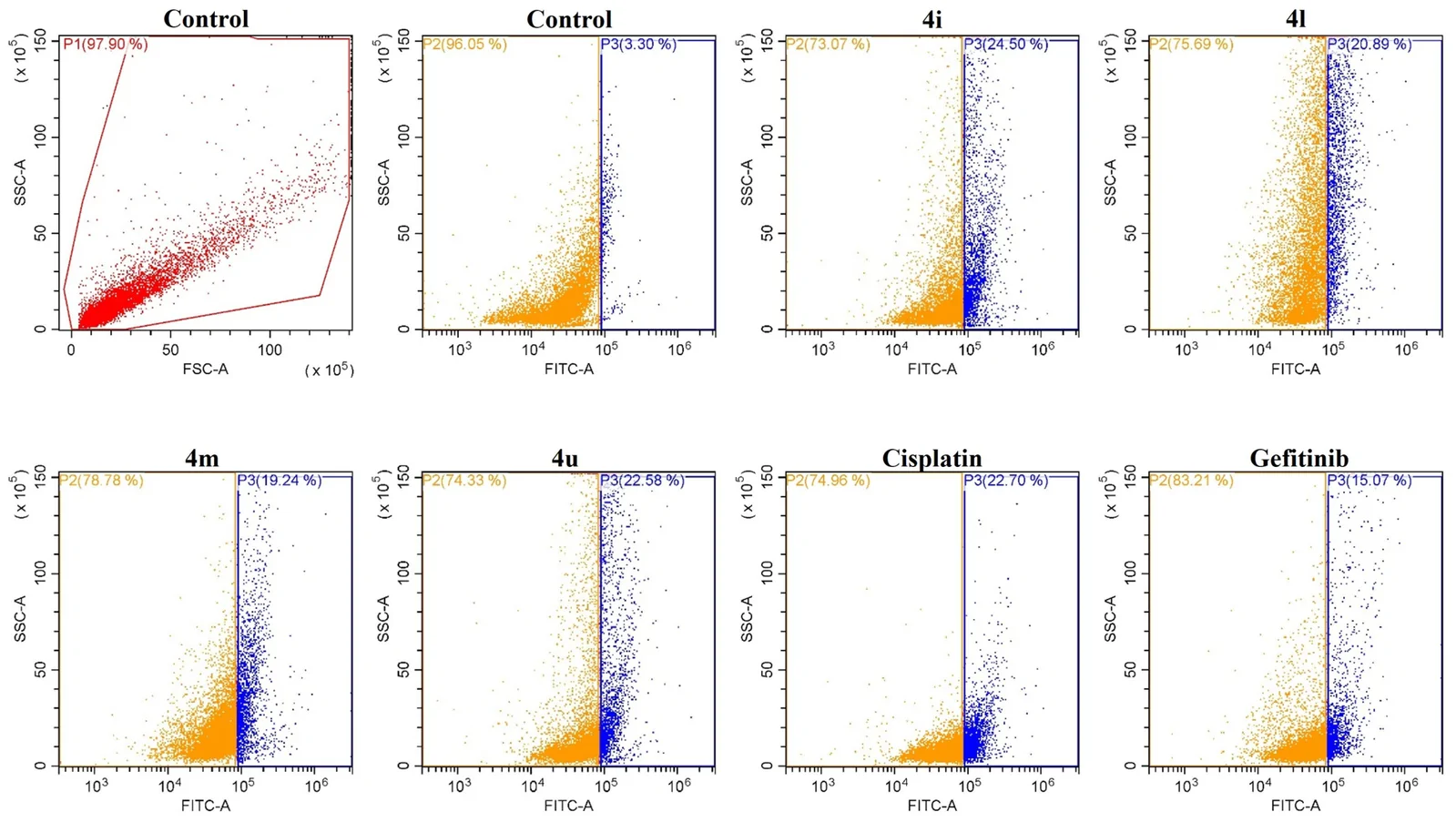
Flow cytometry quadrants analysis of caspase-3 activity in MCF-7 cells treated with compounds **4i**, **4l**, **4m**, **4u**, cisplatin, and gefitinib. P1: caspase-3-positive cells, P2: caspase-3-negative cells.

To investigate caspase-3 activation, MCF-7 cells were incubated for 24 h with compounds **4i**, **4l**, **4m**, **4u** cisplatin and gefitinib with their IC_50_ doses, and the results are presented in Table 5 and Figure 4. After flow cytometry analysis, the percentages of caspase-3-positive cells in treatment with compounds **4i**, **4l**, **4m**, **4u** cisplatin and gefitinib were found to be 24.50%, 20.89%, 19.24%, 22.58%, 22.70%, and 15.07%, respectively, while the percentages of caspase-3-negative cells with these compounds were found to be 73.07%, 75.69%, 78.78%, 74.33%, 74.96%, and 83.21%, respectively (Table 5 and Figure 5). According to these results, the compounds induced caspase-3 activation in MCF-7 cells compared to the control. All compounds surpassed the 15.07% caspase-3 activation exhibited by gefitinib. Furthermore, compound **4i** surpassed cisplatin’s 22.70% caspase-3 activation by 24.50%, while the other compounds showed slightly less caspase-3 activation in MCF-7 compared to cisplatin. Compared to standard drugs and gefitinib, the compounds induced significant caspase-3 activation in the MCF-7 cell line, supporting early apoptosis. Flow cytometry analysis of the apoptotic cell populations showed that the percentage of early apoptotic cells was higher than that of late apoptotic cells, further supporting this finding.

#### 2.2.4. Cell Cycle Analysis

**Figure 6:**
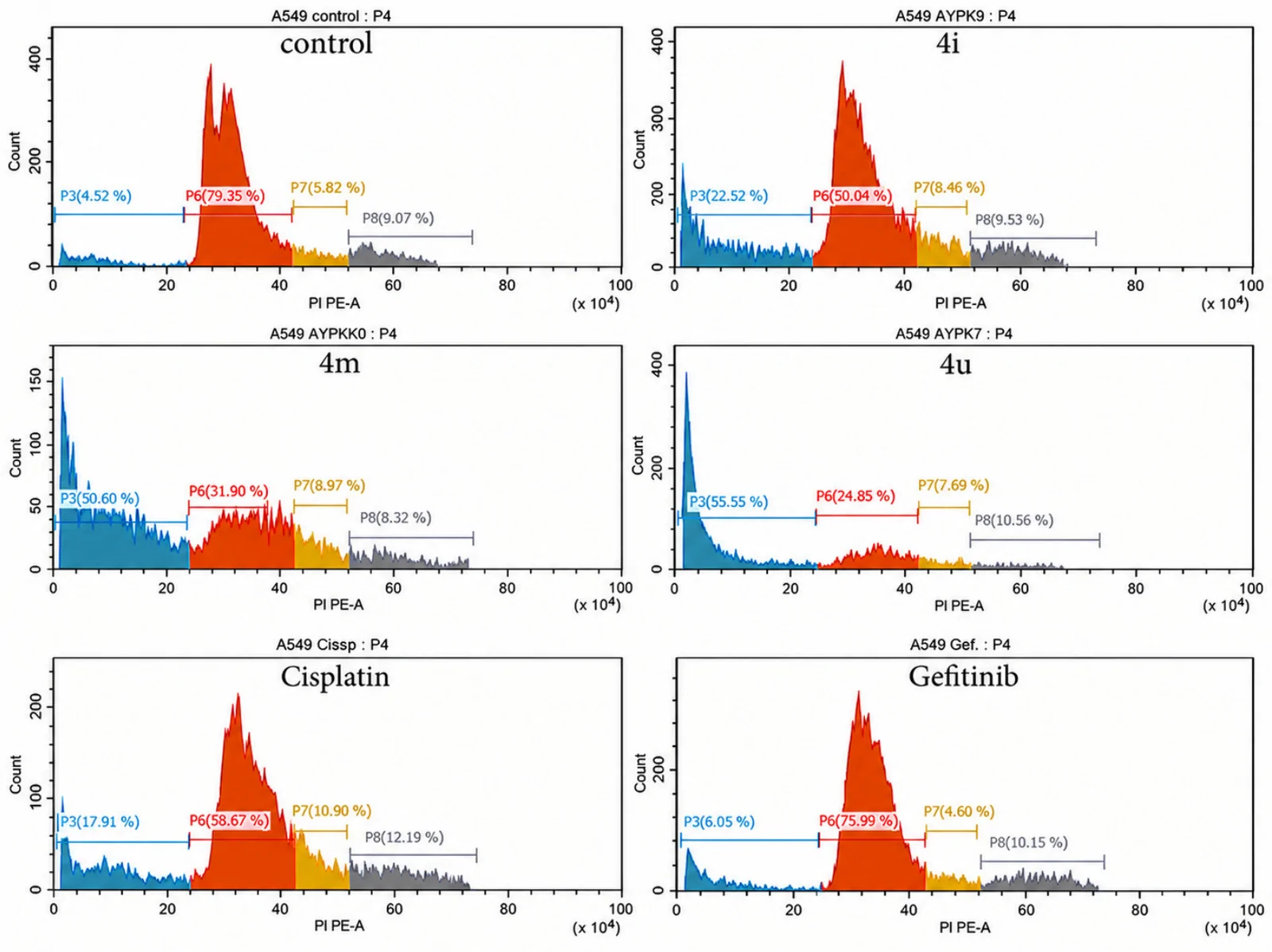
Cell cycle analysis on A549 cell lines.

Cell cycle analysis percentages and images of test compounds and positive controls are given in Table 6 and Figure 6. Compounds **4i**, **4m**, and **4u** had a higher percentage of pre-G (Sub-G0) cells than the positive control cisplatin and gefitinib, thus inducing pre-G apoptosis. These compounds also induced apoptosis by causing S-phase retention, while only gefitinib induced G2/M-phase retention.

**Figure 7:**
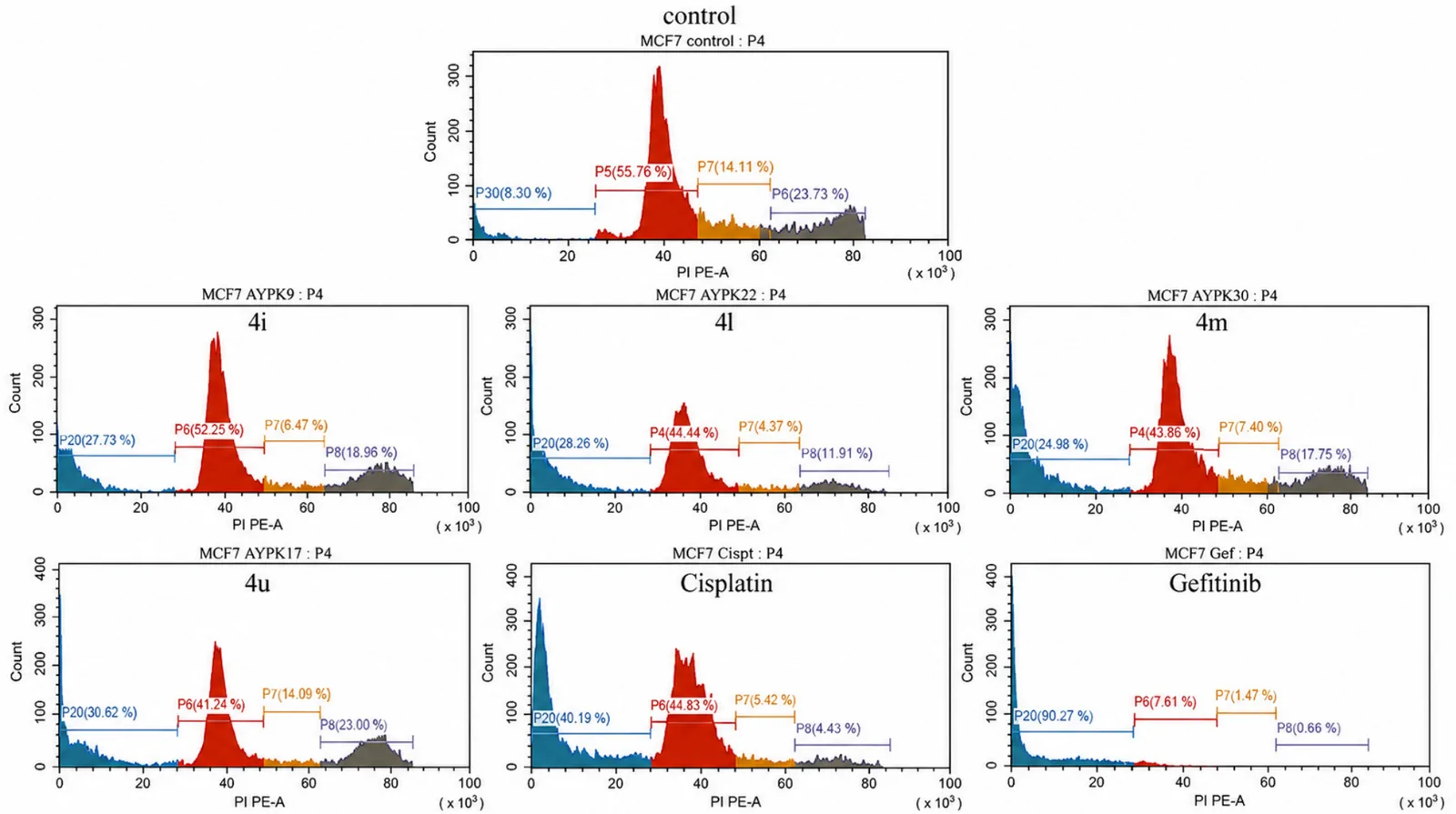
Cell cycle analysis on MCF-7 cell lines.

The percentages and images of MCF-7 cell cycle analysis of the test compounds and positive controls are given in Table 7 and Figure 7. Compounds **4l**, **4m**, and **4u** had a higher percentage of pre-G (Sub-G0) cells compared to the control group, thus inducing pre-G apoptosis. These increases were not as high as those of the positive controls, cisplatin and gefitinib.

#### 2.2.5. EGFR Inhibition

Clinical research has shown that gefitinib and erlotinib offer undeniable benefits in EGFR-targeted therapy for non-small cell lung cancer [47]. Compounds **4i**, **4m**, and **4u**, which selectively showed stronger cytotoxicity than other compounds (**4a**–**4x**), were studied against gefitinib A549, a potent EGFR inhibitor, on the A549 cell line at the determined cytotoxicity dose (IC_50_: 35.58 µM). Cells were treated with the test compounds at three dose levels, namely IC_50_, IC_50_/100, and IC_50_/1000, and the mean ± standard deviation were calculated for all data in this study; the results are presented in Table 8. The positive control, gefitinib, showed an IC_50_ value of 1.86 μM against EGFR. Among the synthesized compounds, **4m** and **4u** demonstrated the greatest inhibitory activity against EGFR (IC_50_: 4.80 μM and IC_50_: 5.40 μM, respectively). Compound **4i** exhibited a moderate EGFR inhibitory value of 9.12 μM.

### 2.3. In Silico Analysis

**Figure 8:**
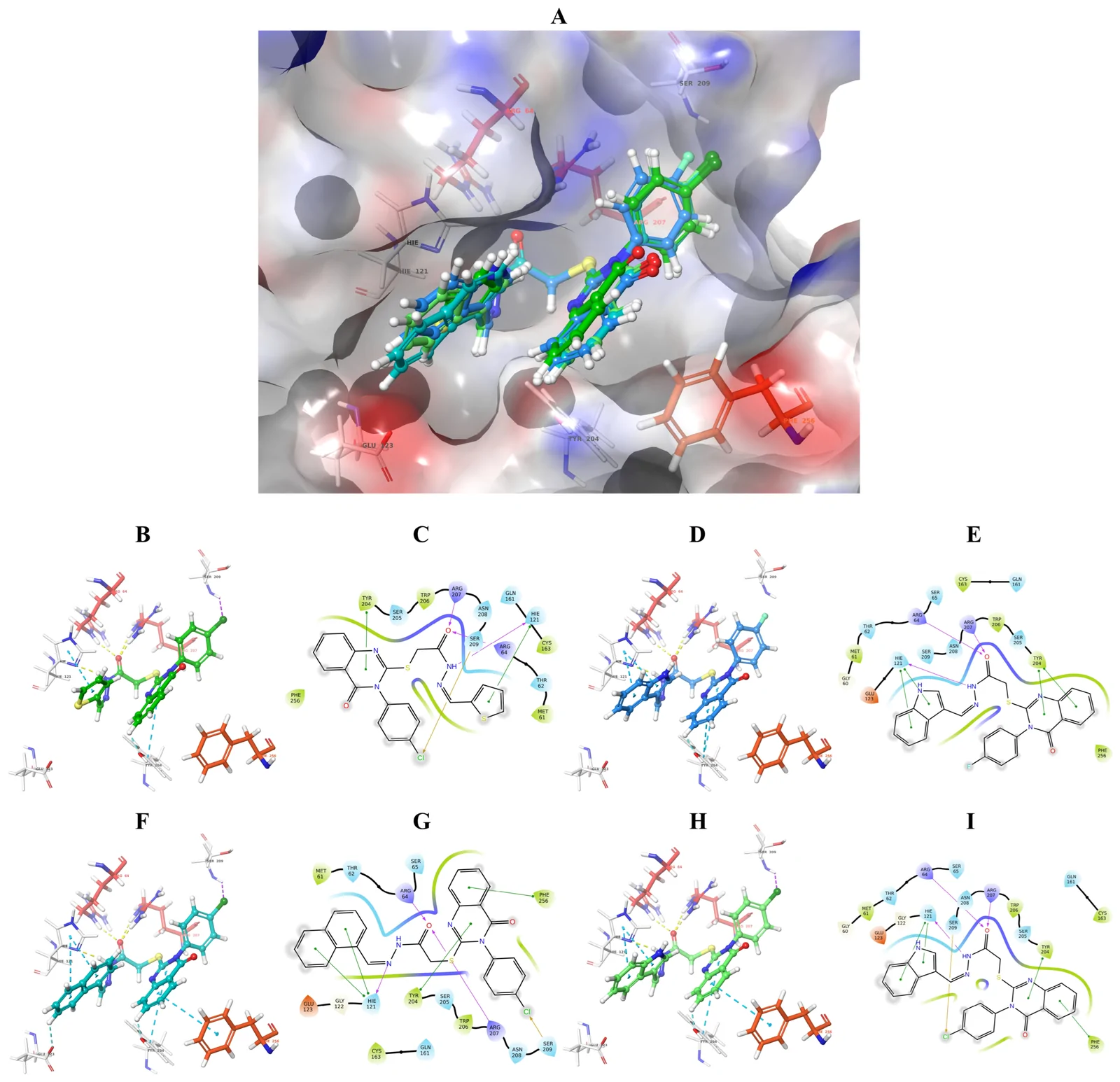
Binding poses at the caspase-3 binding site. (**A**): Superimposed image of active compounds; (**B**,**C**): 3D-2D poses of **4i**; (**D**,**E**): 3D-2D poses of **4l**; (**F**,**G**): 3D-2D poses of **4m**; (**H**,**I**): 3D-2D poses of **4u**.

**Figure 9:**
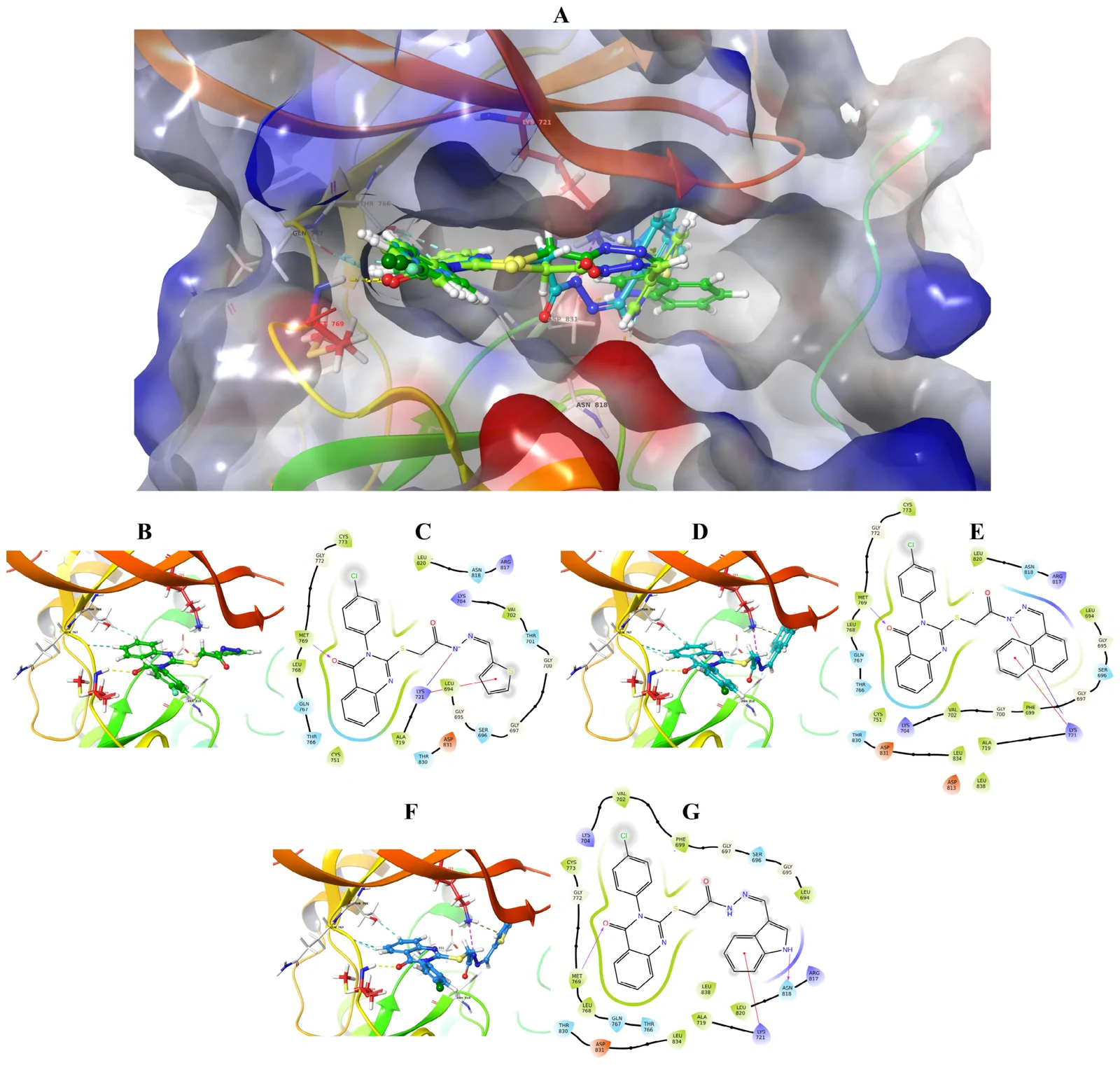
Binding poses at the EGFR binding site. (**A**): Superimposed image of active compounds; (**B**,**C**): 3D-2D poses of **4i**; (**D**,**E**): 3D-2D poses of **4m**; (**F**,**G**): 3D-2D poses of **4u**.

**Figure 10:**
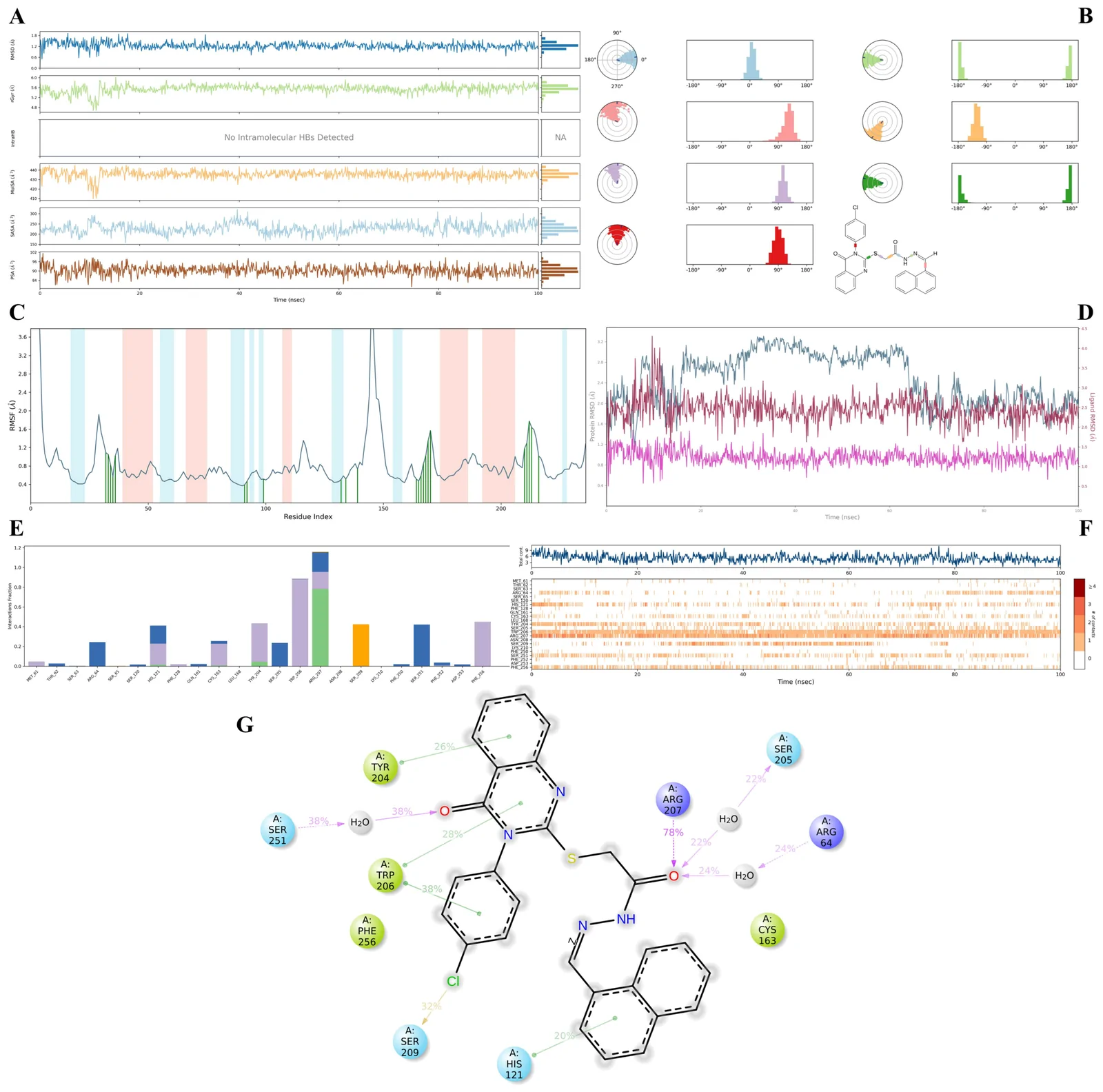
MDS results of **4m**-caspase-3 enzyme. (**A**): Ligand properties; (**B**): ligand torsions; (**C**): RMSF plot; (**D**): RMSD plot for protein Cα (blue), ligand fit protein (red) and ligand fit ligand (purple); (**E**): interaction fractions; (**F**): interaction type and times during simulation time; (**G**): overall bond strength (cutoff: 20%).

**Figure 11:**
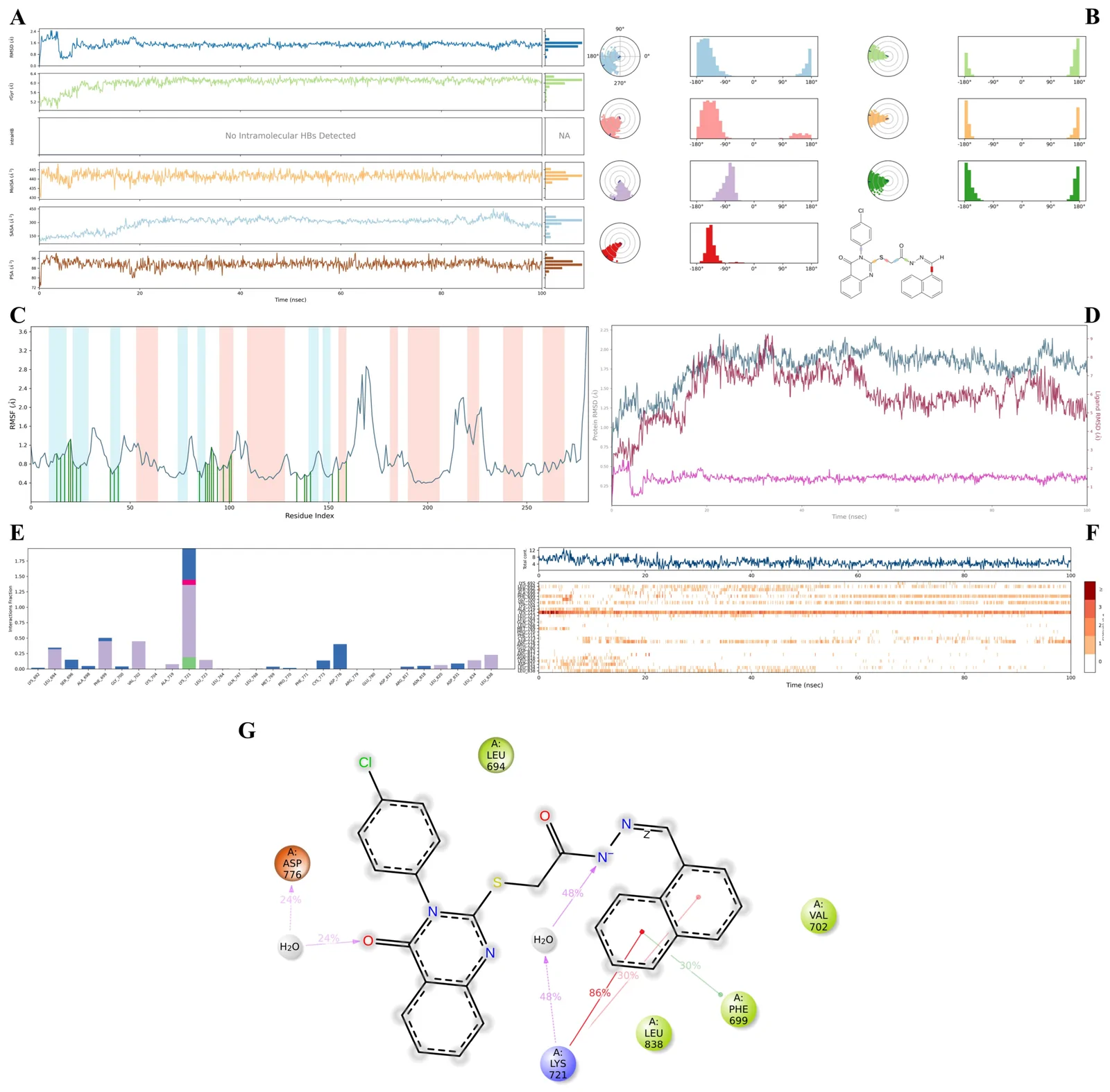
MDS results of **4m**-EGFR. (**A**): Ligand properties; (**B**): ligand torsions; (**C**): RMSF plot; (**D**): RMSD plot for protein Cα (blue), ligand fit protein (red) and ligand fit ligand (purple); (**E**): interaction fractions; (**F**): interaction type and times during simulation time; (**G**): overall bond strength (cutoff: 20%).

Molecular docking (Figure 8 and Figure 9) and molecular dynamics simulation (Figure 10 and Figure 11) studies were performed to elucidate the binding modes of the experimentally identified active compounds (**4i**, **4m**, **4l**, and **4u**) with the target protein.

#### 2.3.1. Molecular Docking

In light of the experimental findings, the binding behaviors and interaction patterns of compounds **4i**, **4l**, **4m**, and **4u** within the caspase-3 binding pocket were analyzed. The obtained binding poses (Figure 8) revealed that the quinazolin-4-one ring system established a π–π interaction with Tyr204, one of the amino acids located in the critical loop region. In the presence of the 4-chlorophenyl group at the 3-position, a halogen bond was formed with Ser209. It was observed that when the variable substituent was bulky, such as indole or naphthalene, an additional π–π interaction with Phe256 could be established, whereas relatively smaller ring systems, such as thiophene, did not interact with this residue. The oxygen atom of the hydrazide carbonyl group formed hydrogen bonds with arginine residues, namely Arg64 and Arg207, suggesting that the compound could be stably accommodated in this region of the enzyme. In addition, the variable substituents (should be aromatic at this case) were found to form π–π interactions with His121, while the nitrogen atom of the hydrazone moiety participated in hydrogen bonding with the same residue. Overall, considering that the experimentally determined caspase-3 activation levels of these compounds were comparable, it can be suggested that their shared core structure adopts a similar binding orientation, as supported by the superimposed poses shown in Figure 8A.

The interactions of the active compounds with EGFR (Figure 9) were further evaluated to elucidate their possible binding modes. A common interaction pattern was observed among the compounds, including a hydrogen bond between the quinazolin-4-one carbonyl oxygen and Met796, as well as aromatic hydrogen bonds formed between Thr766 and Gln767 and the aromatic hydrogens located at the 7- and 6-positions of the quinazoline ring, respectively. These interactions indicate that the compounds adopt highly similar orientations within the enzyme binding pocket.

The phenyl substituent attached to the 3-position of the quinazoline scaffold was oriented toward the outer region of the enzyme cavity. In contrast, the thioether bridge at the 2-position displayed rotational flexibility and extended toward the pocket surrounded by Lys721 and Asp831. This region is located within the ATP binding cleft of the EGFR kinase domain and is functionally relevant for ligand accommodation and stabilization, as Lys721 is associated with the catalytic environment of the kinase domain, while Asp831 is located close to the activation-loop/DFG-related region [44].

The inhibitory activity appeared to be associated more closely with the steric contribution of the substituents on the phenyl ring than with their electron-withdrawing properties. This interpretation is supported by the observation that these groups contributed more effectively to filling the receptor cavity rather than forming dominant electrostatic interactions. Moreover, the rotatable nature of the thioether bridge enabled the ligand to adapt to the receptor binding region, suggesting that the variable substituent plays an important role in receptor accommodation and binding stability.

In this context, bulkier ring systems, including naphthyl, indole, and relatively thiophene moieties, appeared to provide a better fit within the binding pocket. In addition, these groups contributed to complex stabilization through π–cation interactions. Overall, the superior EGFR inhibitory activity of these four compounds compared with the other derivatives may be mainly attributed to the ability of their bulky variable substituents to occupy the receptor cavity more effectively and to stabilize the ligand–EGFR complex.

#### 2.3.2. Molecular Dynamics Simulation

To investigate the behavior of the quinazolin-4-one and acyl arylhydrazone moieties within the caspase-3 binding pocket, the enzyme complex of caspase-3 with compound **4m**, the most active analogue, was selected for molecular dynamics simulation (MDS). According to the simulation stability results (Figure 10A,C,D), low fluctuations were observed in the RMSD and Rg values of the ligand, while the protein RMSD remained within the range of 1–3 Å. The reduced fluctuation in the protein RMSD after 63 ns indicates that the system reached a more stable state. The ligand torsion plot (Figure 10B) showed that the ligand maintained certain torsional conformations throughout the simulation. The observation of narrow and unimodal angular distributions for most rotatable bonds suggests that the ligand was conformationally restricted within the binding pocket and exhibited a stable binding geometry. The distributions observed around ±180° most likely reflect boundary effects arising from the periodic representation of anti/trans conformations. Overall, these findings support that the ligand did not undergo marked conformational dispersion or destabilization during the simulation.

When the binding interactions of the complex were examined (Figure 10E–G), frequent interactions were observed between the oxygen atom of the acetamide moiety and Arg207 throughout the simulation, including direct and water-mediated hydrogen bonds, as well as π-related interactions. The same moiety also formed occasional water-mediated hydrogen bonds with Arg64. In addition, the quinazolin-4-one and 4-chlorophenyl rings frequently established π–π interactions with Trp206 throughout the simulation. A halogen bond between the 4-chlorophenyl group and Ser209 was observed intermittently, while the same moiety occasionally formed π–π interactions with Phe256. Furthermore, occasional water-mediated hydrogen bonding was detected between the 4-oxoquinazoline moiety and Ser251, and intermittent π–π interactions were observed between the naphthyl group and His121.

Among these interactions, those other than the interactions with Phe256 appeared to make a substantial contribution to complex stability. Although interactions with Phe256 were occasionally observed, their interaction strength was calculated to be below 20%. Overall, most of the interactions observed in the docking study were largely maintained during the simulation. However, the hydrogen bond observed between the hydrazone nitrogen and His121 in the docking pose appeared to weaken during the simulation.

Taken together, and consistent with previous studies [44], these findings may suggest that the acetamide moiety effectively interacts with Arg207 and Arg64 and may, therefore, represent an important pharmacophoric group. In addition, the quinazolinone and naphthyl rings attached to this moiety may contribute to differences in caspase-3 activation potency depending on their ability to stabilize amino acid residues located in the loop regions of the enzyme.

The **4m**–EGFR complex was selected as a representative model of the active compounds to investigate the plausible binding mechanism and conformational behavior of quinazolin-4-one–hydrazide hybrids within the EGFR binding pocket. Results of the MDS of **4m**-EGFR complex (Figure 11) indicated that, based on the ligand Rg and RMSD plots, **4m** underwent repositioning relative to EGFR within the first 10 ns and subsequently exhibited only minimal fluctuations, without any pronounced instability. In addition, the protein RMSD values remained within the range of 1–3 Å, while the RMSF plot showed that the residues located in the interacting loop regions, indicated by the green lines in the white region, displayed fluctuations below 1 Å, suggesting that these regions were stabilized during the simulation (Figure 11A,C,D) [48,49].

Following ligand repositioning, the interaction between the quinazolin-4-one carbonyl oxygen and the key residue Met769 became weaker, whereas the interaction with Asp776, located in the same binding region, became more prominent and occurred with higher frequency. Nevertheless, the L-torsion analysis (Figure 11B) indicated that the ligand did not undergo completely free rotation throughout the simulation but instead adopted preferred torsional angles. The narrow angular distributions observed for some bonds suggest that the ligand was conformationally restricted within the binding pocket and maintained its overall binding geometry. In this study, the distributions observed around ±180° were interpreted as an indication that the ligand preferentially preserved a trans/anti-like torsional arrangement during the simulation, rather than exhibiting unrestricted rotational behavior. Therefore, these results further support the generally stable conformational behavior of the ligand within the binding pocket. Throughout the simulation, the most frequent and persistent interactions were observed between Lys721 and the naphthyl group through π–cation interactions, as well as between Lys721 and the hydrazide N1 atom through water-mediated hydrogen bonds. Another notable interaction was the π–π interaction formed between Phe699 and the naphthyl ring (Figure 11E–G).

## 3. Materials and Methods

### 3.1. Chemistry

All chemicals used in the syntheses were sourced either from Merck Chemicals (Merck KGaA, Darmstadt, Germany) or Sigma-Aldrich Chemicals (Sigma-Aldrich Corp., St. Louis, MO, USA). Reaction monitoring and compound purity were performed using thin-layer chromatography (TLC) on silica gel 60 F_254_ aluminum plates supplied by Merck (Darmstadt, Germany). The melting points of the final compounds were determined using an MP90 digital melting point scale (Mettler Toledo, Columbus, OH, USA). IR spectra were recorded using a Shimadzu IR spectrometer (Shimadzu, Kyoto, Japan). ^1^H NMR and ^13^C NMR spectra were recorded in DMSO-*d*_6_ solvent using a Bruker 300 MHz digital FT NMR spectrometer (Bruker Bioscience, Billerica, MA, USA). Mass spectroscopy analyses were performed using high-resolution mass spectrometric (HRMS) studies, utilizing an LC/MS-IT-TOF system (Shimadzu, Kyoto, Japan).

#### 3.1.1. Synthesis of the Compounds

##### Synthesis of 2-Mercapto-3-(substituted phenyl)-3H-substituted quinazolin-4-one Derivatives (**1a**–**1d**)

The appropriate anthranilic acid derivatives (1 eq) and the appropriate isothiocyanate derivatives (1 eq) were boiled in absolute ethanol containing a catalytic amount of triethylamine (1.5 eq). After 2 h, the reaction was monitored by TLC and completed. The crude product was isolated by filtration and washing with ethanol.

##### Synthesis of Ethyl Ester Derivatives of [4-Oxo-3-(substituted phenyl)-3,4-dihydro-(substituted quinazolin-2-yl)thio]acetic Acid (**2a**–**2d**)

2-Mercapto-3-(substituted phenyl)-3*H*-substituted quinazolin-4-one derivatives (1eq) (**1a**–**1d**) obtained in the first step were boiled for 6 h in acetone with ethyl iodoacetate (1.2 eq) and potassium carbonate (1 eq). Reaction completion was confirmed by thin-layer chromatography, and the crude product was precipitated with addition of water and separated by filtration.

##### Synthesis of 2-[[4-Oxo-3-(substituted phenyl)-3,4-dihydro-(substituted quinazolin-2-yl)]thio]acetohydrazide Derivatives (**3a**–**3d**)

The [4-oxo-3-(substituted phenyl)-3,4-dihydro-(substituted quinazolin-2-yl)thio]acetic acid ethyl ester derivatives (**2a**–**2d**) obtained in the second step (1 eq) were reacted with excessive amount hydrazine hydrate (3 eq) by boiling overnight in ethanol. Reaction completion was monitored by TLC, and the crude product was precipitated and washed with ethanol [50].

##### Synthesis of 2-[[4-Oxo-3-(substituted phenyl)-3,4-dihydro-(substituted quinazolin-2-yl)]thio]-N′-(aryl/heteroaryl methylene)acetohydrazide Derivatives (**4a**–**4x**)

The 2-[[4-oxo-3-(substituted phenyl)-3,4-dihydro-(substituted quinazolin-2-yl)]thio]acetohydrazide (1 eq) derivatives (**3a**–**3d**) obtained in the third step were boiled with selected aldehyde derivatives (1.1 eq) in ethanol for 1 h. The final compounds were gained by precipitating in the reaction medium or by precipitating when left standing. The crude products were recrystallized from ethanol.

##### 2-((3-(4-Fluorophenyl)-6-methyl-4-oxo-3,4-dihydroquinazolin-2-yl)thio)-*N*′-(pyridin-4-ylmethylene)acetohydrazide (**4a**)

C_23_H_18_FN_5_O_2_S, m.p.: 270–271 °C, yield: 62%. ^1^H-NMR (300 MHz, DMSO-*d*_6_): δ: 2.39 and 2.44 (3H, 2s, Quinazolinone-CH_3_), 4.02 and 4.49 (2H, 2s, S-CH_2_), 7.27–7.67 (8H, m, Ar-CH), 7.96 (1H, d, *J*: 7.92 Hz, Ar-CH), 8.05 and 8.27 (1H, 2s, Ar-CH), 8.62–8.65 (2H, m, Ar-CH), 11.95 (1H, s, NH). ^13^C-NMR: (75 MHz, DMSO-*d*_6_): δ: 21.8, 34.4, 117.0 (*J_CF_*: 22.8 Hz), 117.5, 121.3, 121.4, 126.1, 127.0, 127.9, 132.4 (*J_CF_*: 9.2 Hz), 141.4, 141.7, 144.7, 146.0, 147.6, 150.7, 157.2, 161.1, 163.1 (*J_CF_*: 245.4 Hz), 164.5, 169.7. HRMS (*m*/*z*): [M+H]^+^: calculated: 448.1238, found: 448.1240.

##### 2-((3-(4-Fluorophenyl)-6-methyl-4-oxo-3,4-dihydroquinazolin-2-yl)thio)-*N*′-(pyridin-2-ylmethylene)acetohydrazide (**4b**)

C_23_H_18_FN_5_O_2_S, m.p.: 235–236 °C, yield: 70%. ^1^H-NMR (300 MHz, DMSO-*d*_6_): δ: 2.39 and 2.45 (3H, 2s, Quinazolinone-CH_3_), 4.01 and 4.48 (2H, 2s, S-CH_2_), 7.28–7.33 (1H, m, Ar-CH), 7.39–7.46 (4H, m, Ar-CH), 7.54–7.59 (2H, m, Ar-CH), 7.94–7.97 (1H, m, Ar-CH), 8.10–8.32 (2H, m, Ar-CH), 8.60–8.62 (1H, m, Ar-CH), 8.84–8.88 (1H, m, Ar-CH), 11.85 (1H, s, NH). ^13^C-NMR: (75 MHz, DMSO-*d*_6_): δ: 21.8, 34.5, 116.9 (*J_CF_*: 22.8 Hz), 124.4, 126.1, 126.9, 127.9, 130.5, 132.4 (*J_CF_*: 9.3 Hz), 133.8, 133.9, 141.1, 144.5, 146.0, 147.6, 149.0, 149.2, 151.0, 151.2, 161.1, 163.1 (*J_CF_*: 245.5 Hz), 164.2, 169.4. HRMS (*m*/*z*): [M+H]^+^: calculated: 448.1238, found: 448.1236.

##### 2-((3-(4-Fluorophenyl)-6-methyl-4-oxo-3,4-dihydroquinazolin-2-yl)thio)-*N*′-(thiophene-2-ylmethylene)acetohydrazide (**4c**)

C_22_H_17_FN_4_O_2_S_2_, m.p.: 254–255 °C, yield: 65%. ^1^H-NMR (300 MHz, DMSO-*d*_6_): δ: 2.41 and 2.46 (3H, 2s, Quinazolinone-CH_3_), 3.99 and 4.41 (2H, 2s, S-CH_2_), 7.13–7.17 (1H, m, Ar-CH), 7.28–7.33 (2H, m, Ar-CH), 7.40–7.48 (3H, m, Ar-CH), 7.55–7.60 (2H, m, Ar-CH), 7.66 (1H, d, *J*: 4.89 Hz, Ar-CH), 7.96–7.99 (1H, m, Ar-CH), 8.25 and 8.50 (1H, 2s, Ar-CH), 11.65 (1H, s, NH). ^13^C-NMR: (75 MHz, DMSO-*d*_6_): δ: 21.8, 34.6, 35.8, 117.0 (*J_CF_*: 23.0 Hz), 126.2, 127.9 (*J_CF_*: 9.4 Hz), 128.4, 129.0, 129.5, 131.0, 131.6, 132.4 (*J_CF_*: 9.3 Hz), 139.1, 142.5, 146.0, 147.7, 161.1, 163.1 (*J_CF_*: 245.3 Hz), 163.8, 168.9. HRMS (*m*/*z*): [M+H]^+^: calculated: 453.0850, found: 453.0856.

##### *N*′-((1*H*-Pyyrol-2-yl)methylene)-2-((3-(4-fluorophenyl)-6-methyl-4-oxo-3,4-dihydroquinazolin-2-yl)thio)acetohydrazide (**4d**)

C_22_H_18_FN_5_O_2_S, m.p.: 242–243 °C, yield: 75%. ^1^H-NMR (300 MHz, DMSO-*d*_6_): δ: 2.41 and 2.47 (3H, 2s, Quinazolinone-CH_3_), 3.98 and 4.43 (2H, 2s, S-CH_2_), 6.11–6.16 (1H, m, Ar-CH), 6.46–6.47 (1H, m, Ar-CH), 6.92 (1H, dd, *J*_1_: 1.26 Hz, *J*_2_: 16.26 Hz, Ar-CH), 7.28–7.32 (2H, m, Ar-CH), 7.41–7.47 (2H, m, Ar-CH), 7.54–7.60 (2H, m, Ar-CH), 7.90 and 8.09 (1H, 2s, Ar-CH), 7.97 (1H, dd, *J*_1_: 3.54 Hz, *J*_2_: 8.03 Hz, Ar-CH), 11.32 and 11.40 (1H, 2s, NH), 11.38 and 11.49 (1H, 2s, NH). ^13^C-NMR: (75 MHz, DMSO-*d*_6_): δ: 21.8, 21.9, 34.7, 109.7, 112.9, 113.9, 117.0 (*J_CF_*: 22.9 Hz), 122.4, 123.1, 127.0, 127.5, 127.8, 128.0, 132.4 (*J_CF_*: 8.2 Hz), 136.7, 140.4, 146.0, 147.7, 161.1, 163.1 (*J_CF_*: 242.8 Hz), 168.4. HRMS (*m*/*z*): [M+H]^+^: calculated: 436.1238, found: 436.1238.

##### 2-((4-Oxo-3-phenyl-3,4-dihydroquinazolin-2-yl)thio)-*N*′-(thiophen-2-ylmethylene)acetohydrazide (**4e**)

C_21_H_16_N_4_O_2_S_2_, m.p.: 255–256 °C, yield: 66%. ^1^H-NMR (300 MHz, DMSO-*d*_6_): δ: 3.99 and 4.42 (2H, 2s, S-CH_2_), 7.13–7.16 (1H, m, Ar-CH), 7.49–7.65 (9H, m, Ar-CH), 7.80–7.88 (1H, m, Ar-CH), 8.10 (1H, d, *J*: 7.44 Hz, Ar-CH), 8.25 and 8.49 (1H, 2s, Ar-CH), 11.65 and 11.74 (1H, 2s, NH). ^13^C-NMR: (75 MHz, DMSO-*d*_6_): δ: 34.7, 35.8, 120.0, 126.5, 127.1, 128.4, 129.0, 129.9, 130.0, 130.5, 131.0, 131.6, 135.4, 136.3, 139.0, 139.3, 142.3, 147.6, 157.5, 163.9, 168.9. HRMS (*m*/*z*): [M+H]^+^: calculated: 421.0787, found: 421.0794.

##### *N*′-((1*H*-Pyrrol-2-yl)methylene)-2-((4-oxo-3-phenyl-3,4-dihydroquinazolin-2-yl)thio)acetohydrazide (**4f**)

C_21_H_17_N_5_O_2_S, m.p.: 230–231 °C, yield: 61%. ^1^H-NMR (300 MHz, DMSO-*d*_6_): δ: 3.99 and 4.45 (2H, 2s, S-CH_2_), 6.13–6.16 (1H, m, Ar-CH), 6.46–6.49 (1H, m, Ar-CH), 6.89–6.95 (1H, m, Ar-CH), 7.45–7.64 (7H, m, Ar-CH), 7.79–7.90 (2H, m, Ar-CH), 8.09–8.11 (1H, m, Ar-CH), 11.34 and 11.44 (1H, 2s, NH), 11.38 and 11.49 (1H, 2s, NH). ^13^C-NMR: (75 MHz, DMSO-*d*_6_): δ: 34.7, 36.0, 109.7, 112.9, 113.9, 122.4, 123.0, 126.5, 127.1, 127.5, 129.9, 130.1, 130.5, 135.4, 135.4, 136.2, 136.4, 136.7, 147.6, 163.2, 168.5. HRMS (*m*/*z*): [M+H]^+^: calculated: 404.1176, found: 404.1156.

##### 2-((3-(4-Chlorophenyl)-4-oxo-3,4-dihydroquinazolin-2-yl)thio)-*N*′-(pyridin-4-ylmethylene)acetohydrazide (**4g**)

C_22_H_16_ClN_5_O_2_S, m.p.: 291–292 °C, yield: 63%. ^1^H-NMR (300 MHz, DMSO-*d*_6_): δ: 4.07 and 4.53 (2H, 2s, S-CH_2_), 7.50–7.73 (8H, m, Ar-CH), 7.80–7.89 (1H, m, Ar-CH), 8.06–8.29 (2H, m, Ar-CH), 8.64–8.67 (2H, m, Ar-CH), 12.01 (1H, s, NH). ^13^C-NMR: (75 MHz, DMSO-*d*_6_): δ: 34.5, 35.9, 120.0, 121.2, 121.5, 126.5, 126.6, 127.1, 130.2, 132.0, 135.1, 135.2, 135.5, 141.4, 141.7, 141.8, 147.5, 150.7, 156.8, 157.0, 161.1, 169.7. HRMS (*m*/*z*): [M+H]^+^: calculated: 450.0786, found: 450.0787.

##### 2-((3-(4-Chlorophenyl)-4-oxo-3,4-dihydroquinazolin-2-yl)thio)-*N*′-(pyridin-2-ylmethylene)acetohydrazide (**4h**)

C_22_H_16_ClN_5_O_2_S, m.p.: 223–224 °C, yield: 64%. ^1^H-NMR (300 MHz, DMSO-*d*_6_): δ: 4.04 and 4.51 (2H, 2s, S-CH_2_), 7.41–7.72 (7H, m, Ar-CH), 7.78–7.95 (3H, m, Ar-CH), 8.08 and 8.29 (1H, 2s, Ar-CH), 8.11 (1H, m, Ar-CH), 8.62–8.63 (1H, m, Ar-CH), 11.88 and 12.01 (1H, 2s, NH). ^13^C-NMR: (75 MHz, DMSO-*d*_6_): δ: 34.5, 35.9, 120.1, 120.4, 124.8, 126.5, 126.6, 127.1, 130.2, 132.0, 135.2, 135.5, 137.3, 144.3, 147.4, 147.5, 150.0, 153.4, 153.5, 161.1, 164.4, 169.4. HRMS (*m*/*z*): [M+H]^+^: calculated: 450.0786, found: 450.0770.

##### 2-((3-(4-Chlorophenyl)-4-oxo-3,4-dihydroquinazolin-2-yl)thio)-*N*′-(thiophen-2-ylmethylene)acetohydrazide (**4i**)

C_21_H_15_ClN_4_O_2_S_2_, m.p.: 261–262 °C, yield: 74%. ^1^H-NMR (300 MHz, DMSO-*d*_6_): δ: 4.03 and 4.46 (2H, 2s, S-CH_2_), 7.16–7.18 (1H, m, Ar-CH), 7.49–7.74 (8H, m, Ar-CH), 7.82–7.90 (1H, m, Ar-CH), 8.12 (1H, d, *J*: 7.65 Hz, Ar-CH), 8.27 and 8.51 (1H, 2s, Ar-CH), 11.71 (1H, s, NH). ^13^C-NMR: (75 MHz, DMSO-*d*_6_): δ: 34.7, 35.8, 120.0, 126.5, 127.1, 128.4, 129.0, 129.5, 130.1, 131.1, 131.6, 132.0, 135.5, 139.1, 139.3, 142.4, 147.6, 157.1, 161.1, 163.8, 168.9. HRMS (*m*/*z*): [M+H]^+^: calculated: 455.0398, found: 455.0394.

##### *N*′-((1*H*-Pyrrol-2-yl)methylene)-2-((3-(4-chlorophenyl)-4-oxo-3,4-dihydroquinazolin-2-yl)thio)acetohydrazide (**4j**)

C_21_H_16_ClN_5_O_2_S, m.p.: 233–234 °C, yield: 72%. ^1^H-NMR (300 MHz, DMSO-*d*_6_): δ: 3.99 and 4.45 (2H, 2s, S-CH_2_), 6.11–6.15 (1H, m, Ar-CH), 6.44–6.49 (1H, m, Ar-CH), 6.88–6.95 (1H, m, Ar-CH), 7.44–7.60 (4H, m, Ar-CH), 7.69 (2H, d, *J*: 8.64 Hz, Ar-CH), 7.78–7.86 (1H, m, Ar-CH), 7.89 (1H, s, Ar-CH), 8.07–8.10 (1H, m, Ar-CH), 11.34 and 11.43 (1H, 2s, NH) 11.38 and 11.48 (1H, 2s, NH). ^13^C-NMR: (75 MHz, DMSO-*d*_6_): δ: 34.9, 36.1, 109.7, 112.8, 114.0, 120.0, 122.4, 123.0, 126.5, 127.2, 127.5, 130.1, 132.0, 135.2, 136.7, 147.6, 157.0, 157.1, 161.1, 163.1, 168.3. HRMS (*m*/*z*): [M+H]^+^: calculated: 438.0786, found: 438.0774.

##### 2-((3-(4-Chlorophenyl)-4-oxo-3,4-dihydroquinazolin-2-yl)thio)-*N*′-(furan-2-ylmethylene)acetohydrazide (**4k**)

C_21_H_15_ClN_4_O_3_S, m.p.: 238–239 °C, yield: 70%. ^1^H-NMR (300 MHz, DMSO-*d*_6_): δ: 3.99 and 4.42 (2H, 2s, S-CH_2_), 6.62–6.65 (1H, m, Ar-CH), 6.91–6.93 (1H, m, Ar-CH), 7.445–7.61 (4H, m, Ar-CH), 7.67–7.70 (2H, m, Ar-CH), 7.78–7.87 (2H, m, Ar-CH), 7.95 and 8.15 (1H, 2s, Ar-CH), 8.08 (1H, d, *J*: 7.59 Hz, Ar-CH), 11.62 and 11.73 (1H, 2s, NH). ^13^C-NMR: (75 MHz, DMSO-*d*_6_): δ: 34.8, 35.9, 112.7, 114.1, 114.3, 120.0, 126.5, 127.1, 130.2, 132.0, 134.1, 135.3, 135.5, 137.0, 145.5, 147.6, 149.5, 157.1, 161.1, 163.9, 168.9. HRMS (*m*/*z*): [M+H]^+^: calculated: 439.0626, found: 439.0625.

##### *N*′-((1*H*-Indol-3-yl)methylene)-2-((3-(4-chlorophenyl)-4-oxo-3,4-dihydroquinazolin-2-yl)thio)acetohydrazide (**4l**)

C_25_H_18_ClN_5_O_2_S, m.p.: 290–291 °C, yield: 68%. ^1^H-NMR (300 MHz, DMSO-*d*_6_): δ: 4.02 and 4.61 (2H, 2s, S-CH_2_), 7.11–7.26 (2H, m, Ar-CH), 7.42–7.51 (2H, m, Ar-CH), 7.57–7.60 (3H, m, Ar-CH), 7.68 (2H, d, *J*: 8.67 Hz, Ar-CH), 7.82–7.88 (2H, m, Ar-CH), 8.08–8.12 (1H, m, Ar-CH), 8.23 (1H, s, Ar-CH), 8.25 and 8.41 (1H, 2s, Ar-CH), 11.39 and 11.42 (1H, 2s, NH), 11.61 (1H, s, NH). ^13^C-NMR: (75 MHz, DMSO-*d*_6_): δ: 36.0, 111.9, 112.5, 120.0, 120.9, 122.1, 123.2, 124.5, 126.4, 126.5, 127.1, 130.1, 131.2, 132.0, 135.2, 135.3, 135.5, 137.6, 141.6, 144.4, 147.7, 157.4, 161.2, 162.9, 168.2. HRMS (*m*/*z*): [M+H]^+^: calculated: 488.0942, found: 488.0942.

##### 2-((3-(4-Chlorophenyl)-4-oxo-3,4-dihydroquinazolin-2-yl)thio)-*N*′-(naphthalen-1-ylmethylene)acetohydrazide (**4m**)

C_27_H_19_ClN_4_O_2_S, m.p.: 251–252 °C, yield: 65%. ^1^H-NMR (300 MHz, DMSO-*d*_6_): δ: 4.08 and 4.58 (2H, 2s, S-CH_2_), 7.44–7.51 (2H, m, Ar-CH), 7.57–7.72 (7H, m, Ar-CH), 7.76–7.84 (1H, m, Ar-CH), 7.90 (1H, d, *J*: 7.17 Hz, Ar-CH), 8.00–8.10 (3H, m, Ar-CH), 8.73 (1H, s, Ar-CH), 8.84–8.89 (1H, m, Ar-CH), 11.74 and 11.90 (1H, 2s, NH). ^13^C-NMR: (75 MHz, DMSO-*d*_6_): δ: 35.1, 35.9, 120.0, 124.3, 124.8, 126.0, 126.5, 126.8, 127.1, 127.8, 128.7, 129.4, 129.8, 130.2, 130.5, 130.9, 132.0, 134.0, 135.2, 135.5, 143.9, 147.3, 147.6, 157.1, 161.1, 164.0, 169.1. HRMS (*m*/*z*): [M+H]^+^: calculated: 499.0990, found: 499.1000.

##### 2-((3-(4-Chlorophenyl)-4-oxo-3,4-dihydroquinazolin-2-yl)thio)-*N*′-(naphthalen-2-ylmethylene)acetohydrazide (**4n**)

C_27_H_19_ClN_4_O_2_S, m.p.: 270–271 °C, yield 72%. IR (ATR) υ_max_ (cm^−1^): 3163 (aromatic C-H), 2990 (aliphatic C-H), 1692 (CON-H), 1680, 1672, 1666 (CON-H), 1547, 1543, 1537, 1470 (C=C, C=N), 1346, 1261, 1256, 1223, 1215, 1201 (C-N, C-O), 831, 808, 766, 745, 733 (benzene ring out-of-plane deformation band). ^1^H-NMR (400 MHz, DMSO-*d*_6_): δ: 4.05 and 4.55 (2H, 2s, S-CH_2_), 7.48–7.68 (8H, m, Ar-CH), 7.80–7.99 (5H, m, Ar-CH), 8.08–8.16 (2H, m, Ar-CH), 8.23 and 8.42 (1H, 2s, Ar-CH), 11.74 and 11.85 (1H, 2s, NH). ^13^C-NMR: (100 MHz, DMSO-*d*_6_): δ: 34.7, 120.0, 122.7, 123.0, 126.5, 127.1, 127.3, 127.6, 128.2, 128.8, 129.0, 129.2, 130.1, 132.0, 132.2, 133.3, 134.1, 135.2, 135.5, 143.9, 147.5, 157.1, 161.1, 161.1, 164.0, 169.2.

##### 2-((3-(4-Chlorophenyl)-4-oxo-3,4-dihydroquinazolin-2-yl)thio)-*N*′-(cyclohexylmethylene)acetohydrazide (**4o**)

C_23_H_23_ClN_4_O_2_S, m.p.: 238–239 °C, yield 68%. IR (ATR) υ_max_ (cm^−1^): 3109 (aromatic C-H), 2947 (aliphatic C-H), 1680 (CON-H), 1548, 1470 (C=C, C=N), 1263, 1202(C-N, C-O), 769 (benzene ring out-of-plane deformation band). ^1^H-NMR (400 MHz, DMSO-*d*_6_): δ: 1.16–1.30 (5H, m, cyclohexyl CH), 1.61–1.71 (5H, m, cyclohexyl CH), 2.21 (1H, s, cyclohexyl CH), 3.91 and 4.32 (2H, 2s, S-CH_2_), 7.27 (1H, s, Ar-CH), 7.46–7.56 (4H, m, Ar-CH), 7.67–7.68 (2H, m, Ar-CH), 7.84 (1H, s, Ar-CH), 8.08 (1H, d, *J*: 6.88 Hz, Ar-CH), 11.18 and 11.27 (1H, 2s, NH). ^13^C-NMR: (100 MHz, DMSO-*d*_6_): δ: 25.4, 25.4, 25.9, 26.0, 30.0, 30.1, 34.7, 35.8, 119.9, 126.5, 127.1, 130.1, 130.1, 131.9, 135.2, 135.4, 147.5, 151.7, 155.2, 157.1, 161.1, 163.4, 168.7.

##### 2-((3-(4-Fluorophenyl)-4-oxo-3,4-dihydroquinazolin-2-yl)thio)-*N*′-(pyridin-4-ylmethylene)acetohydrazide (**4p**)

C_22_H_16_FN_5_O_2_S, m.p.: 245–246 °C, yield: 63%. ^1^H-NMR (300 MHz, DMSO-*d*_6_): δ: 4.04 and 4.50 (2H, 2s, S-CH_2_), 7.42–7.49 (4H, m, Ar-CH), 7.57–7.65 (4H, m, Ar-CH), 7.77–7.85 (1H, m, Ar-CH), 8.05–8.27 (2H, m, Ar-CH), 8.62–8.64 (2H, m, Ar-CH), 11.95 and 12.07 (1H, 2s, NH). ^13^C-NMR: (75 MHz, DMSO-*d*_6_): δ: 19.0, 34.5, 35.9, 117.0 (*J_CF_*: 22.8 Hz), 120.0, 121.3, 126.5, 127.1, 132.4 (*J_CF_*: 9.2 Hz), 135.5, 141.4, 141.7, 144.7, 147.5, 150.7, 157.4, 161.2, 163.1 (*J_CF_*: 245.7 Hz), 164.6, 169.7. HRMS (*m*/*z*): [M+H]^+^: calculated: 434.1082, found: 434.1092.

##### 2-((3-(4-Fluorophenyl)-4-oxo-3,4-dihydroquinazolin-2-yl)thio)-*N*′-(pyridin-2-ylmethylene)acetohydrazide (**4q**)

C_22_H_16_FN_5_O_2_S, m.p.: 219–220 °C, yield: 66%. ^1^H-NMR (300 MHz, DMSO-*d*_6_): δ: 4.03 and 4.51 (2H, 2s, S-CH_2_), 7.41–7.51 (5H, m, Ar-CH), 7.58–7.62 (2H, m, Ar-CH), 7.78–7.95 (3H, m, Ar-CH), 8.08–8.30 (2H, m, Ar-CH), 8.62–8.64 (1H, m, Ar-CH), 11.88 and 12.02 (1H, 2s, NH). ^13^C-NMR: (75 MHz, DMSO-*d*_6_): δ: 34.5, 35.9, 117.0 (*J_CF_*: 22.8 Hz), 120.4, 124.8, 126.4, 126.5, 127.1, 132.4 (*J_CF_*: 9.2 Hz), 135.5, 137.3, 144.3, 147.5, 150.0, 153.4, 157.4, 161.2, 163,1 (*J_CF_*: 245.3 Hz), 164.4, 169.5. HRMS (*m*/*z*): [M+H]^+^: calculated: 434.1082, found: 434.1066.

##### 2-((3-(4-Fluorophenyl)-4-oxo-3,4-dihydroquinazolin-2-yl)thio)-*N*′-(thiophen-2-ylmethylene)acetohydrazide (**4r**)

C_21_H_15_FN_4_O_2_S_2_, m.p.: 249–250 °C, yield: 73%. ^1^H-NMR (300 MHz, DMSO-*d*_6_): δ: 3.98 and 4.41 (2H, 2s, S-CH_2_), 7.12–7.15 (1H, m, Ar-CH), 7.41–7.65 (8H, m, Ar-CH), 7.78–7.87 (1H, m, Ar-CH), 8.08 (1H, d, *J*: 6.93 Hz, Ar-CH), 8.23 and 8.47 (1H, 2s, Ar-CH), 11.68 (1H, s, NH). ^13^C-NMR: (75 MHz, DMSO-*d*_6_): δ: 34.7, 117.0 (*J_CF_*: 23.0 Hz), 120.0, 126.5, 127.1, 128.4, 129.0, 129.5, 131.0, 131.6, 132.3 (*J_CF_*: 9.2 Hz), 135.4, 139.0, 139.3, 142.3, 147.6, 157.5, 161.2, 163.1 (*J_CF_*: 245.6 Hz), 163.8, 168.9. HRMS (*m*/*z*): [M+H]^+^: calculated: 439.0693, found: 439.0691.

##### *N*′-((1*H*-Pyrrol-2-yl)methylene)-2-((3-(4-fluorophenyl)-4-oxo-3,4-dihydroquinazolin-2-yl)thio)acetohydrazide (**4s**)

C_21_H_16_FN_5_O_2_S, m.p.: 187–188 °C, yield: 78%. ^1^H-NMR (300 MHz, DMSO-*d*_6_): δ: 3.99 and 4.45 (2H, 2s, S-CH_2_), 6.14 (1H, s Ar-CH), 6.46–6.48 (1H, m, Ar-CH), 6.92 (1H, d, *J*: 14.94 Hz, Ar-CH), 7.43–7.63 (6H, m, Ar-CH), 7.78–7.90 (2H, m, Ar-CH), 8.08–8.10 (1H, s, Ar-CH), 11.36 (1H, 2s, NH), 11.46 (1H, 2s, NH). ^13^C-NMR: (75 MHz, DMSO-*d*_6_): δ: 34.8, 36.1, 109.7, 112.8, 113.9, 117.0 (*J_CF_*: 22.8 Hz), 120.0, 122.4, 123.0, 126.5, 126.6, 127.1, 127.2, 127.5, 132.3 (*J_CF_*: 6.9 Hz), 135.5, 147.6, 161.2, 163.1 (*J_CF_*: 247.5 Hz), 168.4. HRMS (*m*/*z*): [M+H]^+^: calculated: 422.1082, found: 422.1091.

##### 2-((3-(4-Fluorophenyl)-4-oxo-3,4-dihydroquinazolin-2-yl)thio)-*N*′-(furan-2-ylmethylene)acetohydrazide (**4t**)

C_21_H_15_FN_4_O_3_S, m.p.: 230–231 °C, yield: 76%. ^1^H-NMR (300 MHz, DMSO-*d*_6_): δ: 3.99 and 4.41 (2H, 2s, S-CH_2_), 6.62–6.65 (1H, m, Ar-CH), 6.91–6.93 (1H, m, Ar-CH), 7.42–7.51 (4H, m, Ar-CH), 7.56–7.62 (2H, m, Ar-CH), 7.78–7.88 (2H, m, Ar-CH), 7.95 and 8.15 (1H, 2s, Ar-CH), 8.08 (1H, d, *J*: 7.77 Hz, Ar-CH), 11.62 and 11.72 (1H, 2s, NH). ^13^C-NMR: (75 MHz, DMSO-*d*_6_): δ: 34.8, 35.9, 112.7, 114.1, 117.0 (*J_CF_*: 22.9 Hz), 120.0, 126.5, 127.1, 132.3 (*J_CF_*: 9.1 Hz), 134.1, 135.4, 137.0, 145.5, 147.6, 149.5, 157.5, 161.2, 163.1 (*J_CF_*: 243.0 Hz), 163.9, 169.0. HRMS (*m*/*z*): [M+H]^+^: calculated: 423.0922, found: 423.0917.

##### *N*′-((1*H*-Indol-3-yl)methylene)-2-((3-(4-fluorophenyl)-4-oxo-3,4-dihydroquinazolin-2-yl)thio)acetohydrazide (**4u**)

C_25_H_18_FN_5_O_2_S, m.p.: 291–292 °C, yield: 68%. ^1^H-NMR (300 MHz, DMSO-*d*_6_): δ: 4.02 and 4.61 (2H, 2s, S-CH_2_), 7.12–7.27 (2H, m, Ar-CH), 7.42–7.52 (4H, m, Ar-CH), 7.57–7.66 (3H, m, Ar-CH), 7.84–7.88 (2H, m, Ar-CH), 8.11 (1H, d, *J*: 7.56 Hz, Ar-CH), 8.24 (1H, s, Ar-CH), 8.26 and 8.42 (1H, 2s, Ar-CH), 11.39 and 11.43 (1H, 2s, NH), 11.61 (1H, s, NH). ^13^C-NMR: (75 MHz, DMSO-*d*_6_): δ: 36.0, 111.9, 112.5, 117.0 (*J_CF_*: 22.7 Hz), 120.0, 120.9, 122.1, 123.2, 124.5, 126.5, 127.1, 131.2, 132.4 (*J_CF_*: 8.9 Hz), 135.5, 137.6, 141.6, 147.7, 157.8, 161.3, 162.9, 163.1 (*J_CF_*: 245.3 Hz), 168.2. HRMS (*m*/*z*): [M+H]^+^: calculated: 472.1238, found: 472.1242.

##### 2-((3-(4-Fluorophenyl)-4-oxo-3,4-dihydroquinazolin-2-yl)thio)-*N*′-(naphthalen-1-ylmethylene)acetohydrazide (**4v**)

C_27_H_19_FN_4_O_2_S, m.p.: 257–258 °C, yield: 66%. ^1^H-NMR (300 MHz, DMSO-*d*_6_): δ: 4.07 and 4.58 (2H, 2s, S-CH_2_), 7.43–7.66 (9H, m, Ar-CH), 7.76–7.91 (2H, m, Ar-CH), 7.95–8.11 (3H, m, Ar-CH), 8.73–8.89 (2H, m, Ar-CH), 11.74 and 11.90 (1H, 2s, NH). ^13^C-NMR: (75 MHz, DMSO-*d*_6_): δ: 35.0, 35.9, 117.0 (*J_CF_*: 22.8 Hz), 120.0, 124.3, 124.8, 126.0, 126.5, 126.8, 127.1, 127.8, 128.0, 129.4, 129.8, 130.5, 130.9, 132.4 (*J_CF_*: 9.1 Hz), 134.0, 135.4, 143.9, 147.2, 147.6, 157.5, 161.2, 163.1 (*J_CF_*: 245.6 Hz), 164.0, 169.1. HRMS (*m*/*z*): [M+H]^+^: calculated: 483.1286, found: 483.1272.

##### 2-((3-(4-Fluorophenyl)-4-oxo-3,4-dihydroquinazolin-2-yl)thio)-*N*′-(naphthalen-2-ylmethylene)acetohydrazide (**4w**)

C_27_H_19_FN_4_O_2_S, m.p.: 268–269 °C, yield: 65%. ^1^H-NMR (300 MHz, DMSO-*d*_6_): δ: 4.05 and 4.55 (2H, 2s, S-CH_2_), 7.42–7.64 (8H, m, Ar-CH), 7.77–7.87 (1H, m, Ar-CH), 7.91–8.02 (4H, m, Ar-CH), 8.08–8.16 (2H, m, Ar-CH), 8.23 and 8.43 (1H, 2s, Ar-CH), 11.78 (1H, s, NH). ^13^C-NMR: (75 MHz, DMSO-*d*_6_): δ: 34.7, 36.0, 117.0 (*J_CF_*: 23.1 Hz), 120.0, 122.7, 123.1, 126.5, 127.1, 127.3, 127.6, 128.3, 128.8, 129.0, 129.2, 129.3, 132,4 (*J_CF_*: 9.4 Hz), 133.3, 134.1, 135.5, 143.9, 147.6, 157.5, 161.3, 163.1 (*J_CF_*: 243.5 Hz), 164.0, 169.3. HRMS (*m*/*z*): [M+H]^+^: calculated: 483.1286, found: 483.1280.

##### *N*′-(Cyclohexylmethylene)-2-((3-(4-fluorophenyl)-4-oxo-3,4-dihydroquinazolin-2-yl)thio)acetohydrazide (**4x**)

C_23_H_23_FN_4_O_2_S, m.p.: 228–229 °C, yield 69%. IR (ATR) υ_max_ (cm^−1^): 3078 (aromatic C-H), 2860 (aliphatic C-H), 1680, 1672 (CON-H), 1601, 1549, 1504, 1470 (C=C, C=N), 1192 (C-N, C-O), 966, 843, 770 (benzene ring out-of-plane deformation band). ^1^H-NMR (400 MHz, DMSO-*d*_6_): δ: 1.19–1.29 (5H, m, cyclohexyl CH), 1.61–1.71 (5H, m, cyclohexyl CH), 2.21 (1H, s, cyclohexyl CH), 3.90 and 4.31 (2H, 2s, S-CH_2_), 7.27 (1H, s, Ar-CH), 7.44–7.56 (6H, m, Ar-CH), 7.84 (1H, s, Ar-CH), 8.08 (1H, d, *J*: 5.80 Hz, Ar-CH), 11.17 and 11.27 (1H, s, NH). ^13^C-NMR: (100 MHz, DMSO-*d*_6_): δ: 25.4, 25.4, 26.0, 26.0, 30.0, 30.0, 34.7, 116.9 (*J_CF_*: 22.9 Hz), 120.0, 126.4, 127.1, 132.3 (*J_CF_*: 9.1 Hz), 135.4, 147.5, 151.7, 155.1, 157.5, 161.2, 163.1 (*J_CF_*: 245.3 Hz), 163.4, 168.7.

### 3.2. Biochemistry

#### 3.2.1. Cytotoxicity

The MTT assays were performed as in a previous study [48]. All final compounds were tested on a human lung carcinoma cell line (A549), a human breast adenocarcinoma cell line (MCF-7), and a healthy connective tissue cell line (L929) to determine their cytotoxic profiles. A549, MCF-7, and L929 cells were cultured in RPMI-1640 medium at 37 °C, 5% CO_2_, and 95% relative humidity. The cells were cultured in 96-well flat-bottomed plates at 37 °C for 24 h (2 × 10^4^ cells per well). Subsequently, all compounds were individually dissolved in DMSO and added to the culture wells at final concentrations of 1000, 500, 100, 50, 10, 5, and 1 μg/mL for the cancer cell lines (A549 and MCF-7) and 500, 250, 125, 62.5, 31.25, 15.63, 7.81, and 3.91 μg/mL for the non-cancerous cell line (L929). After a 24 h drug incubation at 37 °C, 20 µL of MTT solution (5 mg/mL MTT powder in PBS) was added to each well, and incubation was continued for an additional 4 h under the same conditions. At the end of the procedure, the resulting purple formazan crystals were dissolved in 100 µL of DMSO, and the absorbance was measured using an ELISA reader (OD at 570 nm). Cell viability was calculated as a percentage and compared to control cells. The IC_50_ values were calculated by repeating the experiment in three wells for each concentration [49].

#### 3.2.2. Apoptosis

Compounds **4c**, **4i**, **4m**, and **4u**, which exhibited the strongest cytotoxic effects in this series, were analyzed using the Annexin V-FITC apoptosis detection kit (BD Pharmingen, San Jose, CA, USA) on a BD FACS Aria flow cytometry to determine the rates of apoptosis and necrosis they induced. The compounds were treated with A549 cells at IC_50_ doses, and the rates of early and late apoptosis, necrosis, and live cells were determined as percentages. After incubating A549 cells with compounds **4c**, **4i**, **4m**, and **4u**, as well as cisplatin and gefitinib at IC_50_ concentrations for 24 h, the externalization of phosphatidylserine, indicative of early apoptosis, was measured using the FITC Annexin V apoptotic cell detection kit (BD Pharmingen, San Jose, CA, USA). The data were analyzed using FACS Diva version 6.1.1 software (BD Biosciences, San Jose, CA, USA) on a BD FACS Aria flow cytometry [51].

#### 3.2.3. Caspase-3 Activity Analysis

For the caspase-3 activation assay, A549 lung cancer cells and MCF-7 breast cancer cells were seeded in 6-well plates at a density of 2 × 10^5^ cells/mL per well and incubated for 24 h. Following incubation, cisplatin, used as the reference drug, and the synthesized compounds were added to the wells at their respective IC_50_ concentrations and incubated for an additional 24 h. The cells were then washed with phosphate-buffered saline (PBS), detached using trypsin, and transferred to appropriate tubes for flow cytometric analysis. The samples were centrifuged at 1200 rpm for 4 min, after which the supernatant was discarded. The resulting cell pellets were washed with 1 mL of PBS.

Caspase-3 activity was subsequently assessed according to the manufacturer’s instructions (BD Biosciences Pharmingen, Singapore). Briefly, the cells were washed with cold PBS and incubated with 0.5 mL of permeabilizing lysis solution for 30 min at room temperature in the dark. The cell pellets were then washed twice with 0.5 mL of permeabilizing wash buffer. The cells were resuspended in 100 μL of permeabilizing wash buffer, followed by the addition of 10 μL of the caspase-3 antibody. The samples were incubated for 20 min at room temperature in the dark. At least 10,000 cells were analyzed for each sample using a Beckman Coulter CytoFLEX flow cytometer (Brea, CA, Indianapolis, IN, USA) and CytExpert software version 2.7 [51,52].

#### 3.2.4. Cell Cycle Analysis

The cell cycle distribution of the A549 cell line was analyzed by flow cytometry using compounds **4c**, **4i**, **4m**, and **4u**, which exhibited the strongest cytotoxic effects in this series, in accordance with the manufacturer’s (BD Biosciences, San Jose, CA, USA) instructions. Following a 24 h incubation period with the compounds, the cell cycle analysis measurement methodology was applied for A549 cells in accordance with the manufacturer’s instructions (BD Biosciences, Singapore). The data were analyzed using the BD Biosciences ModFit LT Version 6.0 software on a flow cytometry. This method was applied as in our previous studies [53].

#### 3.2.5. EGFR Inhibition

EGFR, a member of the receptor tyrosine kinase family, has been associated with the development and progression of various cancers through overexpression or hyperactivation. To evaluate EGFR inhibition, compounds **4i**, **4m**, and **4u**, which exhibited the highest cytotoxic activity in this series, were selected for further analysis using the colorimetric Human EGFR Intracellular ELISA Kit (Abcam, Cambridge, MA, USA; Cat. No. ab126419) in A549 cells. The assay was performed for 24 h in accordance with the manufacturer’s instructions. Gefitinib, a potent EGFR inhibitor, was used as positive control. The selected compounds were tested at IC_50_, IC_50_/100, and IC_50_/1000 concentrations, with three independent replicates for each condition. The results are expressed as mean ± standard deviation (SD) [44].

#### 3.2.6. Statistical Analyses

Statistical analyses for calculating results in biological tests were performed using the Social Sciences Statistical Package (SPSS) for Windows 23.0. Data were presented as mean ± standard deviation, and comparisons were made using one-way ANOVA for normally distributed continuous variables. Tukey’s test was used for post hoc analyses of group differences, and *p* < 0.05 was considered statistically significant. Apoptotic results (Annexin V-FITC and caspase-3, Mitochondrial Membrane Polarization) were evaluated using flow cytometry with FACS Diva Version 6.1.1 software, and apoptotic cells were determined as a percentage of cells.

### 3.3. In Silico Methods

Molecular modeling studies were performed to determine the possible binding modes of the active compounds within the target protein regions. As in the previous studies, same methodologies are applied [52,54]. In this context, the X-ray crystal structures of the target proteins used in the molecular docking studies were obtained from the Protein Data Bank, namely active caspase-3 with PDB ID: 4QTX and inactive EGFR with PDB ID: 4HJO. Molecular docking studies were carried out using the Schrödinger Maestro interface. The protein crystal structures were prepared using the Protein Preparation Wizard protocol of Schrödinger Suite 2020; missing atoms were completed, protonation states were assigned, and the necessary energy minimization procedures were performed. The compounds were prepared using the LigPrep module to assign appropriate protonation states and atom types. The grid files used for docking studies were generated using the Glide module, and molecular docking analyses were performed in standard precision (SP) mode [55,56,57].

For each ligand–protein complex, a 100 ns molecular dynamics simulation was performed. The simulations were conducted using the Desmond application included in the Schrödinger Suite. The transferable intermolecular potential three-point (TIP3P) water model and the OPLS3e force field were used in the simulations [58]. The system was neutralized using Na^+^ and Cl^−^ ions, and 150 mM NaCl was added to mimic physiological ionic conditions. After completion of the system setup, molecular dynamics simulations were performed. The radius of gyration (Rg), root mean square fluctuation (RMSF), and root mean square deviation (RMSD) values were calculated using the Desmond application to evaluate the simulation results [58]. In addition, plots related to binding modes and interaction durations were obtained and interpreted to assess the continuity of ligand–protein interactions and binding stability.

## 4. Conclusions

In this study, twenty-four derivatives of 2-[[4-oxo-3-(substituted phenyl)-3,4-dihydro-(substituted quinazolin-2-yl)]thio]-*N*′-(aryl/heteroaryl methylene)acetohydrazide (**4a**–**4x**) were synthesized, and their anticancer activities were evaluated on the A549 and MCF-7 cancer cell lines and the L929 healthy cell line. It was determined that compounds **4c**, **4i**, **4m**, and **4u** had IC_50_ values between 44.75 and 78.13 µM on A549 cells, while compounds **4i**, **4l**, **4m**, and **4u** had IC_50_ values between 14.43 and 29.39 µM on MCF-7 cells, selectively. Compounds **4m** (53.37%) and **4u** (41.41%), exhibited significant apoptosis (early + late apoptotic cells) induction in A549 cells with higher potency than standard drugs, while compound **4l** showed an effect of 34.81% in MCF-7 cells. Compounds **4m** (13.06%) and **4u** (14.47%) provoked caspase-3 activation on A549, and compound **4i** surpassed (24.50%) the caspase-3 activation potential of cisplatin on MCF-7. In addition, other compounds, **4l**, **4m**, and **4u**, also caused higher activation in caspase-3 activation exhibited by gefitinib on MCF-7 cells. Most of the compounds induced apoptosis by causing pre-G and S-phase arrest on both cell lines. Compounds **4m** and **4u** exhibited EGFR inhibition with IC_50_ values of 4.80 µM and 5.40 µM; while not as effective as gefitinib, it has a good level of efficacy. In conclusion, the combined biological activity assays, molecular docking, and molecular dynamics simulation studies suggest that the 4-quinazolinone–acetyl hydrazone scaffold bearing a thioether linker represents a promising structural framework for the development of potential anticancer agents. When the general caspase results of the compounds were examined, compounds **4l**, **4m**, and **4u** were studied in molecular docking, and the contribution of π–π interaction with Phe256 interaction to activity was evaluated in compounds containing bulky groups such as indole and naphthalene. It was determined that this situation is similar in molecular docking studies on EGFR. **4m**, 2-((3-(4-chlorophenyl)-4-oxo-3,4-dihydroquinazolin-2-yl)thio)-*N*′-(naphthalen-1-ylmethylene)acetohydrazide and **4u**, *N*′-((1*H*-indol-3-yl)methylene)-2-((3-(4-fluorophenyl)-4-oxo-3,4-dihydroquinazolin-2-yl)thio)acetohydrazide were found to fit well in EGFR binding pockets. Additionally, the acetyl moiety appears to act as an important pharmacophoric group, while the trisubstituted quinazolinone core may contribute favorably to caspase-3 activation.

## Figures and Tables

**Table 1:** IC_50_ values (µM) of the compounds on A549, MCF-7 and L929 cells.

	A549	MCF-7	L929
**4a**	155.09 ± 16.86	513.65 ± 93.44	>500
**4b**	>500	284.69 ± 14.58	>500
**4c**	78.13 ± 13.35	112.10 ± 14.64	168.35 ± 8.58
**4d**	>500	>500	>500
**4e**	247.22 ± 22.35	230.09 ± 31.72	178.79 ± 17.51
**4f**	>500	>500	>500
**4g**	>500	>500	>500
**4h**	89.62 ± 4.19	63.44 ± 5.48	78.18 ± 7.30
**4i**	62.13 ± 4.33	27.59 ± 4.75	220.09 ± 2.86
**4j**	>500	>500	>500
**4k**	>500	>500	>500
**4l**	124.37 ± 6.83	29.39 ± 2.03	132.94 ± 2.79
**4m**	44.75 ± 0.50	14.43 ± 3.28	>500
**4n**	>500	>500	>500
**4o**	>500	>500	>500
**4p**	>500	>500	>500
**4q**	>500	>500	>500
**4r**	360.69 ± 105.37	498.97 ± 118.17	>500
**4s**	>500	>500	>500
**4t**	>500	>500	>500
**4u**	58.69 ± 4.41	26.15 ± 3.15	132.85 ± 13.53
**4v**	>500	>500	>500
**4w**	>500	>500	>500
**4x**	196.23 ± 29.65	128.05 ± 9.84	>500
**Cisplatin**	14.00 ± 2.64	139.55 + 5.12	320.18 ± 24.48
**Gefitinib**	35.58 ± 0.38	465.32 ± 87.30	157.23 ± 9.66

**Table 2:** Percentage analysis of Annexin V-FITC/propidium iodide flow cytometry in A549 cells treated with compounds **4i**, **4m**, **4u**, cisplatin and gefitinib.

Groups	Early Apoptotic Cells	Late Apoptotic Cells	Viable Cells	Necrotic Cells
Control	7.54	1.48	90.85	0.13
**4i**	17.65	7.20	74.33	0.82
**4m**	48.26	5.11	45.82	0.81
**4u**	35.27	6.14	57.93	0.66
Cisplatin	16.96	4.73	77.90	0.40
Gefitinib	13.45	8.49	76.82	1.24

**Table 3:** Percentage analysis of Annexin V-FITC/propidium iodide flow cytometry in MCF-7 cells treated with compound **4i**, **4l**, **4m**, **4u**, cisplatin and gefitinib.

Groups	Early Apoptotic Cells	Late Apoptotic Cells	Viable Cells	Necrotic Cells
Control	4.31	2.32	92.39	0.98
**4i**	18.92	9.82	69.78	1.48
**4l**	27.28	7.53	63.57	1.62
**4m**	17.29	8.46	73.01	1.24
**4u**	16.29	8.06	74.24	1.41
Cisplatin	39.80	17.42	39.70	3.08
Gefitinib	30.60	1.74	66.07	1.60

**Table 4:** Percentage flow cytometry quadrants of active caspase-3 activity in A549 cells treated with compounds **4i**, **4m**, **4u**, cisplatin and gefitinib.

Groups	P2: Caspase-3-Negative Cells	P3: Caspase-3-Positive Cells
Control	99.28	0.53
**4i**	92.36	6.32
**4m**	85.75	13.06
**4u**	81.78	14.47
Cisplatin	84.51	14.66
Gefitinib	92.25	6.81

**Table 5:** Percentage flow cytometry quadrants of active caspase-3 activity in MCF-7 cells treated with compounds **4i**, **4l**, **4m**, **4u**, cisplatin and gefitinib.

Groups	P2: Caspase-3-Negative Cells	P3: Caspase-3-Positive Cells
Control	96.05	3.30
**4i**	73.07	24.50
**4l**	75.69	20.89
**4m**	78.78	19.24
**4u**	74.33	22.58
Cisplatin	74.96	22.70
Gefitinib	83.21	15.07

**Table 6:** Cell cycle percentages of compounds on A549 cells.

Compounds	Sub G0-Pre G	% G0/G1 Phase	% S Phase	% G2/M Phase
Control	4.52	79.35	5.82	9.97
**4i**	22.52	59.04	8.49	9.59
**4m**	50.60	31.90	8.97	8.32
**4u**	55.55	29.85	7.69	6.56
Cisplatin	17.91	58.67	10.90	12.16
Gefitinib	9.05	75.99	4.66	10.15

**Table 7:** Cell cycle percentages of compounds on MCF-7 cells.

Compounds	Sub G0-Pre G	% G0/G1 Phase	% S Phase	% G2/M Phase
Control	6.30	55.76	14.11	23.73
**4i**	22.73	52.87	5.47	18.96
**4l**	39.28	44.41	4.37	11.91
**4m**	31.86	43.46	7.40	17.39
**4u**	30.62	41.24	4.99	23.09
Cisplatin	41.19	44.89	5.42	8.43
Gefitinib	90.27	7.61	1.47	0.69

**Table 8:** IC_50_ values (µM) on EGFR.

Compounds	IC_50_
**4i**	9.12 ± 1.40
**4m**	4.80 ± 0.40
**4u**	5.40 ± 1.79
Gefitinib	1.86 ± 0.43

## Data Availability

The Supplementary Document is accessible along with this article published in the journal.

## Supplementary Materials

The following supporting information can be downloaded at: https://www.mdpi.com/article/10.3390/molecules31183163/s1.
