# Supplementary material for: Synthesis of Quinazoline Derivatives and Mechanistic Approaches in Lung and Breast Cancers
Source: Molecules. 2026 Sep 8;31(18):3163. doi: 10.3390/molecules31183163 (PMC13608900; doi:10.3390/molecules31183163)
Supplement: Supplementary file 1 [file molecules-31-03163-s001.zip › molecules-4479937-supplementary.pdf]

Synthesis of Quinazoline Derivatives and Mechanistic Approaches in Lung and Breast Cancers

Aybüke Züleyha Kaya [1,2], Beyzanur Tutuş [2,3], Şevval Karaca Arpa [4,5], Asaf Evrim Evren [1], Gülşen Akalin Çiftçi [5], Halide Edip Temel [5] and Leyla Yurttaş [1,*]

[1] Department of Pharmaceutical Chemistry, Faculty of Pharmacy, Anadolu University, Eskişehir 26470, Türkiye

[2] Department of Pharmaceutical Chemistry, Graduate School, Anadolu University, Eskişehir 26470, Türkiye

[3] Kırıkhan Vocational School, Hatay Mustafa Kemal University, Hatay 31440, Türkiye

[4] Department of Biochemistry, Graduate School, Anadolu University, Eskişehir 26470, Türkiye

[5] Department of Biochemistry, Faculty of Pharmacy, Anadolu University, Eskişehir 26470, Türkiye

* Correspondence: lyurttas@anadolu.edu.tr

Data File: C:\LabSolutions\Data\Analiz\Asaf\AY-PK-1_493.lcd

Elmt	Val.	Min	Max	Elmt	Val.	Min	Max	Elmt	Val.	Min	Max	Elmt	Val.	Min	Max	Use Adduct
H	1	10	33	O	2	0	5	S	2	1	2	Ru	2	0	0	H
C	4	5	32	F	1	1	1	Cl	1	0	0	Pd	2	0	0	Na
N	3	0	5	P	3	0	0	Br	1	0	0	I	3	0	0	

Error Margin (ppm):	5	DBE Range:	0.0 - 30.0	Electron Ions:	both
HC Ratio:	unlimited	Apply N Rule:	no	Use MSn Info:	yes
Max Isotopes:	3	Isotope RI (%):	1.00	Isotope Res:	9000
MSn Iso RI (%):	10.00	MSn Logic Mode:	AND	Max Results:	50

Event#: 1 MS(E+) Ret. Time : 3.813 Scan# : 573

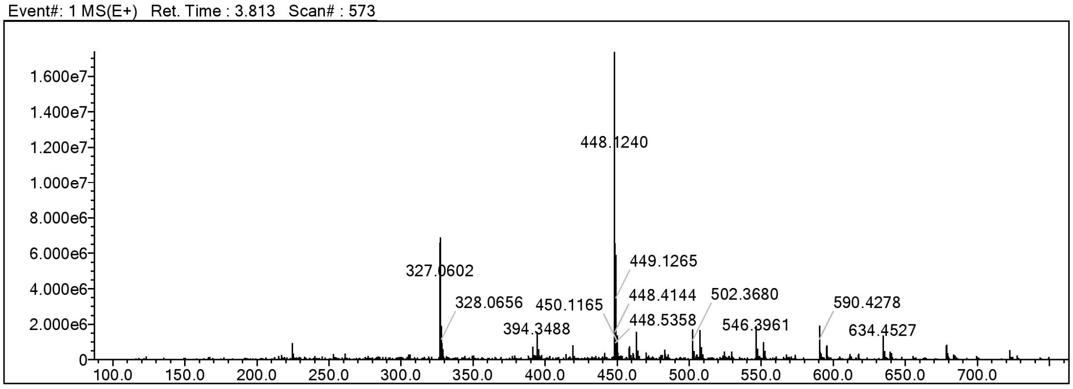

Measured region for 448.1240 m/z

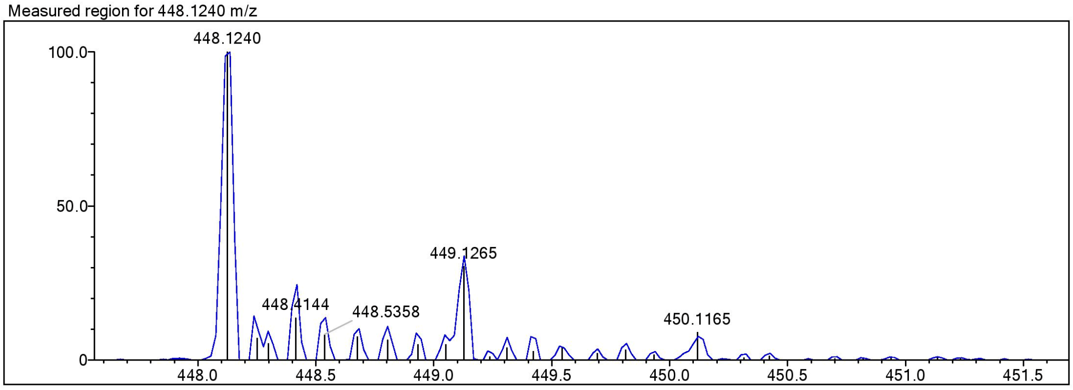

C23 H18 N5 O2 F S [M+H]+ : Predicted region for 448.1238 m/z

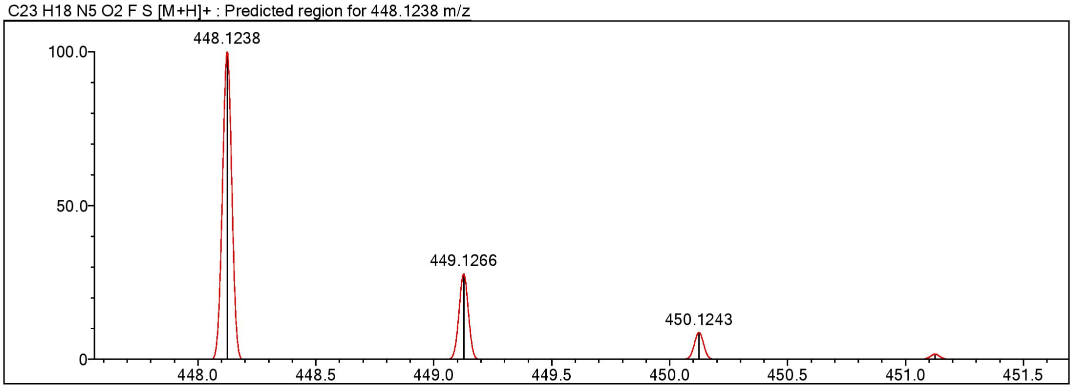

Rank	Score	Formula (M)	Ion	Meas. m/z	Pred. m/z	Df. (mDa)	Df. (ppm)	Iso	DBE
3	72.52	C23 H18 N5 O2 F S	[M+H]+	448.1240	448.1238	0.2	0.45	72.52	17.0

Figure S1. HRMS spectrum of compound **4a**

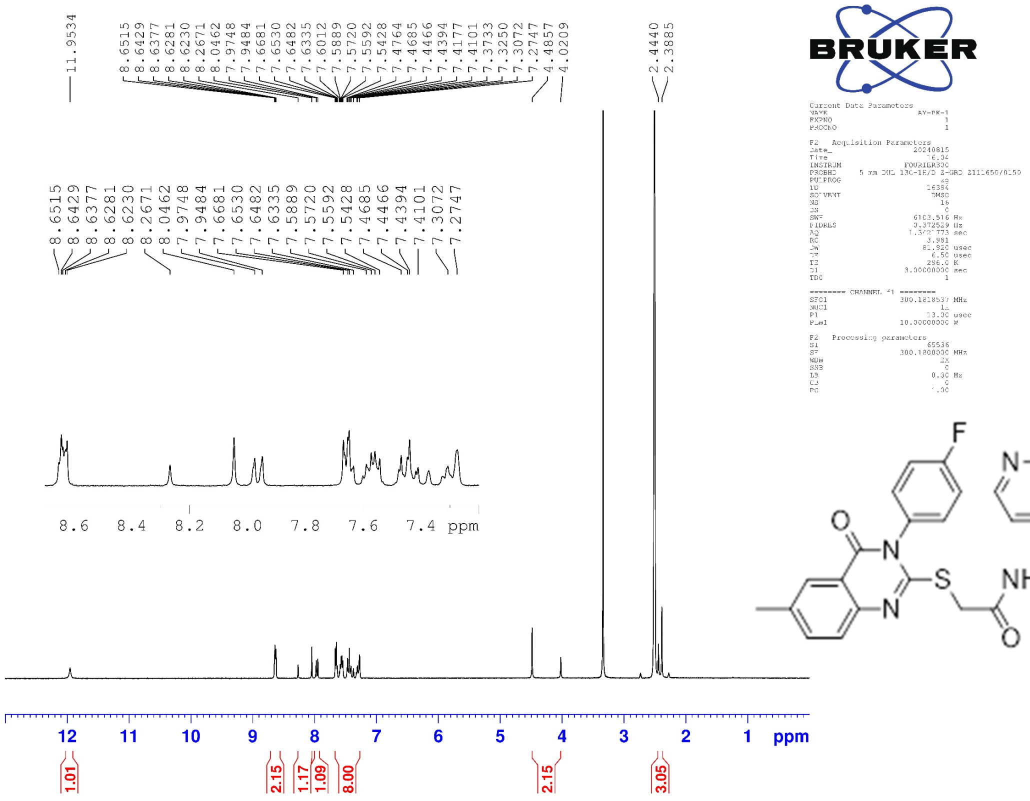

Figure S2. ^{1}H-NMR spectrum of compound **4a**

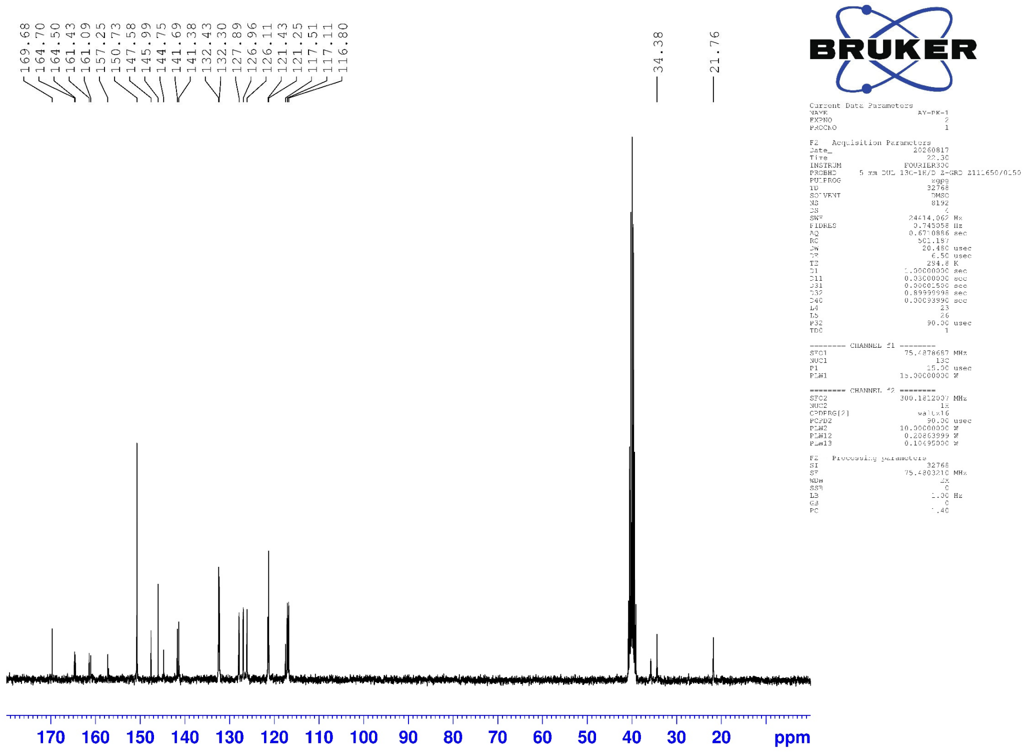

Figure S3. ^{13}C-NMR spectrum of compound **4a**

Data File: C:\LabSolutions\Data\Analiz\Asaf\AY-PK-2_494.lcd

Elmt	Val.	Min	Max	Elmt	Val.	Min	Max	Elmt	Val.	Min	Max	Elmt	Val.	Min	Max	Use Adduct
H	1	10	33	O	2	0	5	S	2	1	2	Ru	2	0	0	H
C	4	5	32	F	1	1	1	Cl	1	0	0	Pd	2	0	0	Na
N	3	0	5	P	3	0	0	Br	1	0	0	I	3	0	0	

Error Margin (ppm):	5	DBE Range:	0.0 - 30.0	Electron Ions:	both
HC Ratio:	unlimited	Apply N Rule:	no	Use MSn Info:	yes
Max Isotopes:	3	Isotope RI (%):	1.00	Isotope Res:	9000
MSn Iso RI (%):	10.00	MSn Logic Mode:	AND	Max Results:	50

Event#: 1 MS(E+) Ret. Time : 4.773 Scan# : 717

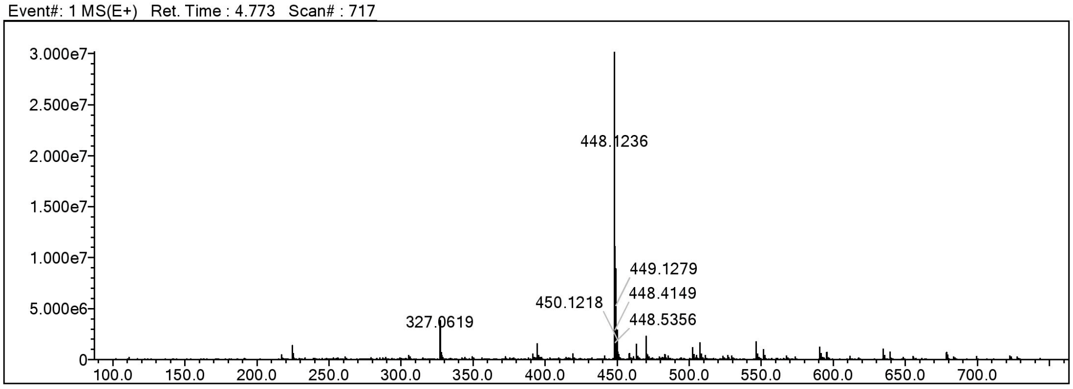

Measured region for 448.1236 m/z

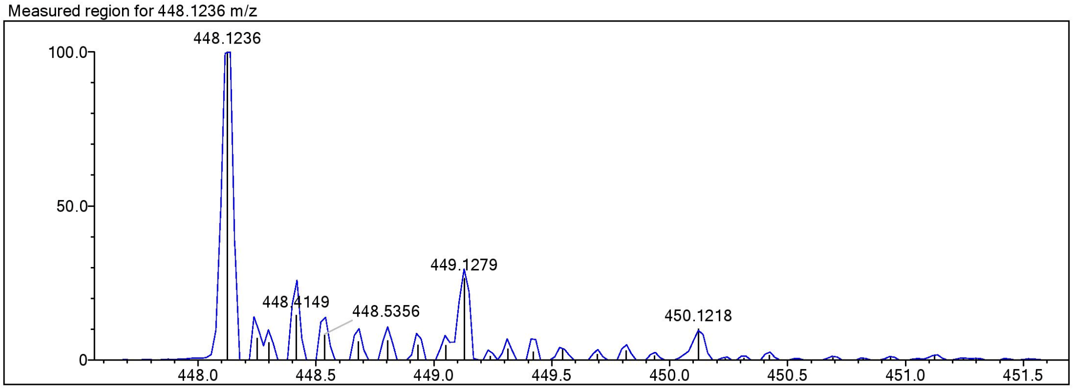

C23 H18 N5 O2 F S [M+H]+ : Predicted region for 448.1238 m/z

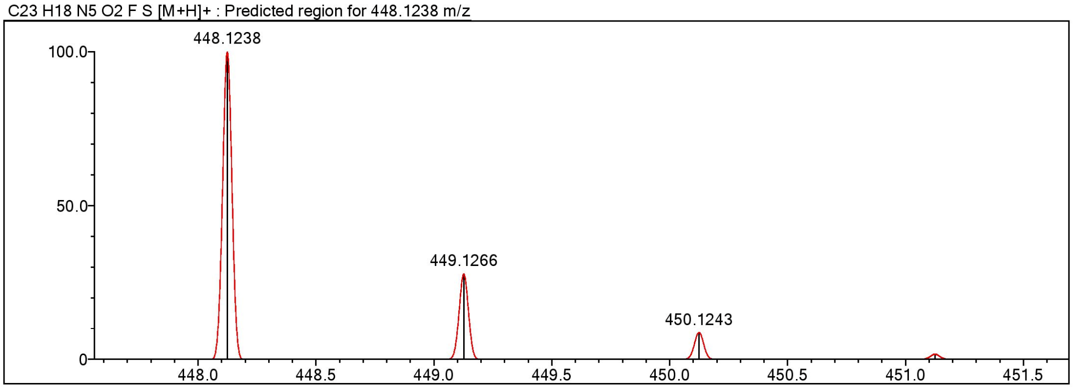

Rank	Score	Formula (M)	Ion	Meas. m/z	Pred. m/z	Df. (mDa)	Df. (ppm)	Iso	DBE
1	91.57	C23 H18 N5 O2 F S	[M+H]+	448.1236	448.1238	-0.2	-0.45	91.57	17.0

Figure S4. HRMS spectrum of compound **4b**

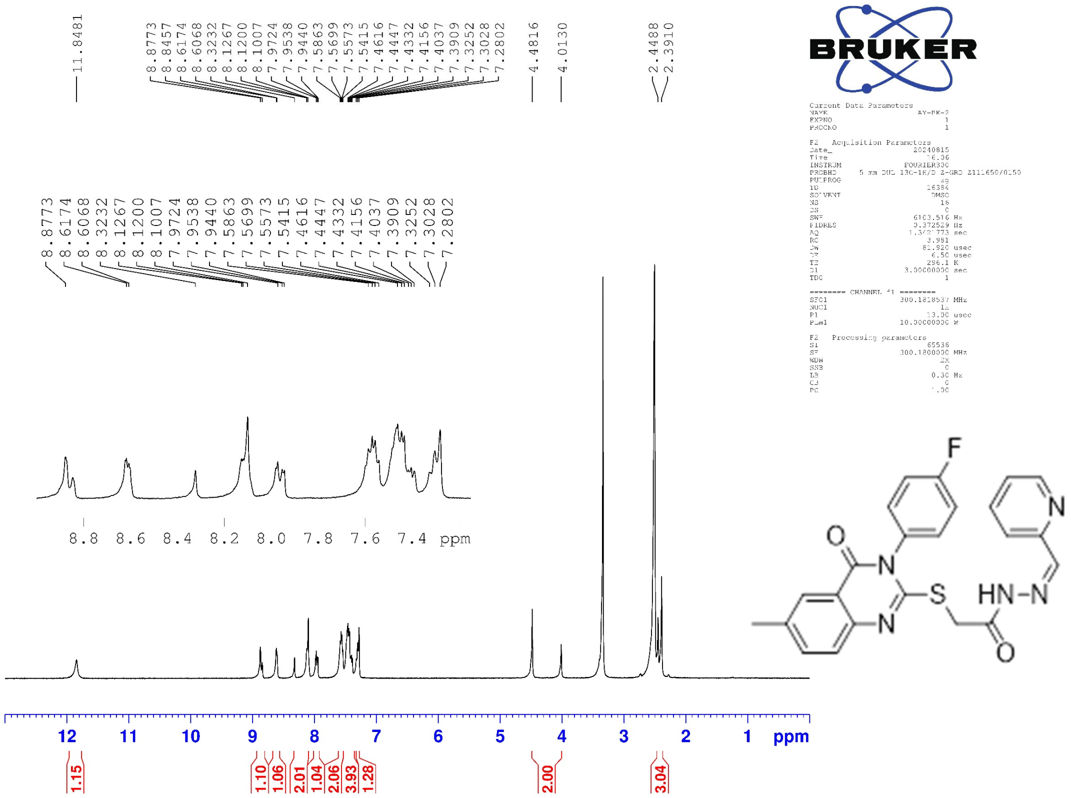

Figure S5. ^{1}H-NMR spectrum of compound **4b**

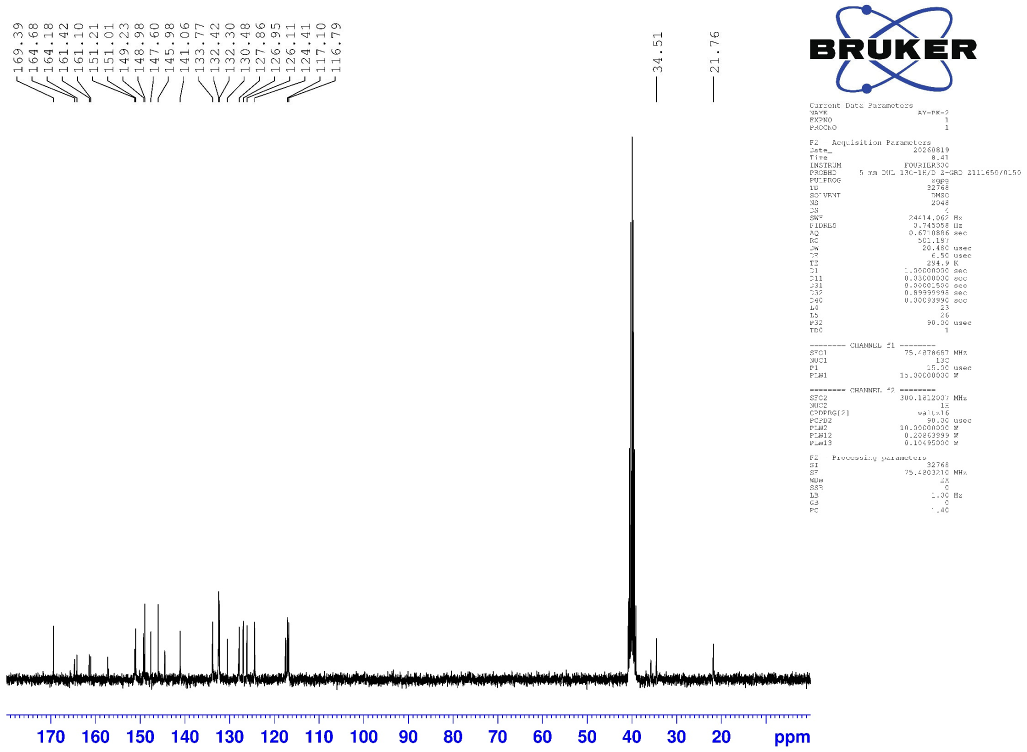

Figure S6. ^{13}C-NMR spectrum of compound **4b**

Data File: C:\LabSolutions\Data\Analiz\Asaf\AY-PK-3_495.lcd

Elmt	Val.	Min	Max	Elmt	Val.	Min	Max	Elmt	Val.	Min	Max	Elmt	Val.	Min	Max	Use Adduct
H	1	10	33	O	2	0	5	S	2	1	2	Ru	2	0	0	H
C	4	5	32	F	1	1	1	Cl	1	0	0	Pd	2	0	0	Na
N	3	0	5	P	3	0	0	Br	1	0	0	I	3	0	0	

Error Margin (ppm):	5	DBE Range:	0.0 - 30.0	Electron Ions:	both
HC Ratio:	unlimited	Apply N Rule:	no	Use MSn Info:	yes
Max Isotopes:	3	Isotope RI (%):	1.00	Isotope Res:	9000
MSn Iso RI (%):	10.00	MSn Logic Mode:	AND	Max Results:	50

Event#: 1 MS(E+) Ret. Time : 7.293 Scan# : 1095

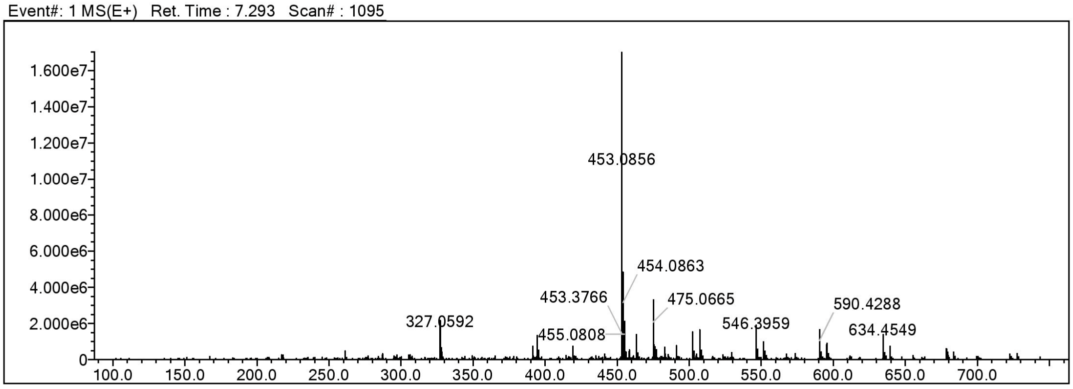

Measured region for 453.0856 m/z

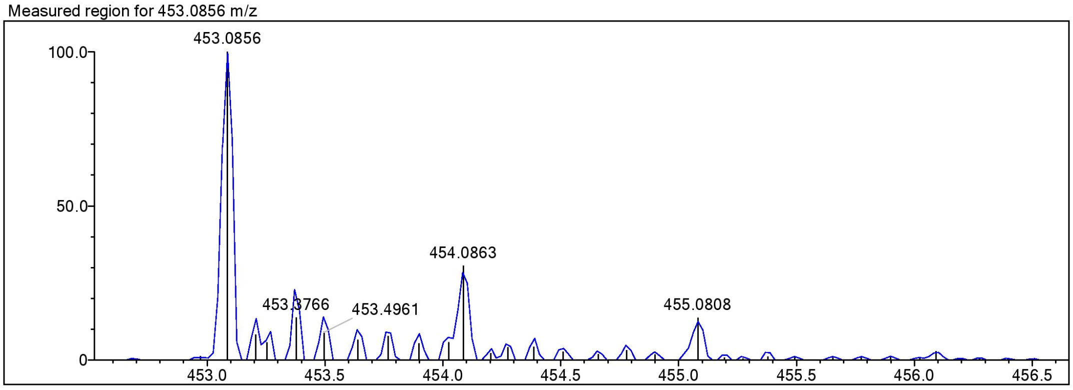

C22 H17 N4 O2 F S2 [M+H]+ : Predicted region for 453.0850 m/z

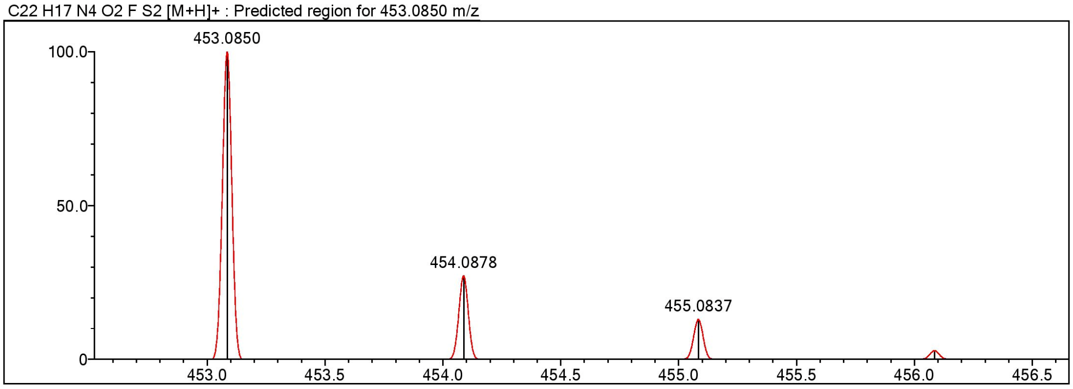

Rank	Score	Formula (M)	Ion	Meas. m/z	Pred. m/z	Df. (mDa)	Df. (ppm)	Iso	DBE
2	81.31	C22 H17 N4 O2 F S2	[M+H]+	453.0856	453.0850	0.6	1.32	81.96	16.0

Figure S7. HRMS spectrum of compound **4c**

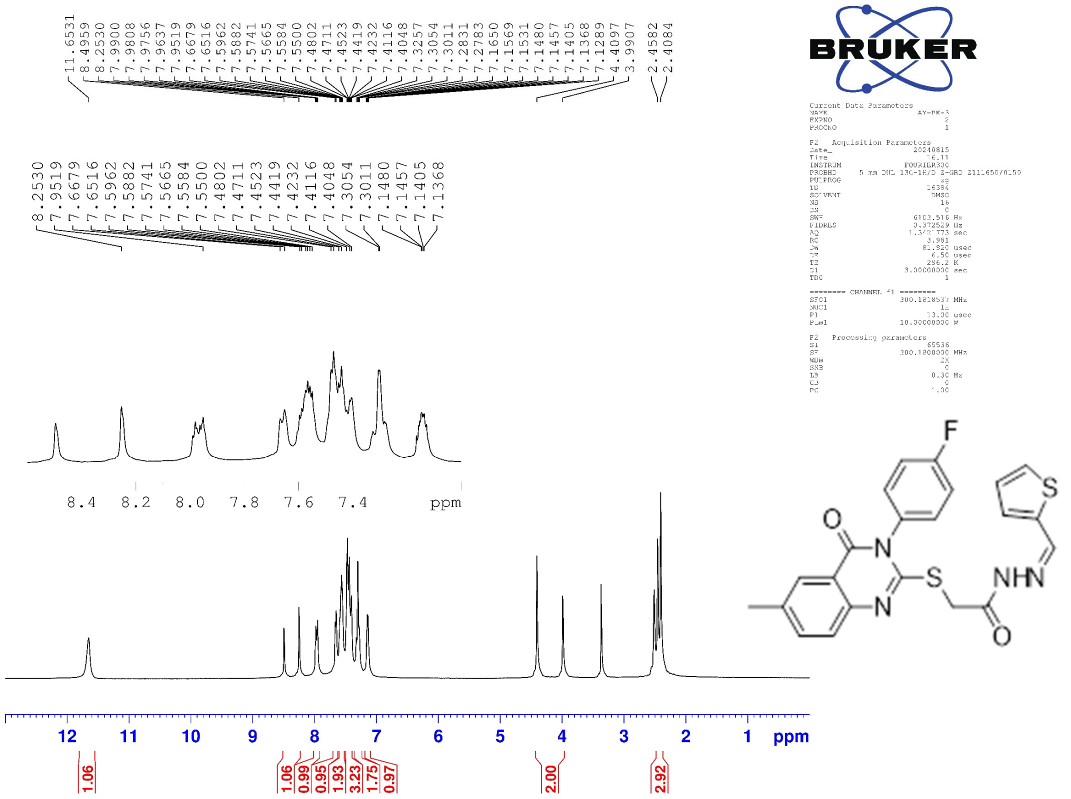

Figure S8. ^{1}H-NMR spectrum of compound **4c**

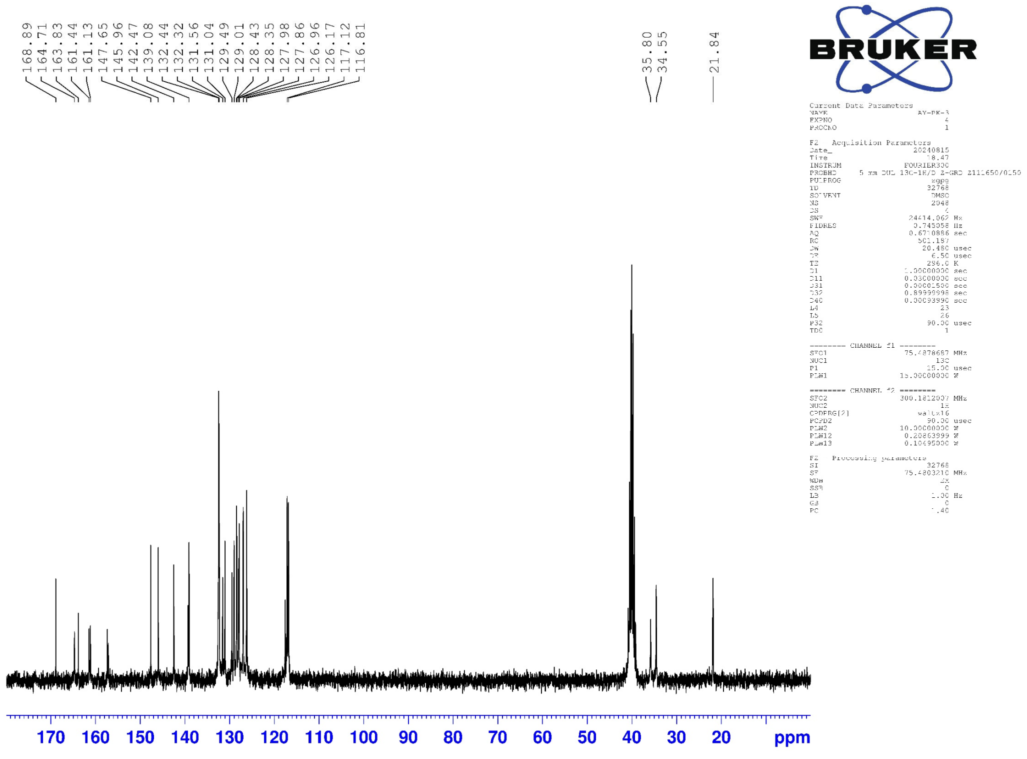

Figure S9. ^{13}C-NMR spectrum of compound **4c**

Data File: C:\LabSolutions\Data\Analiz\Asaf\AY-PK-4_496.lcd

Elmt	Val.	Min	Max	Elmt	Val.	Min	Max	Elmt	Val.	Min	Max	Elmt	Val.	Min	Max	Use Adduct
H	1	10	33	O	2	0	5	S	2	1	2	Ru	2	0	0	H
C	4	5	32	F	1	1	1	Cl	1	0	0	Pd	2	0	0	Na
N	3	0	5	P	3	0	0	Br	1	0	0	I	3	0	0	

Error Margin (ppm):	5	DBE Range:	0.0 - 30.0	Electron Ions:	both
HC Ratio:	unlimited	Apply N Rule:	no	Use MSn Info:	yes
Max Isotopes:	3	Isotope RI (%):	1.00	Isotope Res:	9000
MSn Iso RI (%):	10.00	MSn Logic Mode:	AND	Max Results:	50

Event#: 1 MS(E+) Ret. Time : 6.080 Scan# : 913

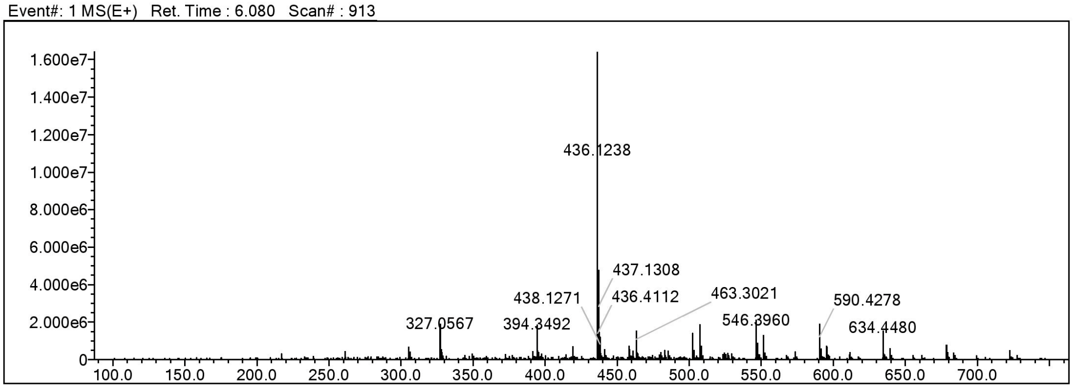

Measured region for 436.1238 m/z

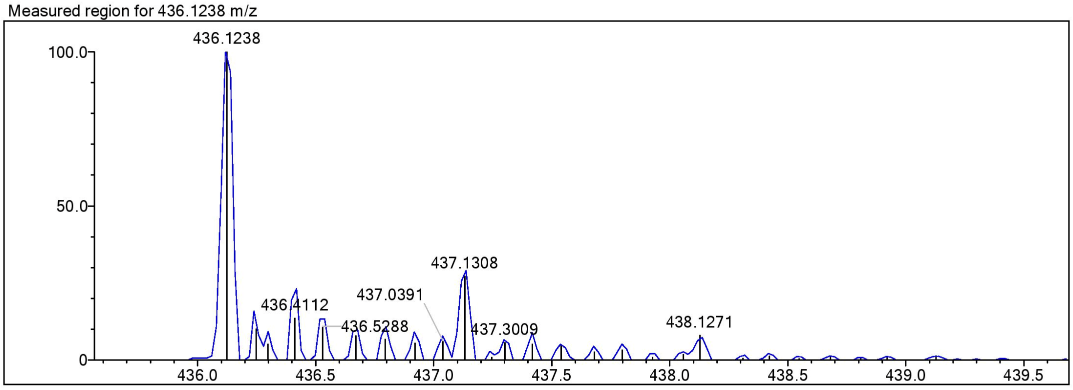

C22 H18 N5 O2 F S [M+H]+ : Predicted region for 436.1238 m/z

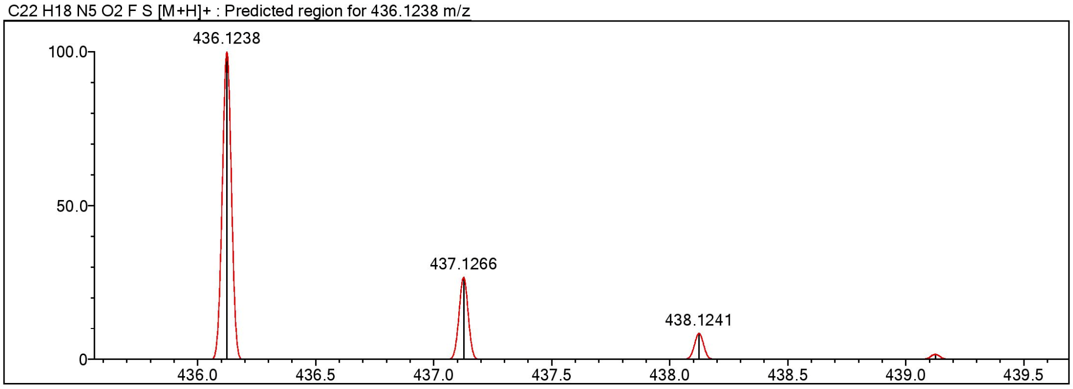

Rank	Score	Formula (M)	Ion	Meas. m/z	Pred. m/z	Df. (mDa)	Df. (ppm)	Iso	DBE
3	84.72	C22 H18 N5 O2 F S	[M+H]+	436.1238	436.1238	-0.0	0.00	84.72	16.0

Figure S10. HRMS spectrum of compound **4d**

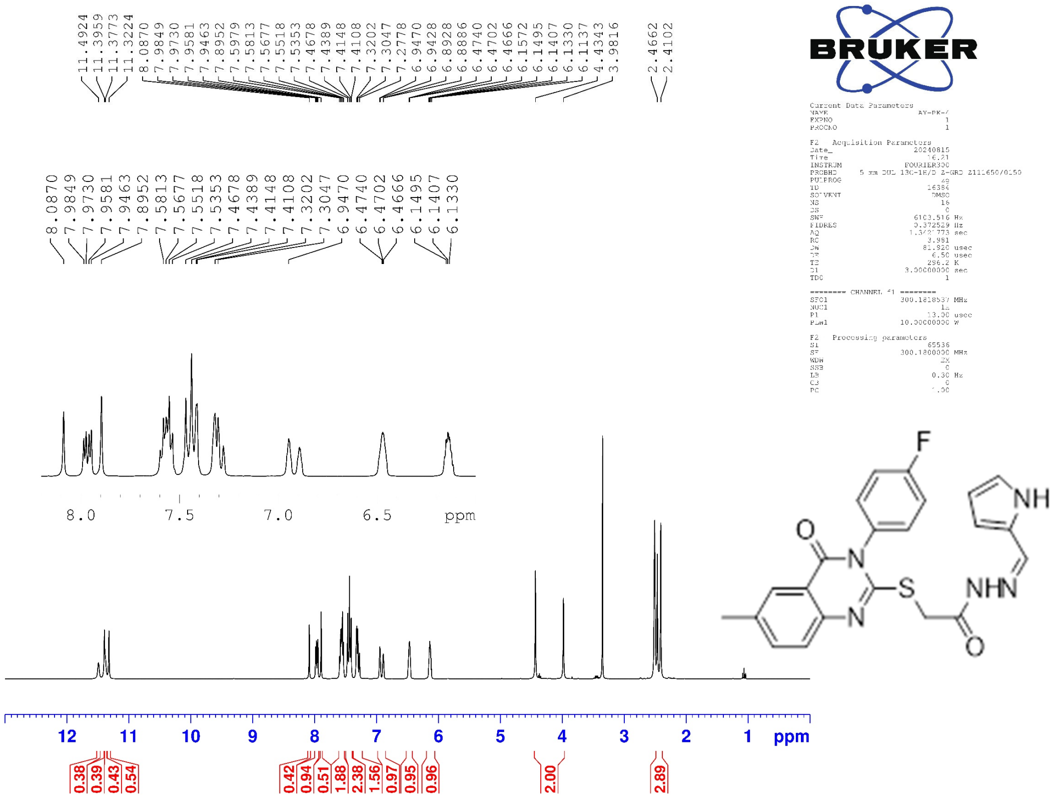

Figure S11. ^{1}H-NMR spectrum of compound **4d**

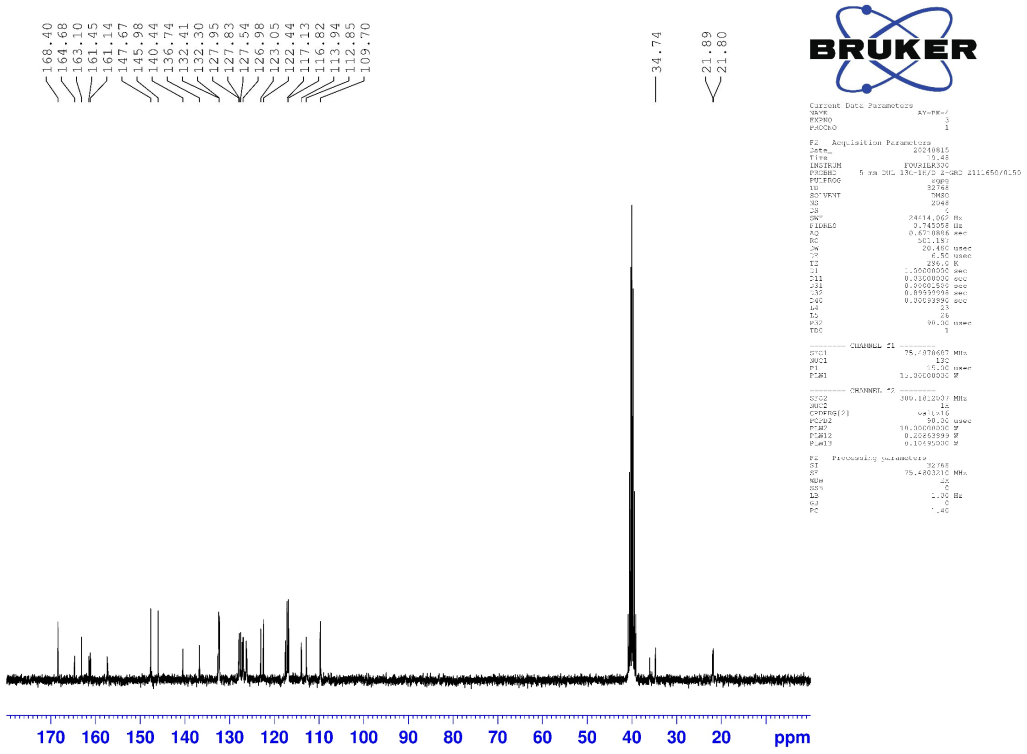

Figure S12. ^{13}C-NMR spectrum of compound **4d**

Data File: C:\LabSolutions\Data\Analiz\Asaf\AY-PK-5_497.lcd

Elmt	Val.	Min	Max	Elmt	Val.	Min	Max	Elmt	Val.	Min	Max	Elmt	Val.	Min	Max	Use Adduct
H	1	10	33	O	2	0	5	S	2	1	2	Ru	2	0	0	H
C	4	5	32	F	1	0	0	Cl	1	0	0	Pd	2	0	0	Na
N	3	0	5	P	3	0	0	Br	1	0	0	I	3	0	0	

Error Margin (ppm):	5	DBE Range:	0.0 - 30.0	Electron Ions:	both
HC Ratio:	unlimited	Apply N Rule:	no	Use MSn Info:	yes
Max Isotopes:	3	Isotope RI (%):	1.00	Isotope Res:	9000
MSn Iso RI (%):	10.00	MSn Logic Mode:	AND	Max Results:	50

Event#: 1 MS(E+) Ret. Time : 6.080 -> 6.613 - 0.640 -> 4.608 Scan# : 913 -> 993 - 97 -> 693

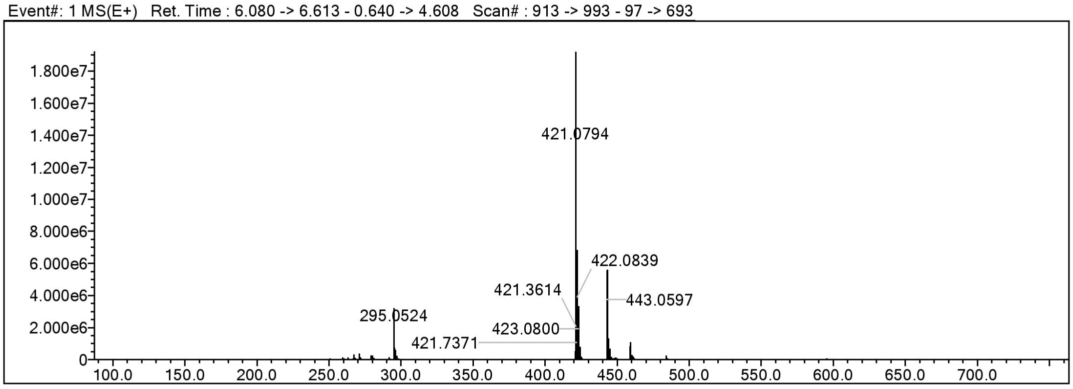

Measured region for 421.0794 m/z

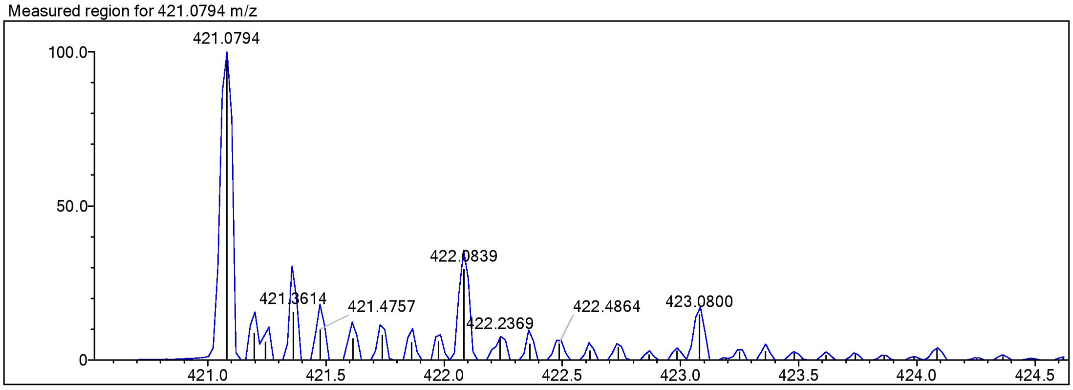

C21 H16 N4 O2 S2 [M+H]+ : Predicted region for 421.0787 m/z

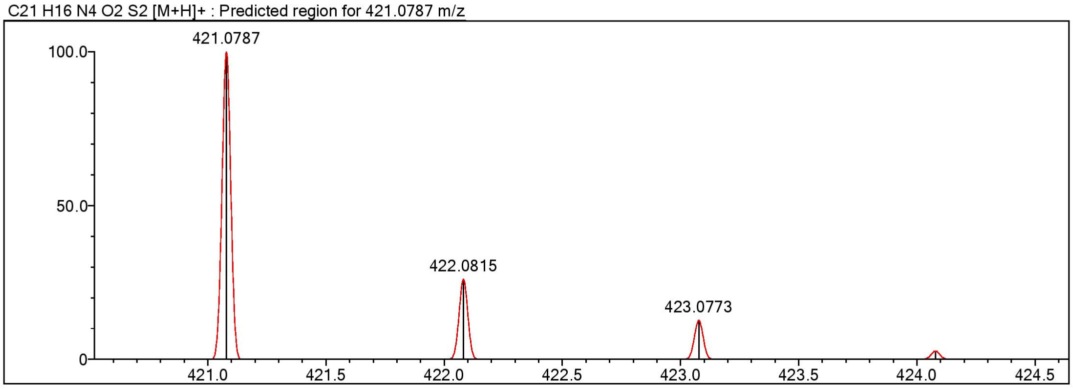

Rank	Score	Formula (M)	Ion	Meas. m/z	Pred. m/z	Df. (mDa)	Df. (ppm)	Iso	DBE
4	72.04	C21 H16 N4 O2 S2	[M+H]+	421.0794	421.0787	0.7	1.66	73.25	16.0

Figure S13. HRMS spectrum of compound **4e**

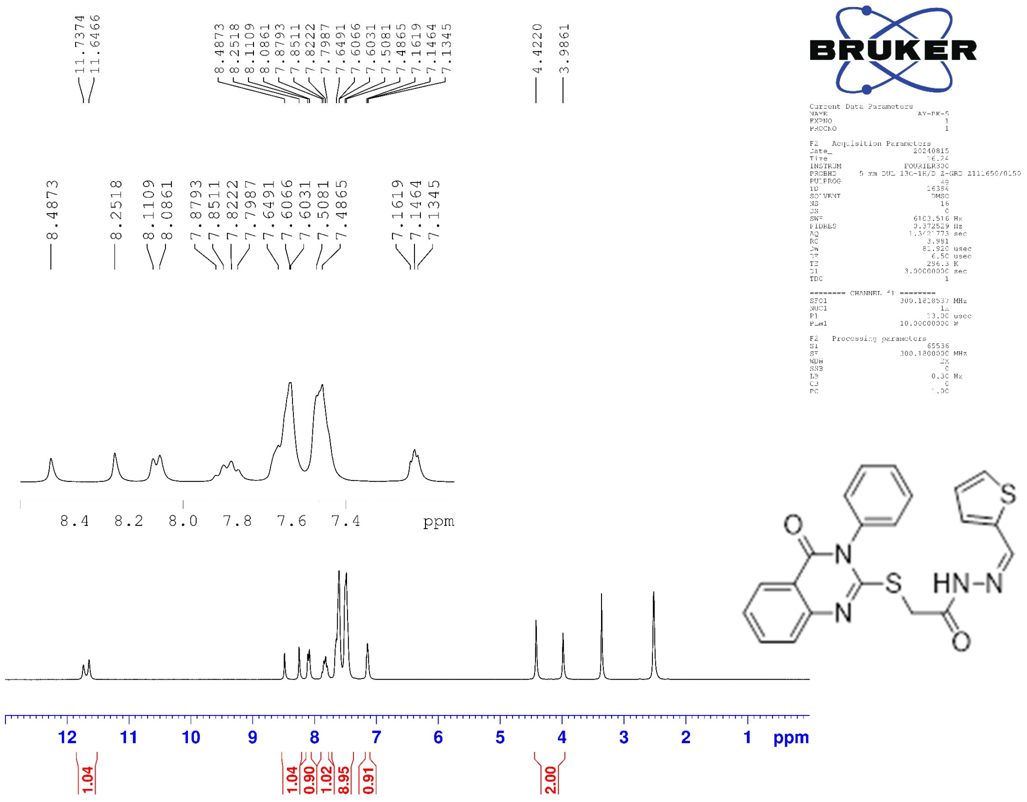

Figure S14. ^{1}H-NMR spectrum of compound **4e**

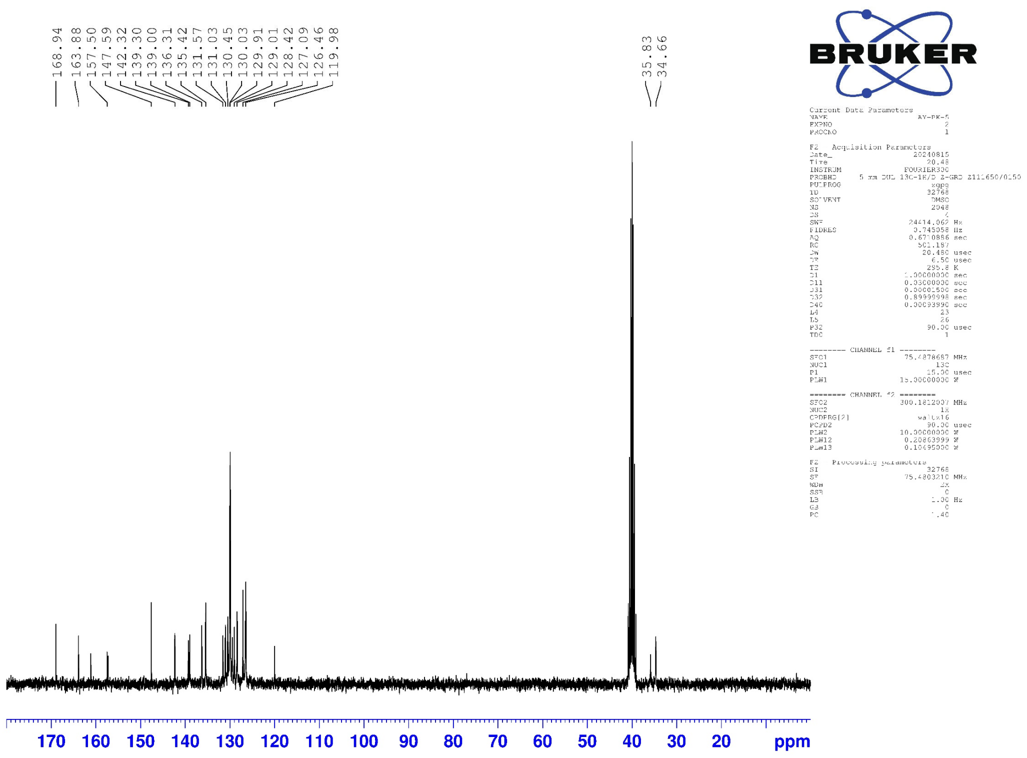

Figure S15. ^{13}C-NMR spectrum of compound **4e**

Data File: C:\LabSolutions\Data\Analiz\Asaf\AY-PK-6_503.lcd

Elmt	Val.	Min	Max	Elmt	Val.	Min	Max	Elmt	Val.	Min	Max	Elmt	Val.	Min	Max	Use Adduct
H	1	10	33	O	2	0	2	S	2	0	2	Ru	2	0	0	H
C	4	4	32	F	1	0	0	Cl	1	0	0	Pd	2	0	0	Na
N	3	0	5	P	3	0	0	Br	1	0	0	I	3	0	0	

Error Margin (ppm):	5	DBE Range:	0.0 - 30.0	Electron Ions:	both
HC Ratio:	unlimited	Apply N Rule:	no	Use MSn Info:	yes
Max Isotopes:	3	Isotope RI (%):	1.00	Isotope Res:	9000
MSn Iso RI (%):	10.00	MSn Logic Mode:	AND	Max Results:	50

Event#: 1 MS(E+) Ret. Time : 5.280 Scan# : 793

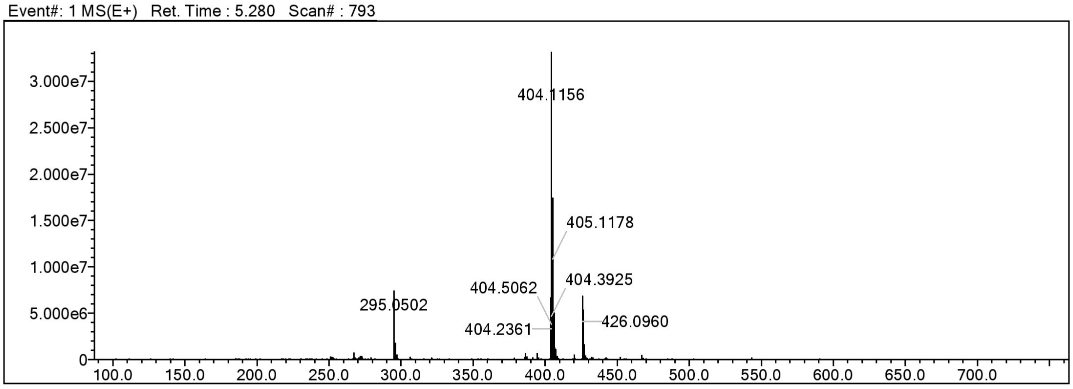

Measured region for 404.1156 m/z

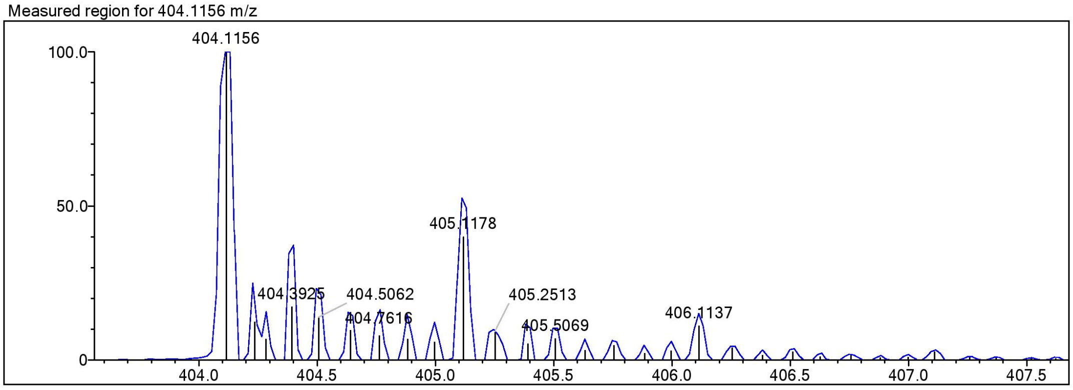

C21 H17 N5 O2 S [M+H]+ : Predicted region for 404.1176 m/z

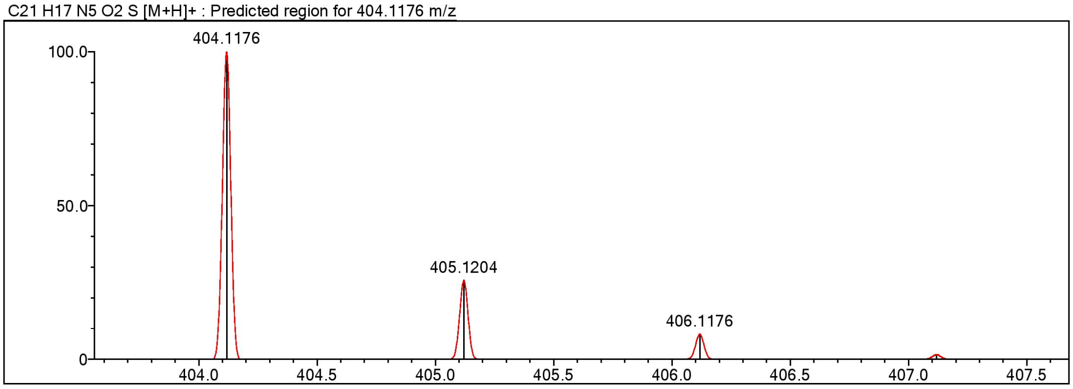

Rank	Score	Formula (M)	Ion	Meas. m/z	Pred. m/z	Df. (mDa)	Df. (ppm)	Iso	DBE
5	39.44	C21 H17 N5 O2 S	[M+H]+	404.1156	404.1176	-2.0	-4.95	43.76	16.0

Figure S16. HRMS spectrum of compound **4f**

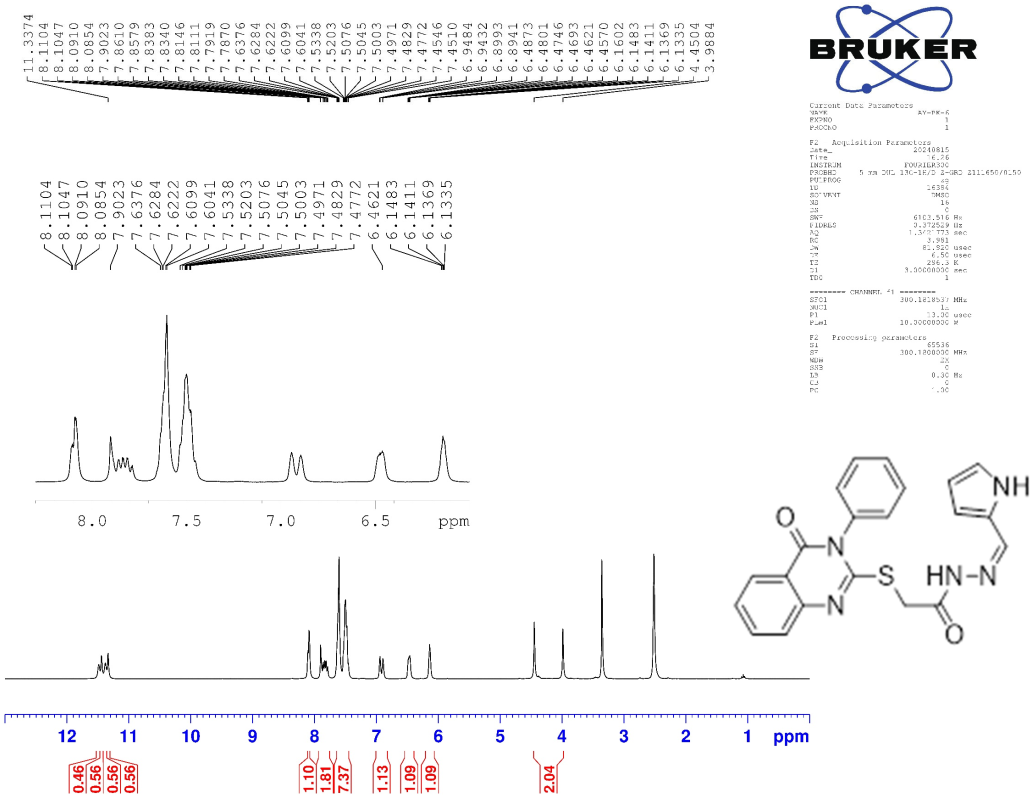

Figure S17. ^{1}H-NMR spectrum of compound **4f**

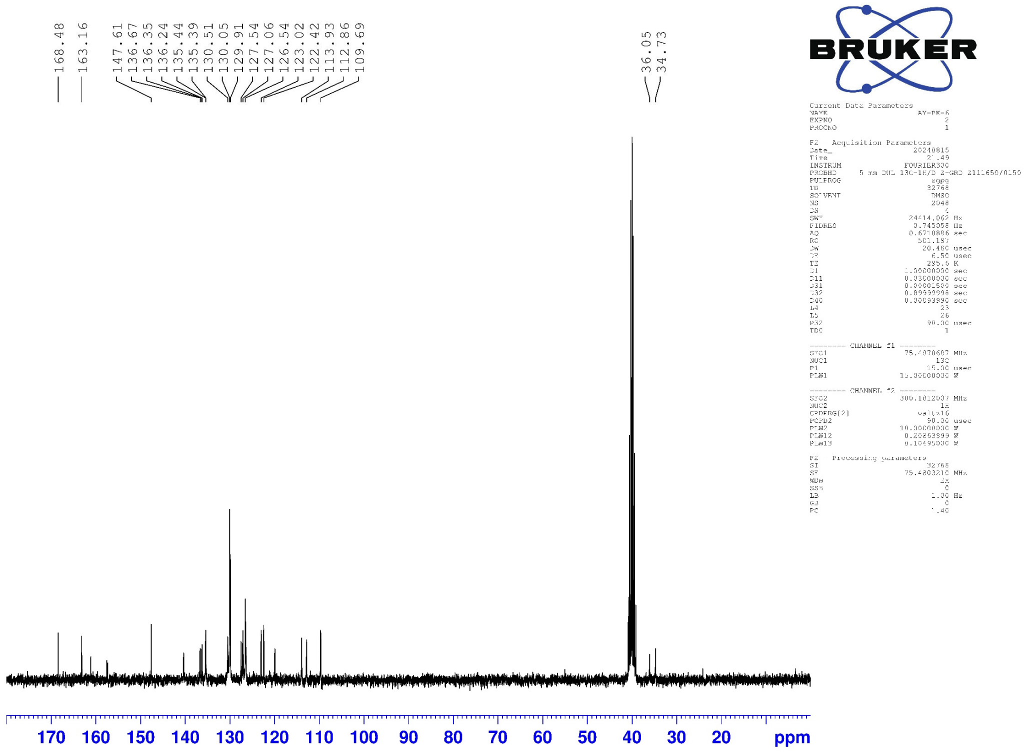

Figure S18. ^{13}C-NMR spectrum of compound **4f**

Data File: C:\LabSolutions\Data\Analiz\Asaf\AY-PK-7_2.lcd

Elmt	Val.	Min	Max	Elmt	Val.	Min	Max	Elmt	Val.	Min	Max	Elmt	Val.	Min	Max	Use Adduct
H	1	8	35	O	2	0	2	S	2	0	2	Ru	2	0	0	H
C	4	9	30	F	1	0	0	Cl	1	1	1	Pd	2	0	0	Na
N	3	5	5	P	3	0	0	Br	1	0	0	I	3	0	0	

Error Margin (ppm):	20	DBE Range:	0.0 - 20.0	Electron Ions:	both
HC Ratio:	unlimited	Apply N Rule:	no	Use MSn Info:	yes
Max Isotopes:	3	Isotope RI (%):	1.00	Isotope Res:	9000
MSn Iso RI (%):	10.00	MSn Logic Mode:	AND	Max Results:	50

Event#: 1 MS(E+) Ret. Time : 3.293 Scan# : 495

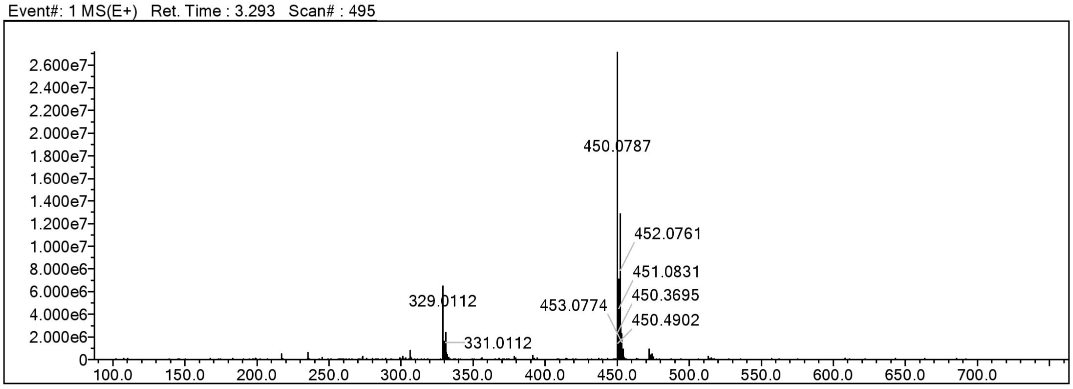

Measured region for 450.0787 m/z

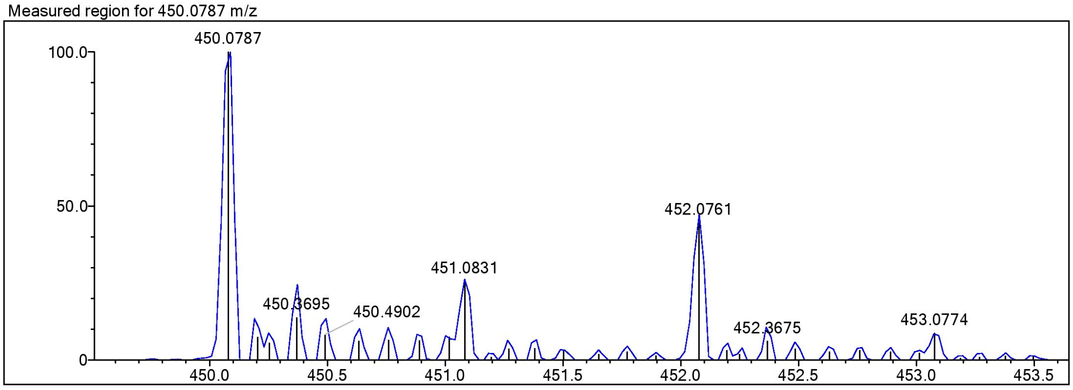

C22 H16 N5 O2 S Cl [M+H]+ : Predicted region for 450.0786 m/z

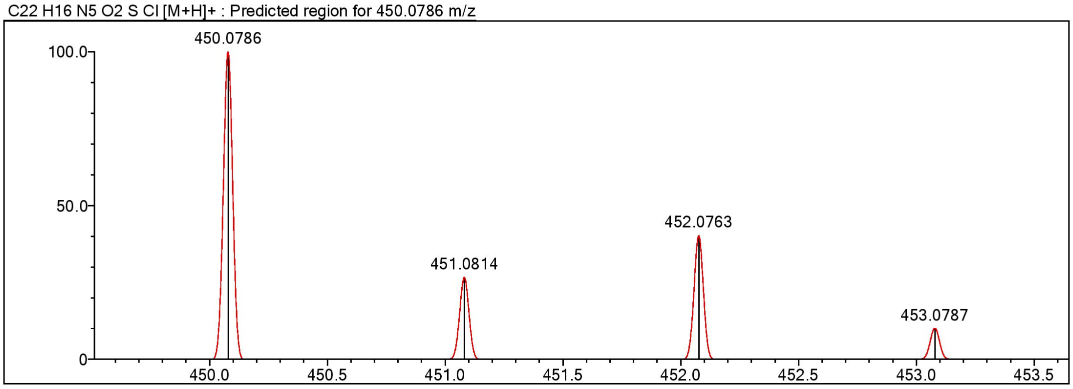

Rank	Score	Formula (M)	Ion	Meas. m/z	Pred. m/z	Df. (mDa)	Df. (ppm)	Iso	DBE
2	81.89	C22 H16 N5 O2 S Cl	[M+H]+	450.0787	450.0786	0.1	0.22	81.89	17.0

Figure S19. HRMS spectrum of compound **4g**

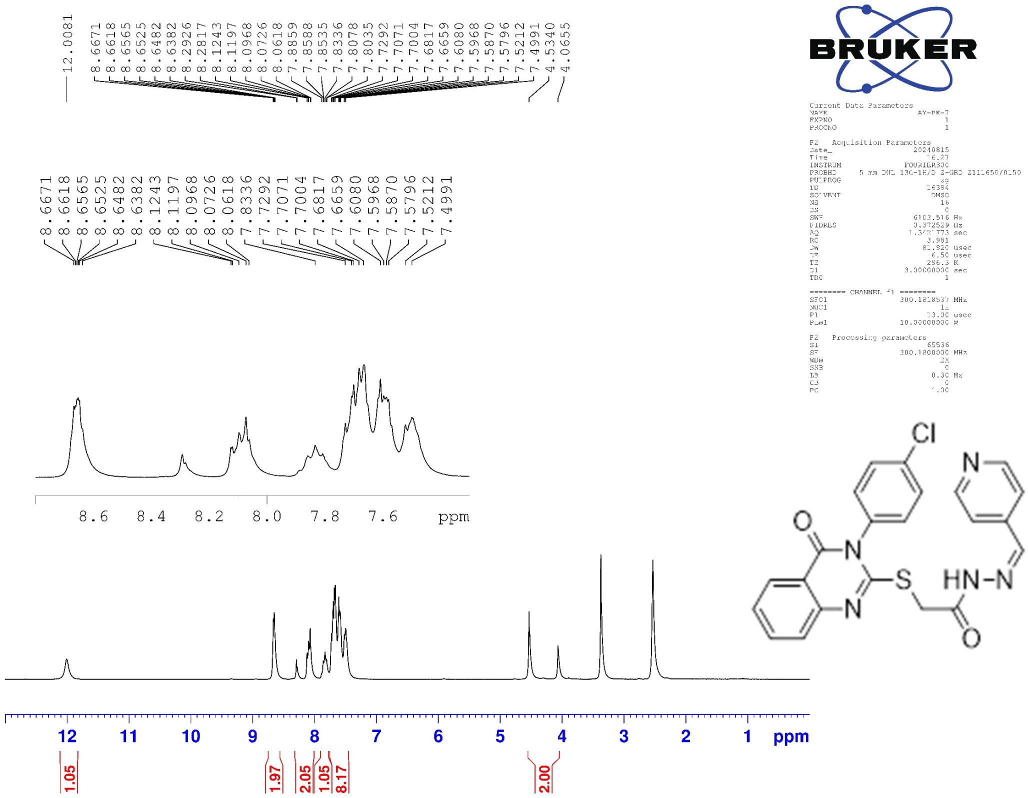

Figure S20. ^{1}H-NMR spectrum of compound **4g**

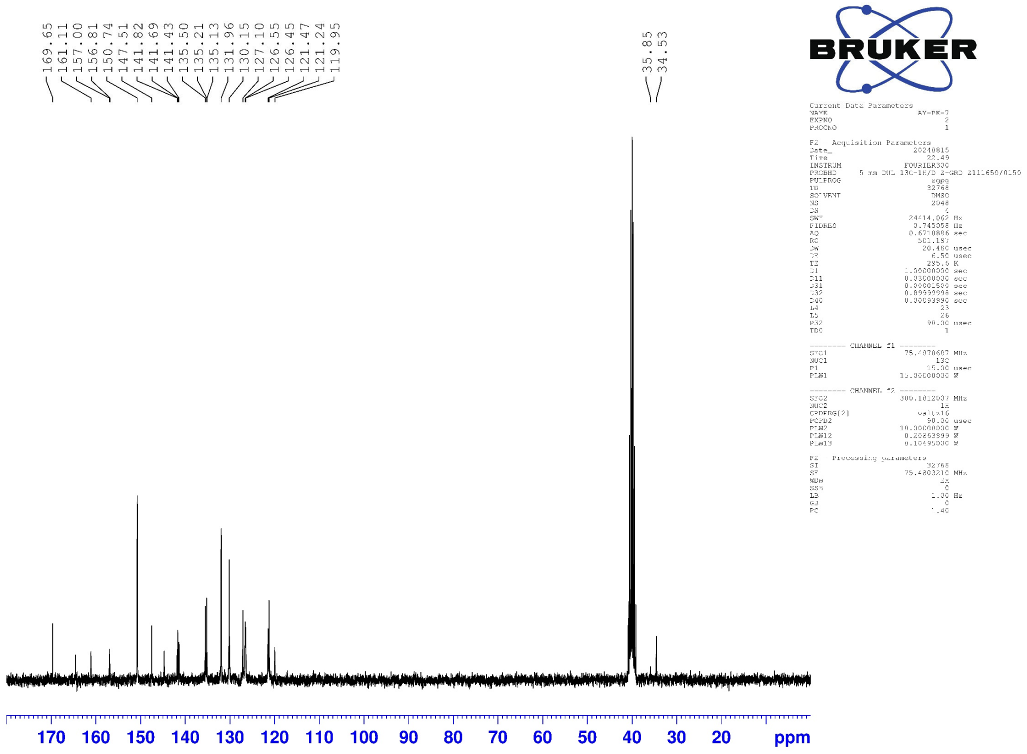

Figure S21. ^{13}C-NMR spectrum of compound **4g**

Data File: C:\LabSolutions\Data\Analiz\Asaf\AY-PK-8_504.lcd

Elmt	Val.	Min	Max	Elmt	Val.	Min	Max	Elmt	Val.	Min	Max	Elmt	Val.	Min	Max	Use Adduct
H	1	10	33	O	2	0	5	S	2	1	1	Ru	2	0	0	H
C	4	4	32	F	1	0	0	Cl	1	1	1	Pd	2	0	0	Na
N	3	0	6	P	3	0	0	Br	1	0	0	I	3	0	0	

Error Margin (ppm):	5	DBE Range:	0.0 - 30.0	Electron Ions:	both
HC Ratio:	unlimited	Apply N Rule:	no	Use MSn Info:	yes
Max Isotopes:	3	Isotope RI (%):	1.00	Isotope Res:	9000
MSn Iso RI (%):	10.00	MSn Logic Mode:	AND	Max Results:	50

Event#: 1 MS(E+) Ret. Time : 5.467 Scan# : 821

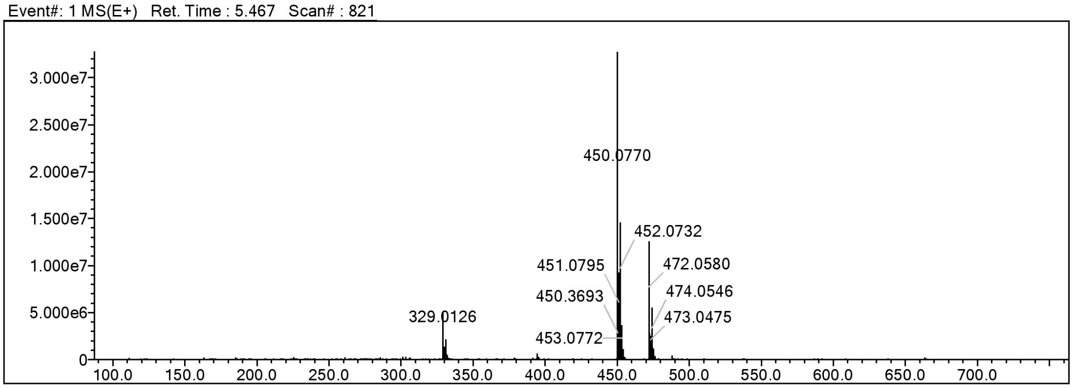

Measured region for 450.0770 m/z

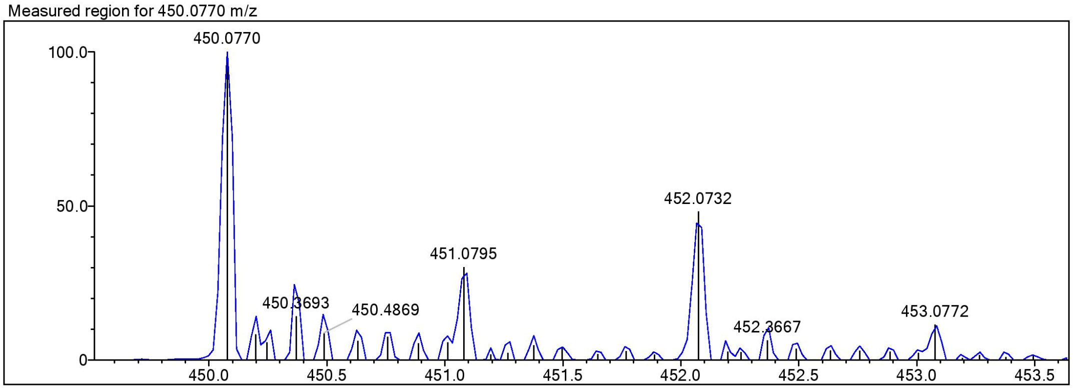

C22 H16 N5 O2 S Cl [M+H]+ : Predicted region for 450.0786 m/z

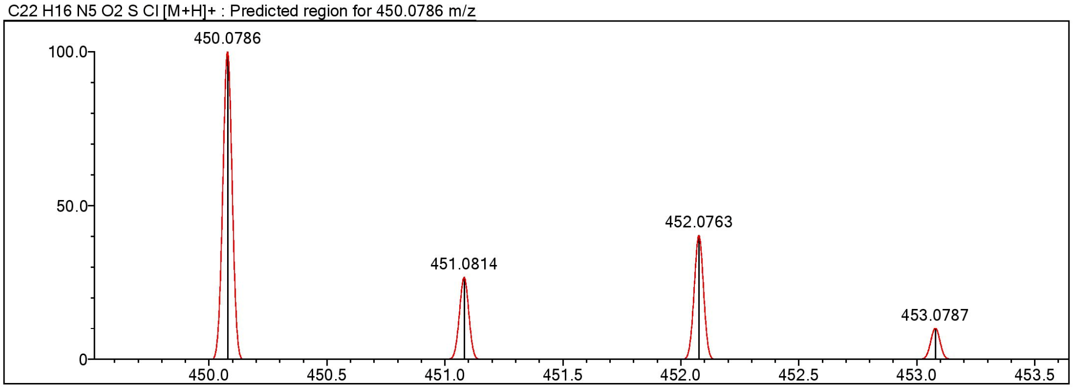

Rank	Score	Formula (M)	Ion	Meas. m/z	Pred. m/z	Df. (mDa)	Df. (ppm)	Iso	DBE
2	88.85	C22 H16 N5 O2 S Cl	[M+H]+	450.0770	450.0786	-1.6	-3.55	94.90	17.0

Figure S22. HRMS spectrum of compound **4h**

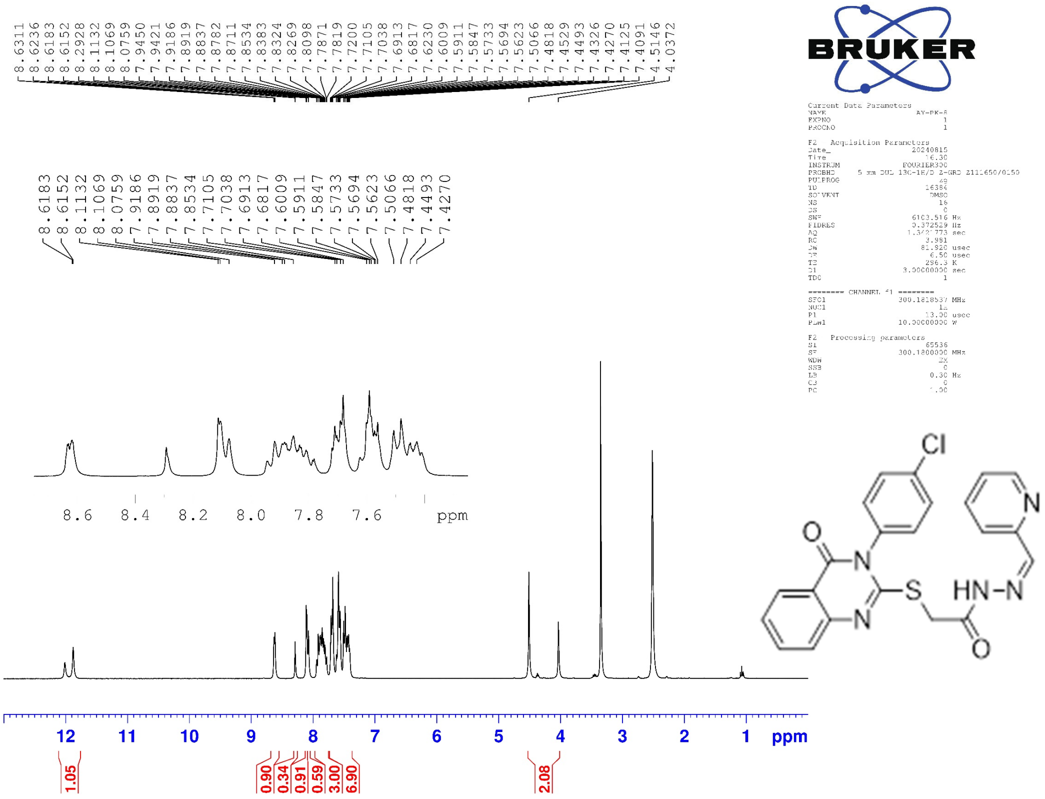

Figure S23. ^{1}H-NMR spectrum of compound **4h**

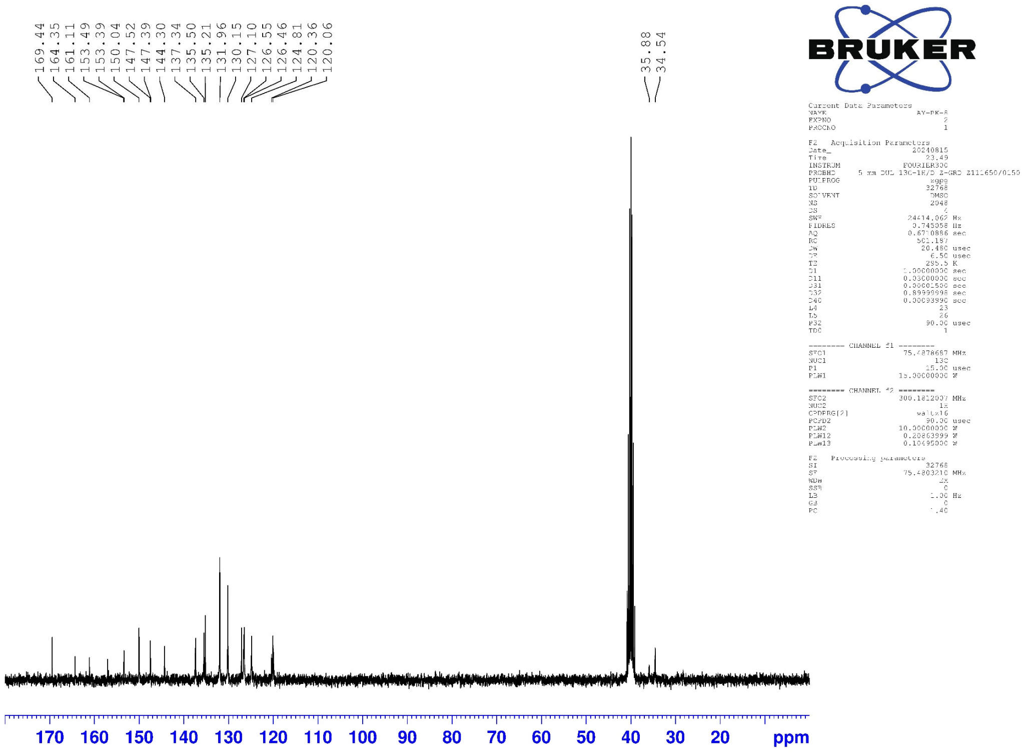

Figure S24. ^{13}C-NMR spectrum of compound **4h**

Data File: C:\LabSolutions\Data\Analiz\Asaf\AY-PK-9_505.lcd

Elmt	Val.	Min	Max	Elmt	Val.	Min	Max	Elmt	Val.	Min	Max	Elmt	Val.	Min	Max	Use Adduct
H	1	10	33	O	2	0	5	S	2	2	2	Ru	2	0	0	H
C	4	4	32	F	1	0	0	Cl	1	1	1	Pd	2	0	0	Na
N	3	0	4	P	3	0	0	Br	1	0	0	I	3	0	0	

Error Margin (ppm):	5	DBE Range:	0.0 - 30.0	Electron Ions:	both
HC Ratio:	unlimited	Apply N Rule:	no	Use MSn Info:	yes
Max Isotopes:	3	Isotope RI (%):	1.00	Isotope Res:	9000
MSn Iso RI (%):	10.00	MSn Logic Mode:	AND	Max Results:	50

Event#: 1 MS(E+) Ret. Time : 7.093 Scan# : 1065

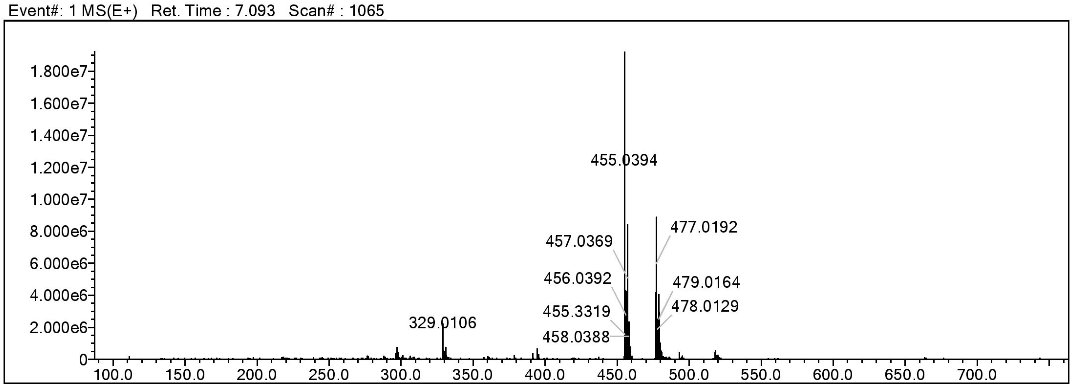

Measured region for 455.0394 m/z

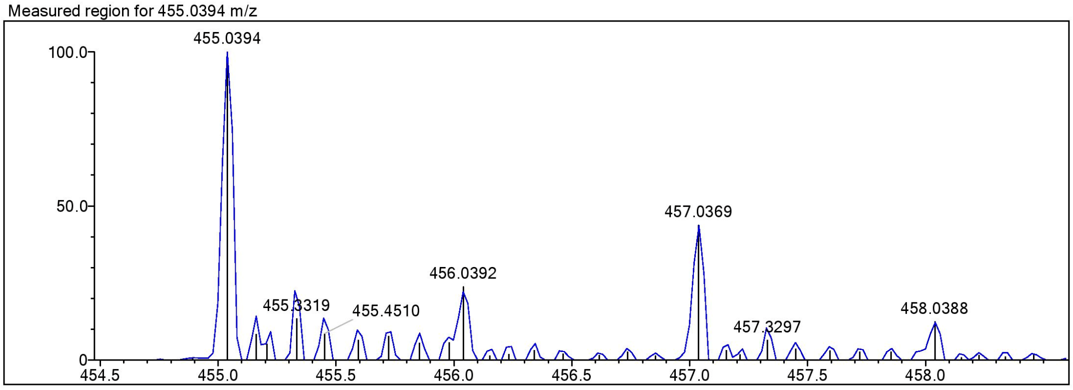

C21 H15 N4 O2 S2 Cl [M+H]+ : Predicted region for 455.0398 m/z

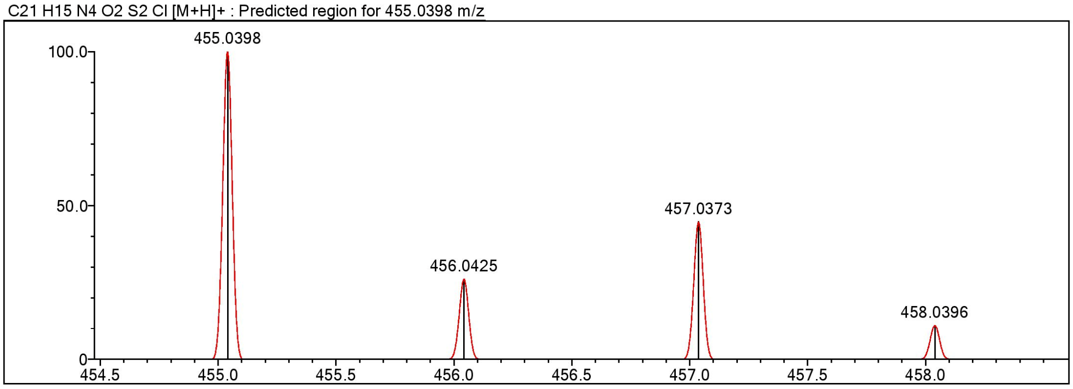

Rank	Score	Formula (M)	Ion	Meas. m/z	Pred. m/z	Df. (mDa)	Df. (ppm)	Iso	DBE
2	75.88	C21 H15 N4 O2 S2 Cl	[M+H]+	455.0394	455.0398	-0.4	-0.88	75.88	16.0

Figure S25. HRMS spectrum of compound **4i**

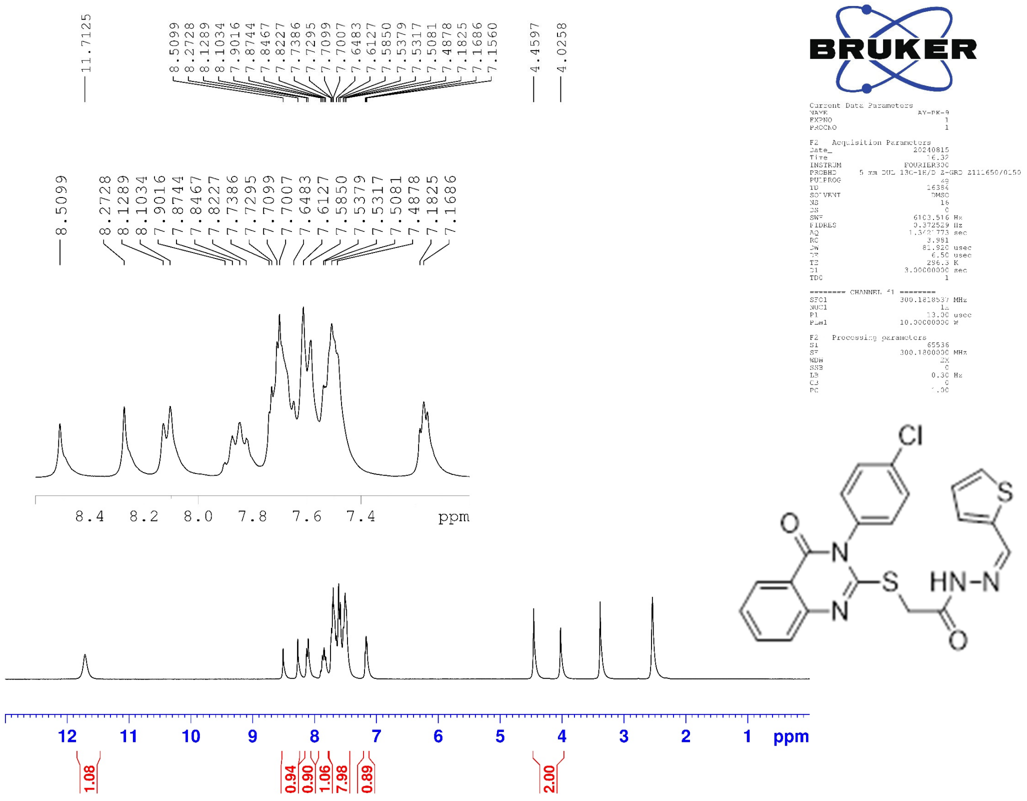

Figure S26. ^{1}H-NMR spectrum of compound **4i**

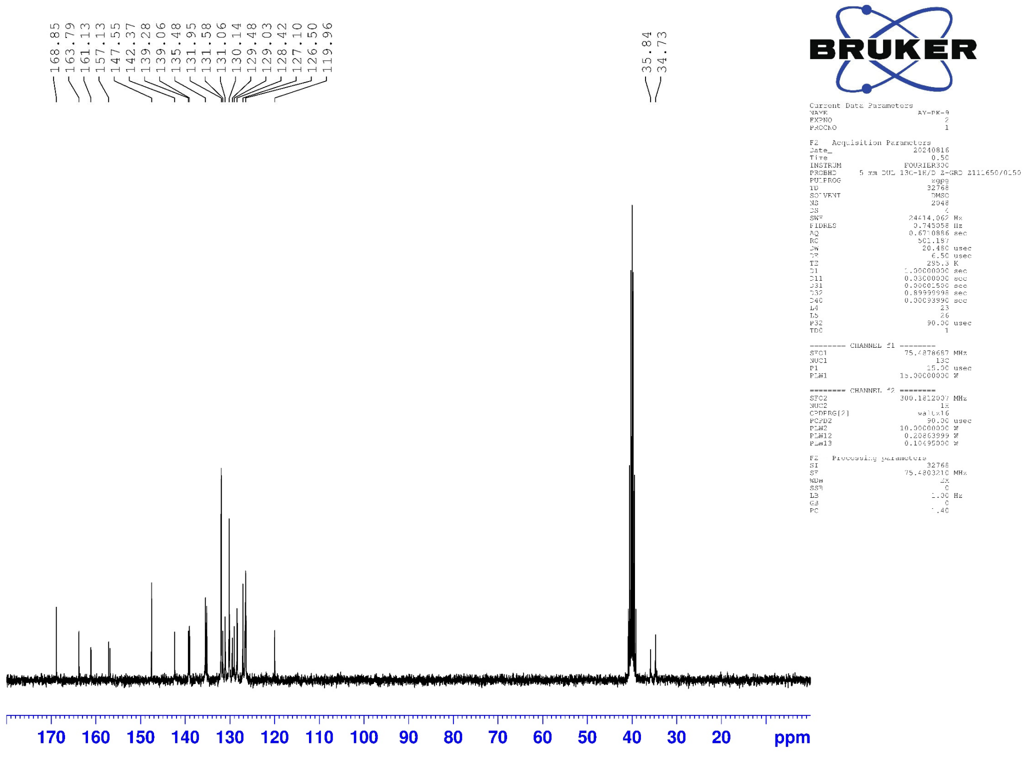

Figure S27. ^{13}C-NMR spectrum of compound **4i**

Data File: C:\LabSolutions\Data\Analiz\Asaf\AY-PK-10_506.lcd

Elmt	Val.	Min	Max	Elmt	Val.	Min	Max	Elmt	Val.	Min	Max	Elmt	Val.	Min	Max	Use Adduct
H	1	10	33	O	2	0	2	S	2	0	2	Ru	2	0	0	H
C	4	4	32	F	1	0	0	Cl	1	1	1	Pd	2	0	0	Na
N	3	0	5	P	3	0	0	Br	1	0	0	I	3	0	0	

Error Margin (ppm):	5	DBE Range:	0.0 - 30.0	Electron Ions:	both
HC Ratio:	unlimited	Apply N Rule:	no	Use MSn Info:	yes
Max Isotopes:	3	Isotope RI (%):	1.00	Isotope Res:	9000
MSn Iso RI (%):	10.00	MSn Logic Mode:	AND	Max Results:	50

Event#: 1 MS(E+) Ret. Time : 5.800 Scan# : 871

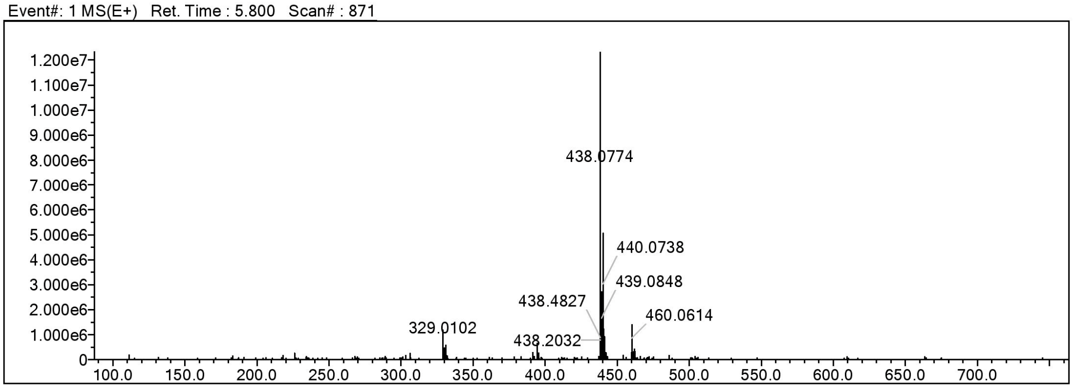

Measured region for 438.0774 m/z

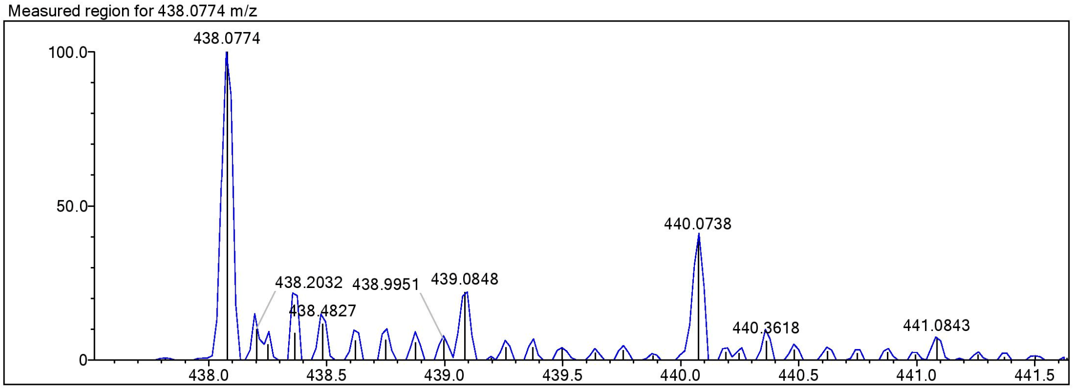

C21 H16 N5 O2 S Cl [M+H]+ : Predicted region for 438.0786 m/z

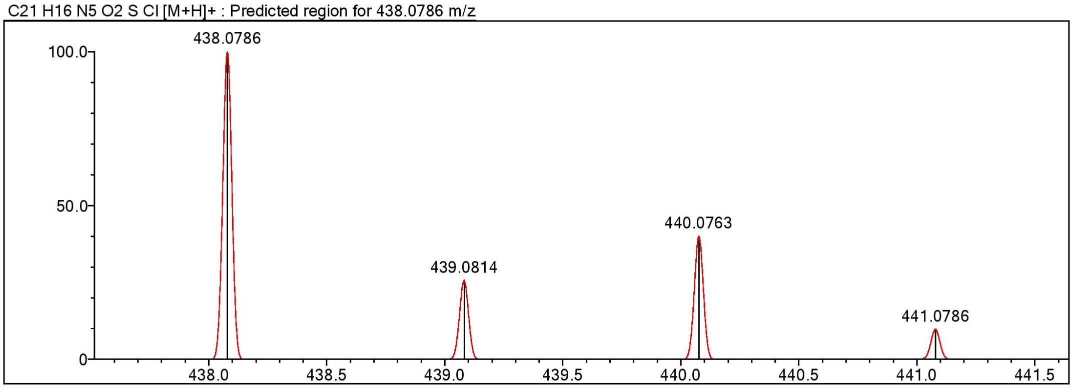

Rank	Score	Formula (M)	Ion	Meas. m/z	Pred. m/z	Df. (mDa)	Df. (ppm)	Iso	DBE
3	73.20	C21 H16 N5 O2 S Cl	[M+H]+	438.0774	438.0786	-1.2	-2.74	76.53	16.0

Figure S28. HRMS spectrum of compound **4j**

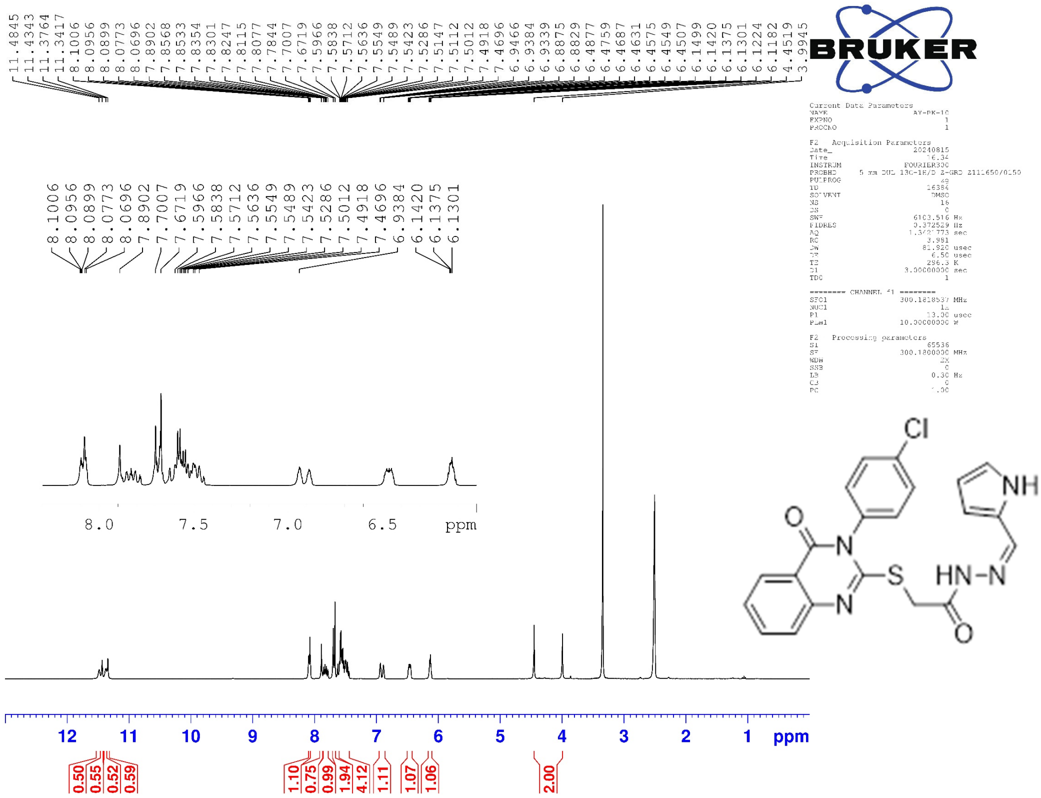

Figure S29. ^{1}H-NMR spectrum of compound **4j**

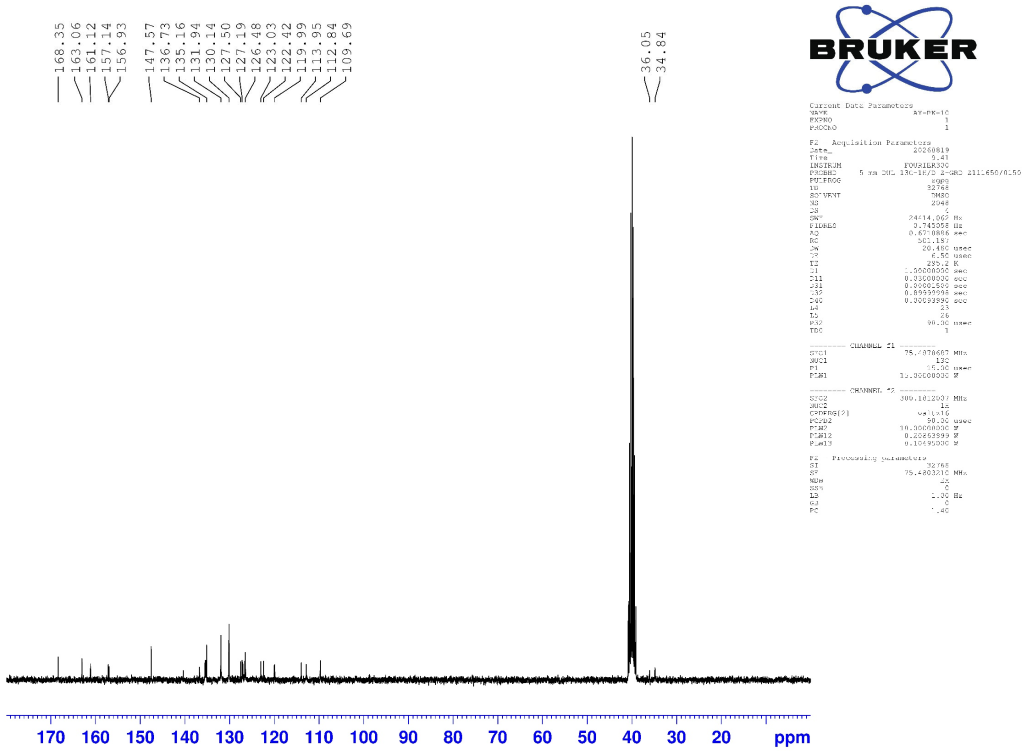

Figure S30. ^{13}C-NMR spectrum of compound **4j**

Data File: C:\LabSolutions\Data\Analiz\Asaf\AY-PK-23_531.lcd

Elmt	Val.	Min	Max	Elmt	Val.	Min	Max	Elmt	Val.	Min	Max	Elmt	Val.	Min	Max	Use Adduct
H	1	8	35	O	2	0	3	S	2	0	2	Ru	2	0	0	H
C	4	9	30	F	1	0	0	Cl	1	1	1	Pd	2	0	0	Na
N	3	0	5	P	3	0	0	Br	1	0	0	I	3	0	0	

Error Margin (ppm):	5	DBE Range:	0.0 - 20.0	Electron Ions:	both
HC Ratio:	unlimited	Apply N Rule:	no	Use MSn Info:	yes
Max Isotopes:	3	Isotope RI (%):	1.00	Isotope Res:	9000
MSn Iso RI (%):	10.00	MSn Logic Mode:	AND	Max Results:	50

Event#: 1 MS(E+) Ret. Time : 4.653 -> 5.213 - 6.280 -> 8.440 Scan# : 699 -> 783 - 943 -> 1267

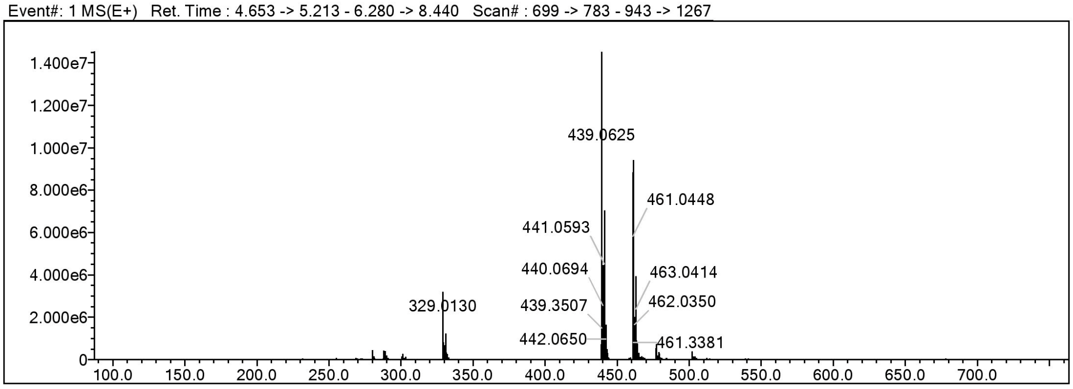

Measured region for 439.0625 m/z

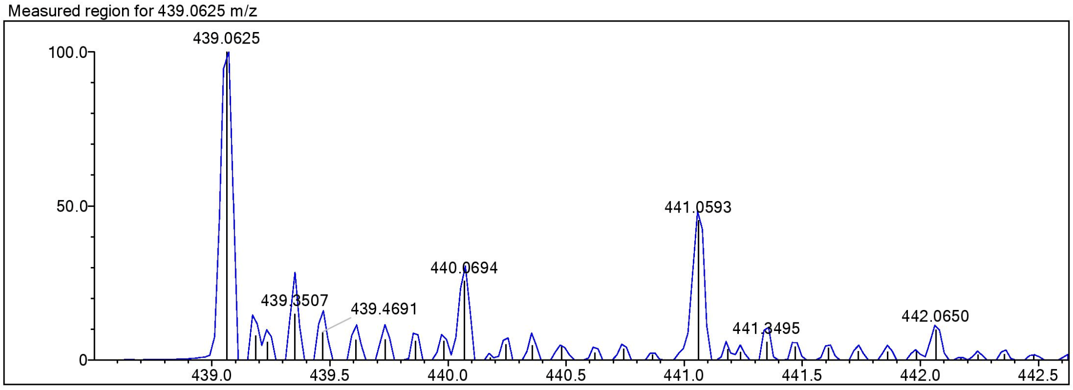

C21 H15 N4 O3 S Cl [M+H]+ : Predicted region for 439.0626 m/z

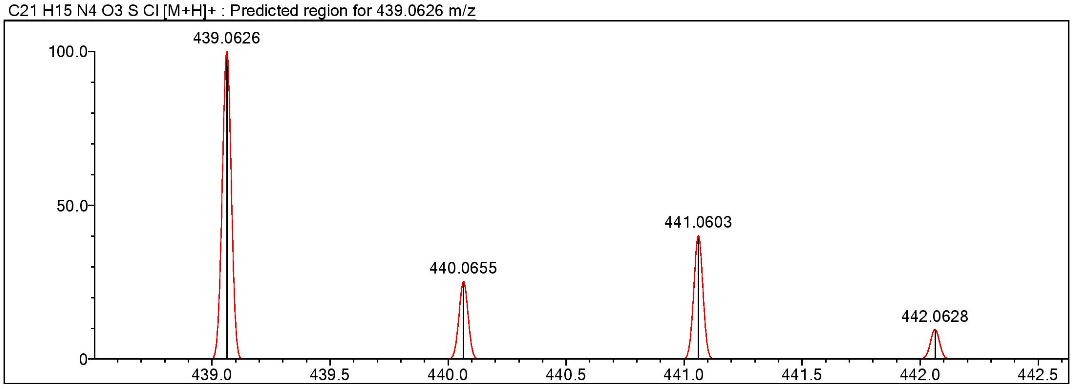

Rank	Score	Formula (M)	Ion	Meas. m/z	Pred. m/z	Df. (mDa)	Df. (ppm)	Iso	DBE
3	81.24	C21 H15 N4 O3 S Cl	[M+H]+	439.0625	439.0626	-0.1	-0.23	81.24	16.0

Figure S31. HRMS spectrum of compound **4k**

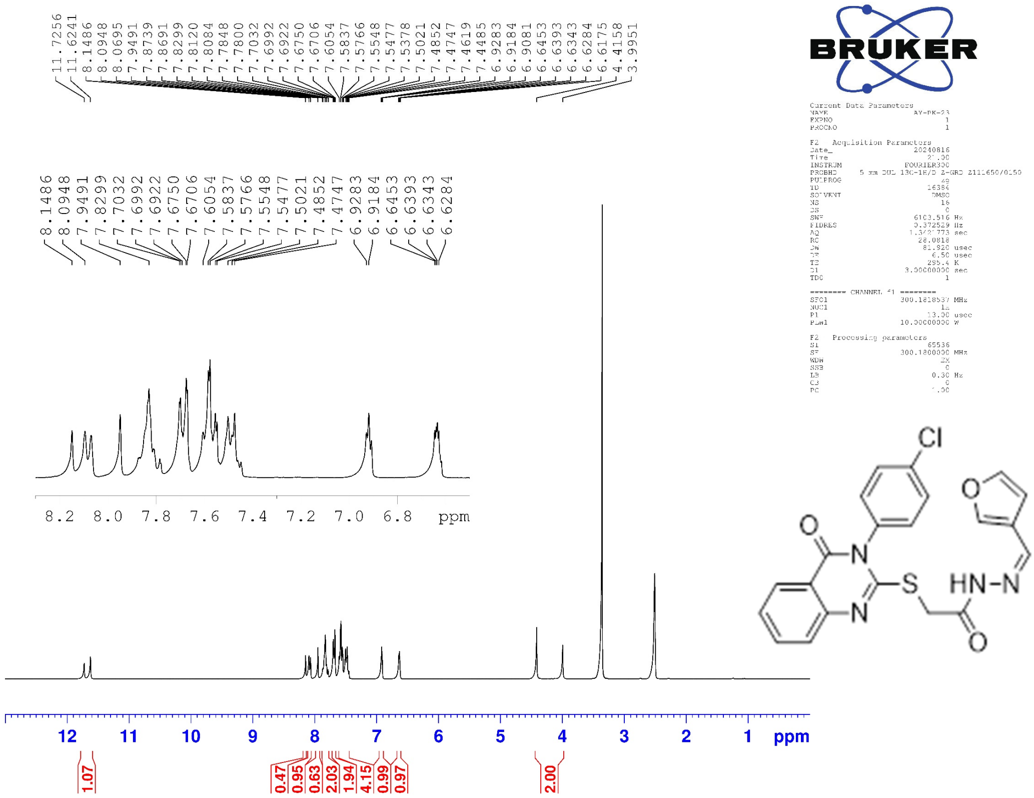

Figure S32. ^{1}H-NMR spectrum of compound **4k**

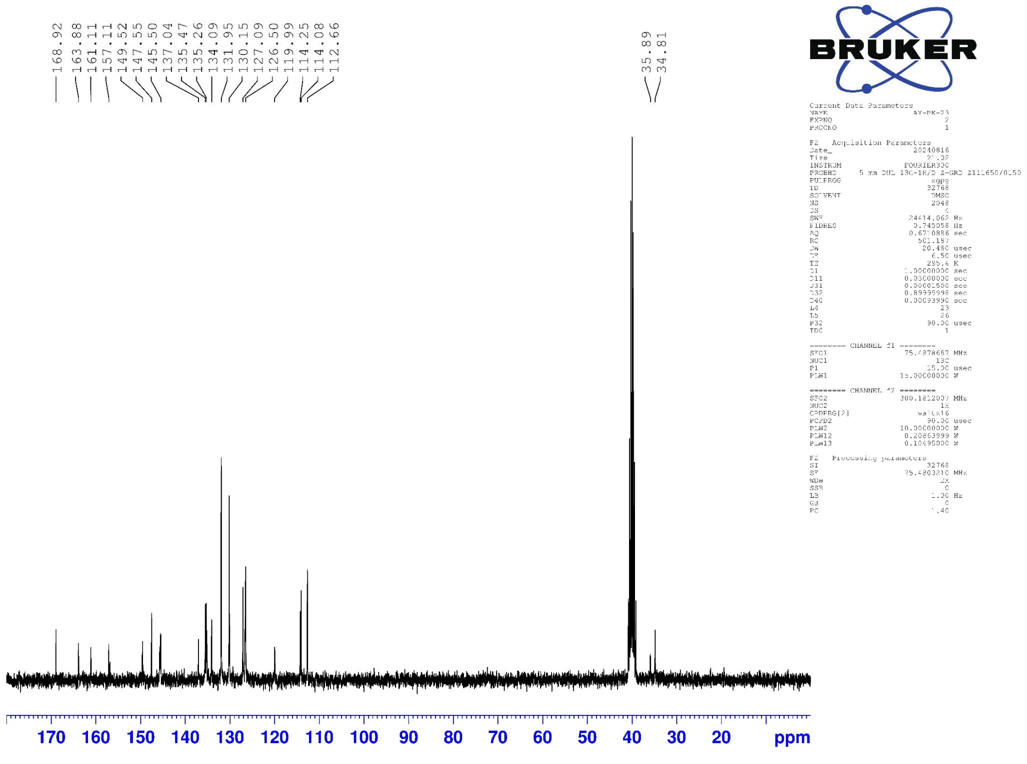

Figure S33. ^{13}C-NMR spectrum of compound **4k**

Data File: C:\LabSolutions\Data\Analiz\Asaf\AY-PK-22_530.lcd

Elmt	Val.	Min	Max	Elmt	Val.	Min	Max	Elmt	Val.	Min	Max	Elmt	Val.	Min	Max	Use Adduct
H	1	8	35	O	2	0	3	S	2	0	2	Ru	2	0	0	H
C	4	9	30	F	1	0	0	Cl	1	1	1	Pd	2	0	0	Na
N	3	0	5	P	3	0	0	Br	1	0	0	I	3	0	0	

Error Margin (ppm):	5	DBE Range:	0.0 - 20.0	Electron Ions:	both
HC Ratio:	unlimited	Apply N Rule:	no	Use MSn Info:	yes
Max Isotopes:	3	Isotope RI (%):	1.00	Isotope Res:	9000
MSn Iso RI (%):	10.00	MSn Logic Mode:	AND	Max Results:	50

Event#: 1 MS(E+) Ret. Time : 5.227 Scan# : 785

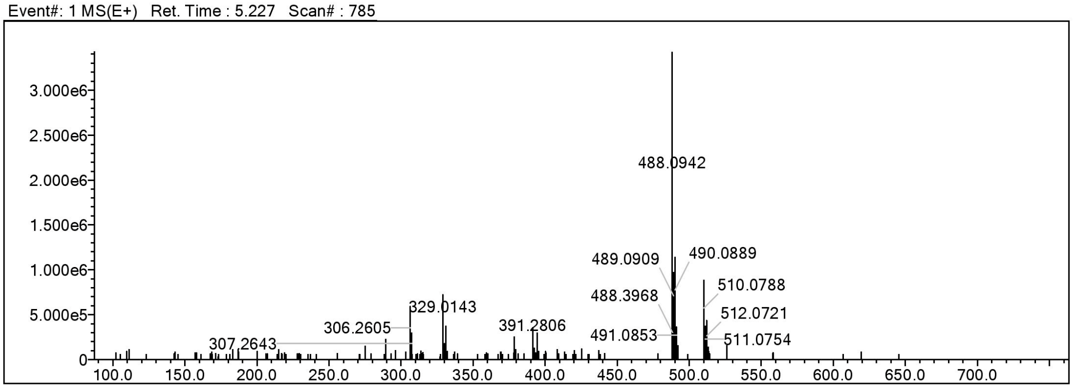

Measured region for 488.0942 m/z

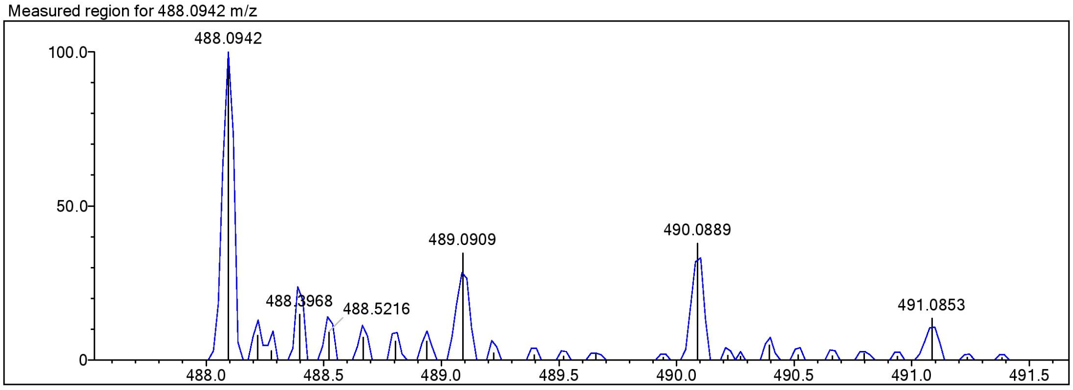

C25 H18 N5 O2 S Cl [M+H]+ : Predicted region for 488.0942 m/z

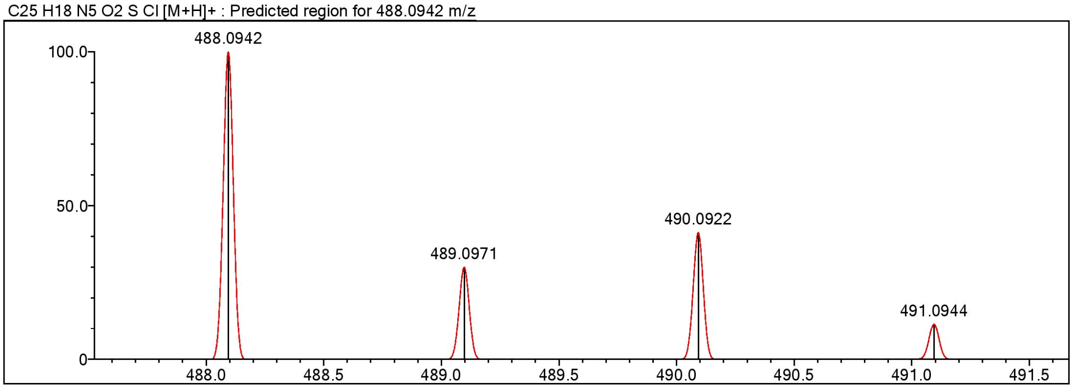

Rank	Score	Formula (M)	Ion	Meas. m/z	Pred. m/z	Df. (mDa)	Df. (ppm)	Iso	DBE
2	66.81	C25 H18 N5 O2 S Cl	[M+H]+	488.0942	488.0942	-0.0	0.00	66.81	19.0

Figure S34. HRMS spectrum of compound **4l**

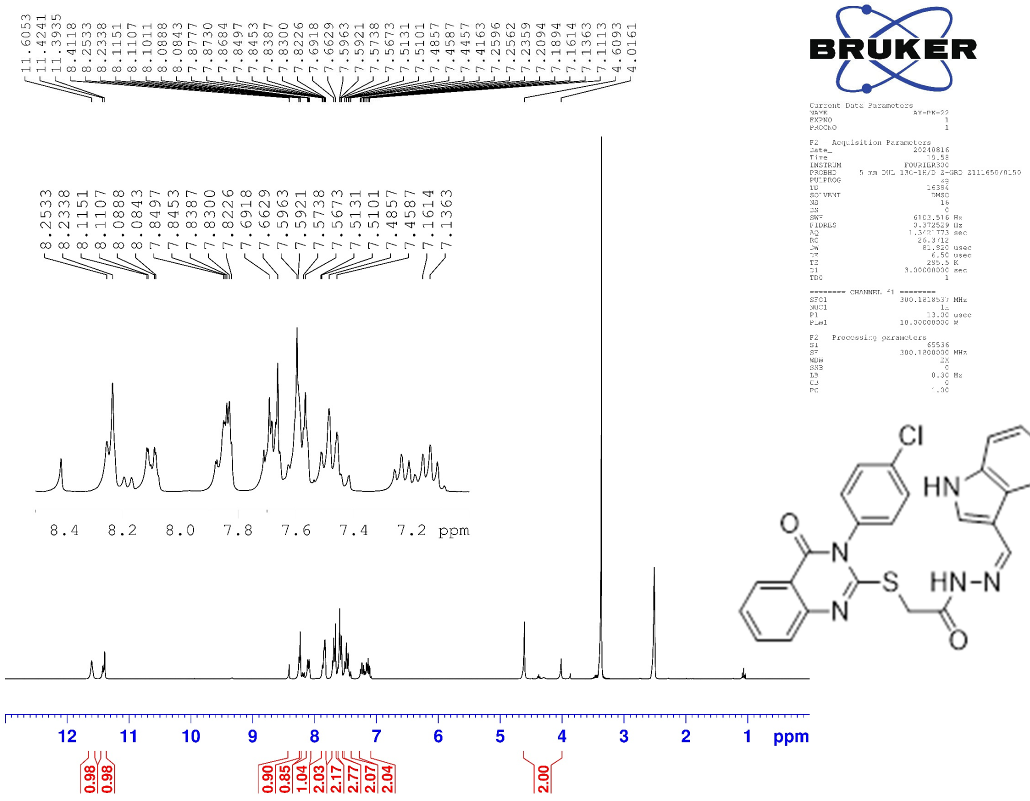

Figure S35. ^{1}H-NMR spectrum of compound **4l**

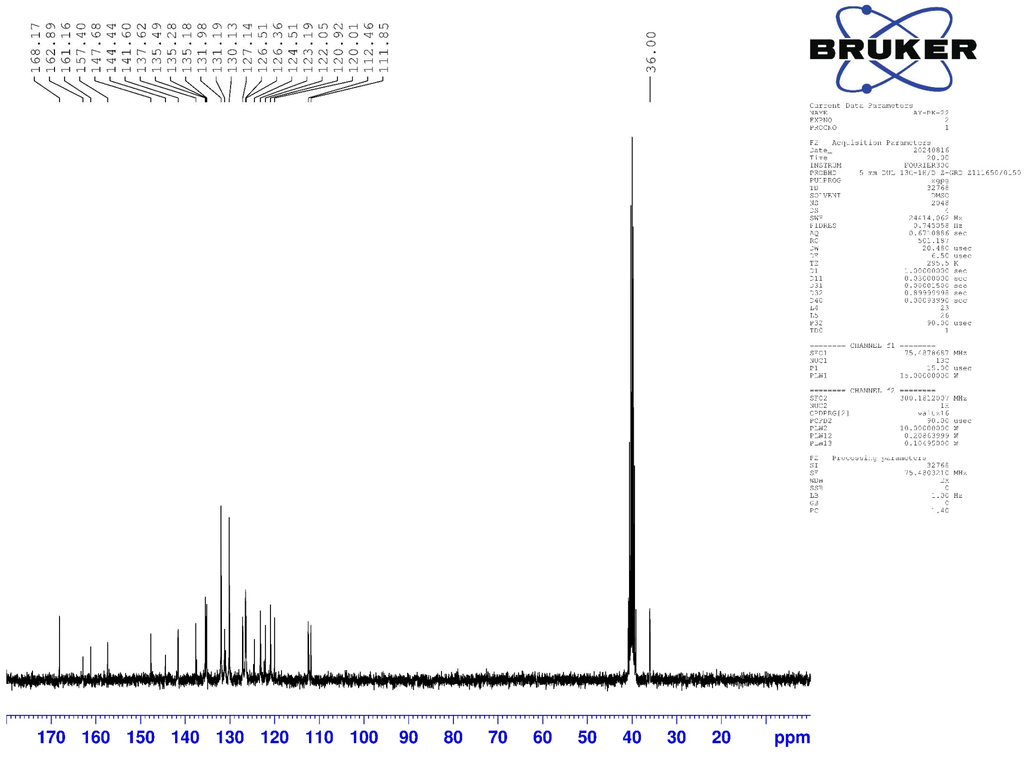

Figure S36. ^{13}C-NMR spectrum of compound **4l**

Data File: C:\LabSolutions\Data\Analiz\Asaf\AY-PK-20_528.lcd

Elmt	Val.	Min	Max	Elmt	Val.	Min	Max	Elmt	Val.	Min	Max	Elmt	Val.	Min	Max	Use Adduct
H	1	8	35	O	2	0	3	S	2	0	2	Ru	2	0	0	H
C	4	9	30	F	1	0	0	Cl	1	1	1	Pd	2	0	0	Na
N	3	0	5	P	3	0	0	Br	1	0	0	I	3	0	0	

Error Margin (ppm):	5	DBE Range:	0.0 - 20.0	Electron Ions:	both
HC Ratio:	unlimited	Apply N Rule:	no	Use MSn Info:	yes
Max Isotopes:	3	Isotope RI (%):	1.00	Isotope Res:	9000
MSn Iso RI (%):	10.00	MSn Logic Mode:	AND	Max Results:	50

Event#: 1 MS(E+) Ret. Time : 8.280 -> 9.413 - 4.907 -> 6.119 Scan# : 1243 -> 1413 - 737 -> 919

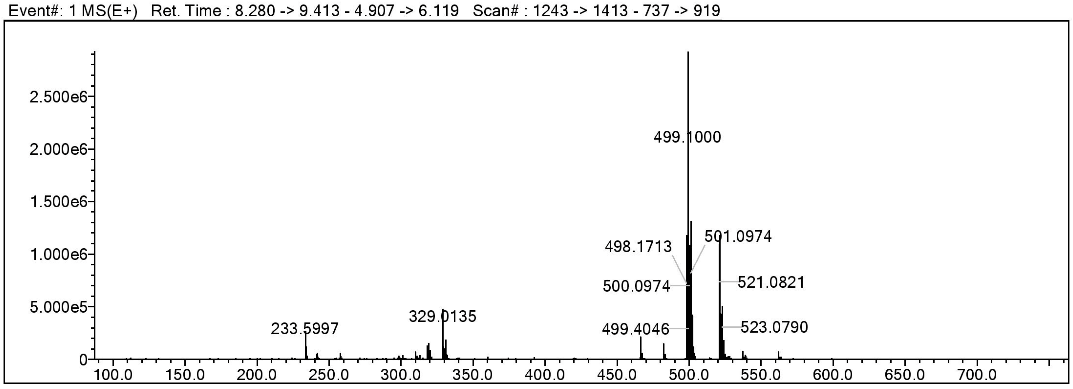

Measured region for 499.1000 m/z

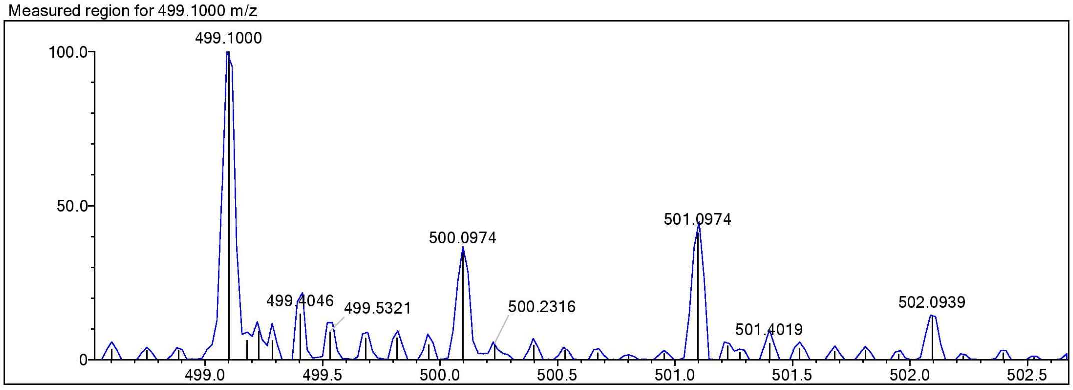

C27 H19 N4 O2 S Cl [M+H]+ : Predicted region for 499.0990 m/z

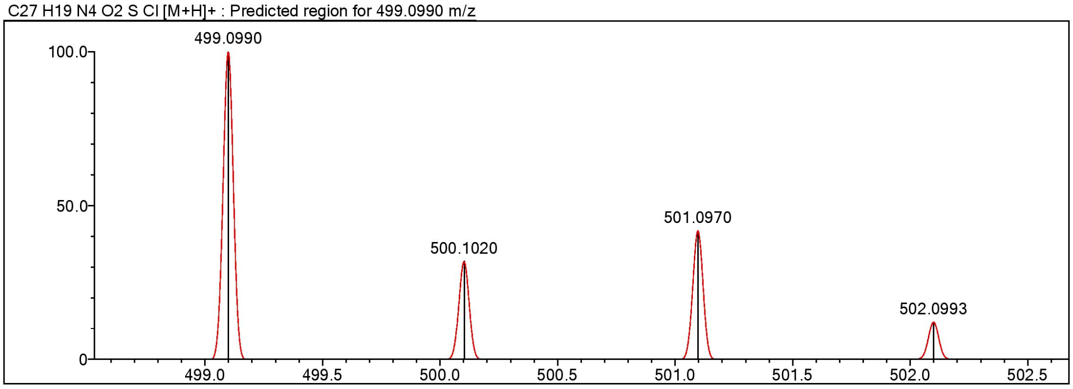

Rank	Score	Formula (M)	Ion	Meas. m/z	Pred. m/z	Df. (mDa)	Df. (ppm)	Iso	DBE
2	90.19	C27 H19 N4 O2 S Cl	[M+H]+	499.1000	499.0990	1.0	2.00	92.50	20.0

Figure S37. HRMS spectrum of compound **4m**

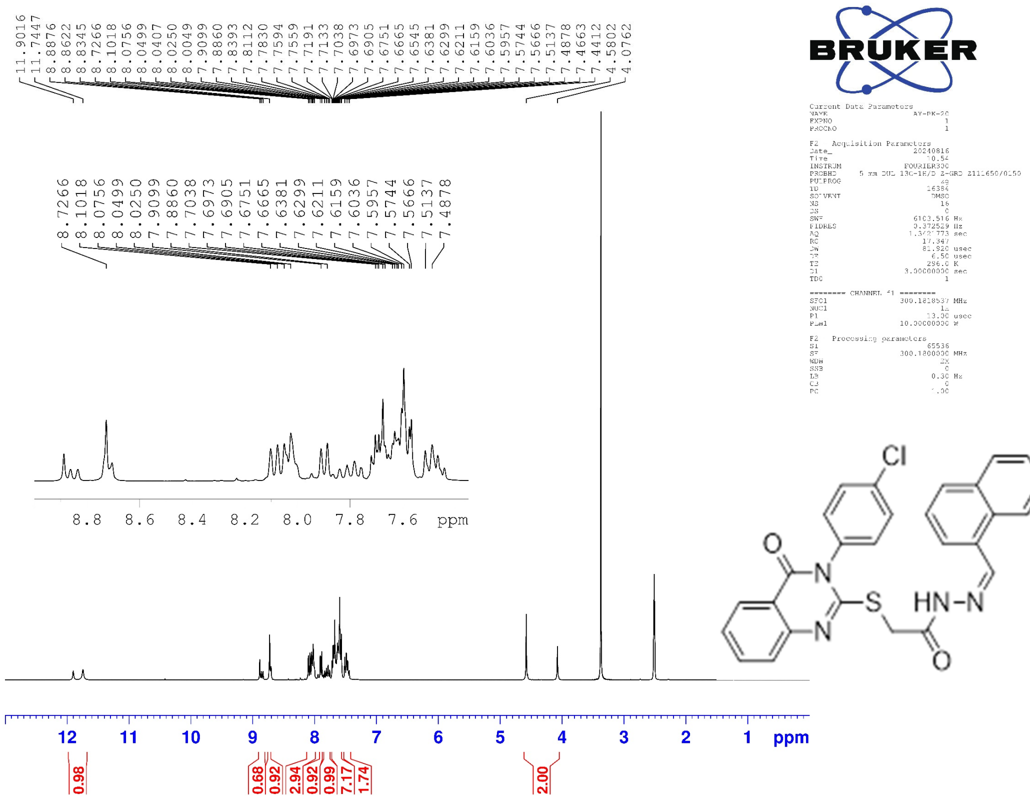

Figure S38. ^{1}H-NMR spectrum of compound **4m**

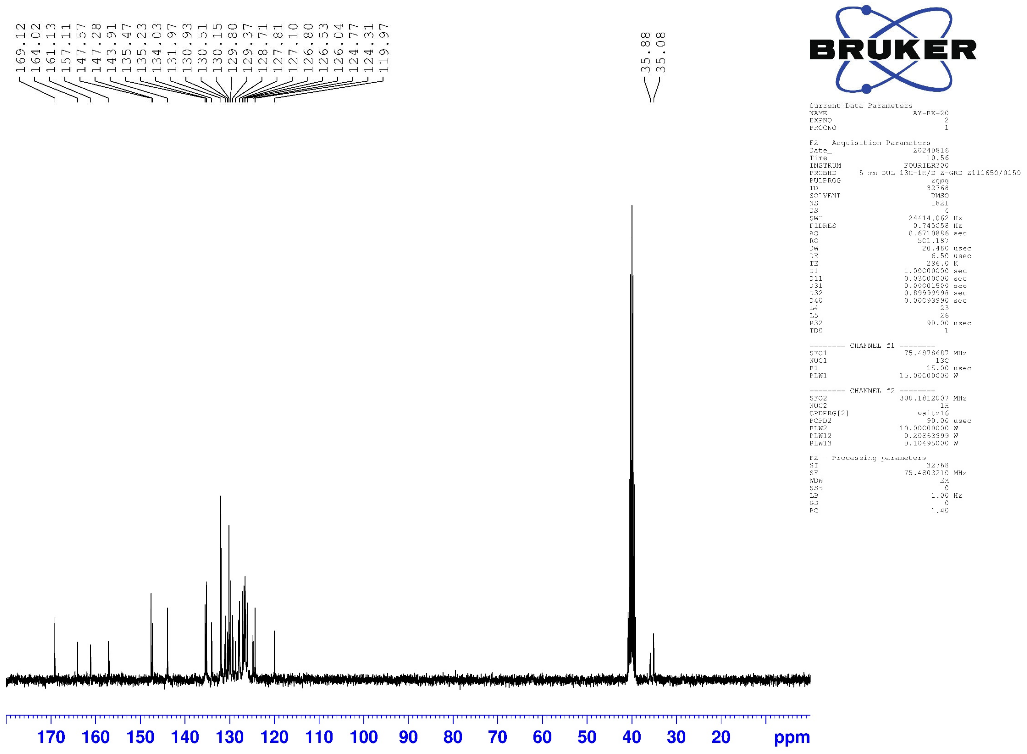

Figure S39. ^{13}C-NMR spectrum of compound **4m**

DOPNALAB

	Item	Value
	Acquired Date&Time	21.08.2026 11:38:16
	Acquired by	System Administrator
	Filename	C:\Users\dopnalab\Desktop\MASAÜSTÜ\FARMASÖTİK\aybuke\AY-PK-212.ispd
	Spectrum name	AY-PK-212
	Sample name	AY-PK-21
	Sample ID	
	Option	
	Comment	
	No. of Scans	30
	Resolution	4 [cm-1]
	Apodization	Happ-Genzel

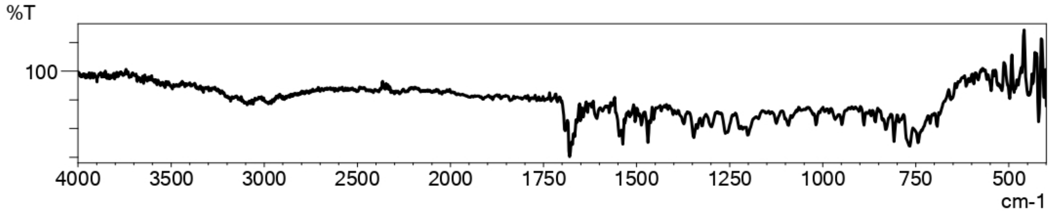

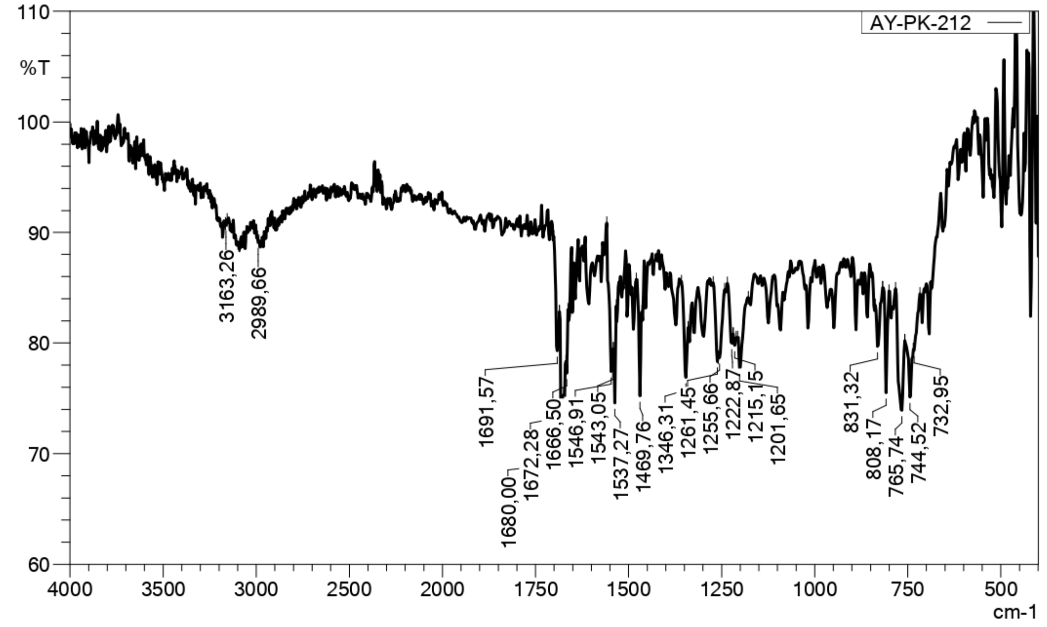

Figure S40. IR spectrum of compound **4n**

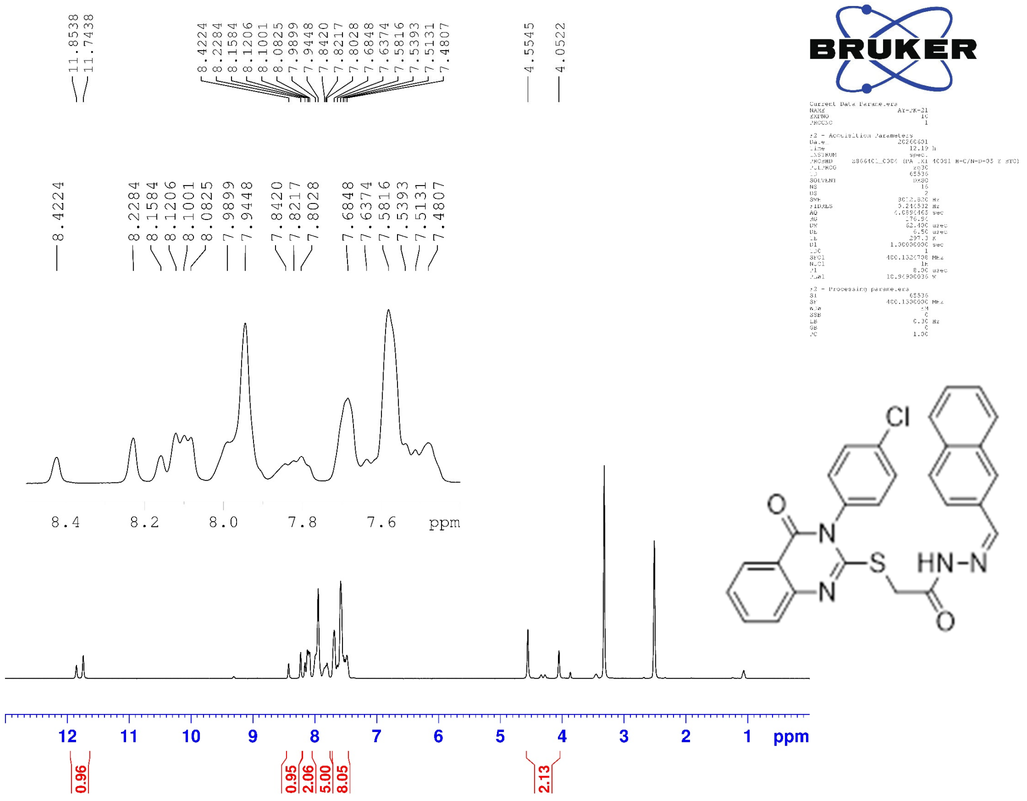

Figure S41. ^{1}H-NMR spectrum of compound **4n**

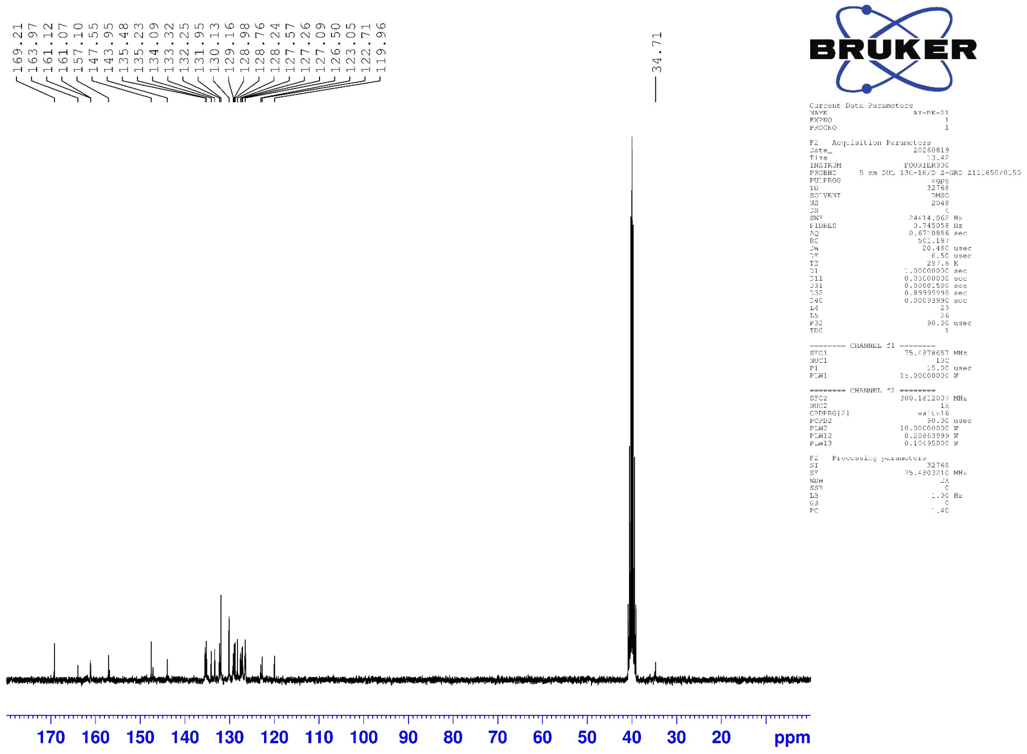

Figure S42. ^{13}C-NMR spectrum of compound **4n**

DOPNALAB

	Item	Value
	Acquired Date&Time	21.08.2026 11:43:12
	Acquired by	System Administrator
	Filename	C:\Users\dopnalab\Desktop\MASAÜSTÜ\FARMASÖTİK\aybuke\AY-PK-241.ispd
	Spectrum name	AY-PK-241
	Sample name	AY-PK-24
	Sample ID	
	Option	
	Comment	
	No. of Scans	30
	Resolution	4 [cm-1]
	Apodization	Happ-Genzel

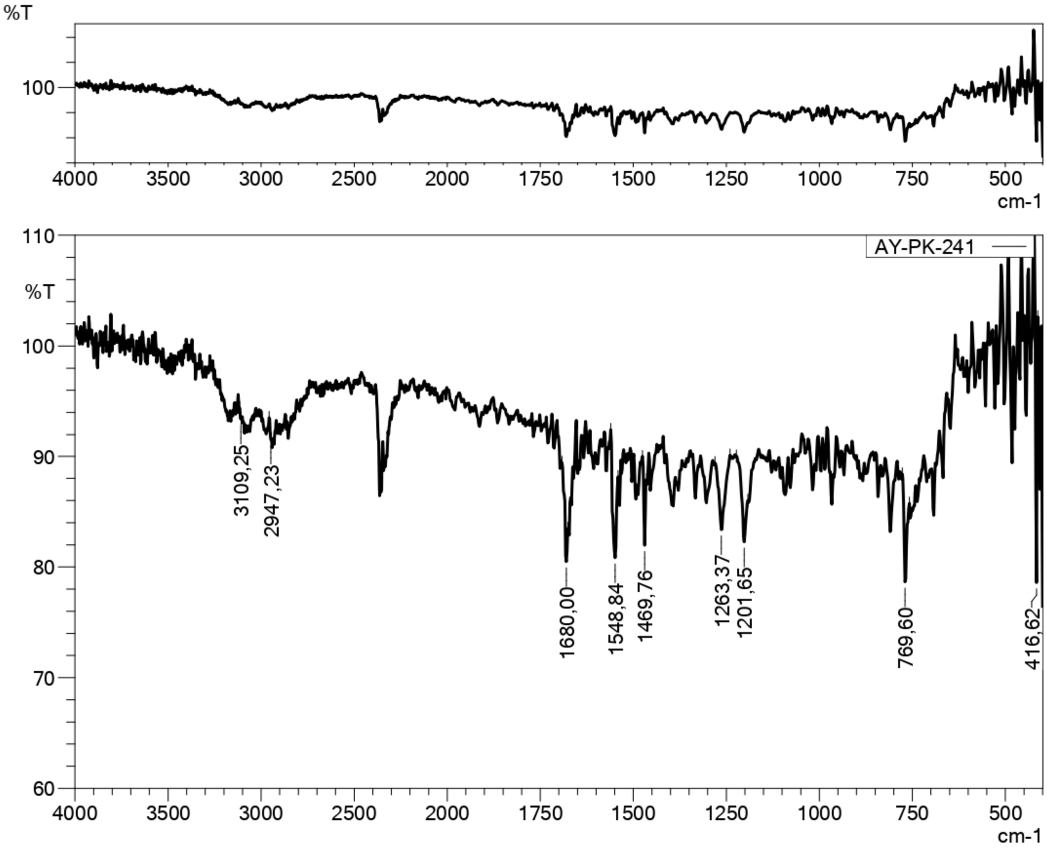

Figure S43. IR spectrum of compound **4o**

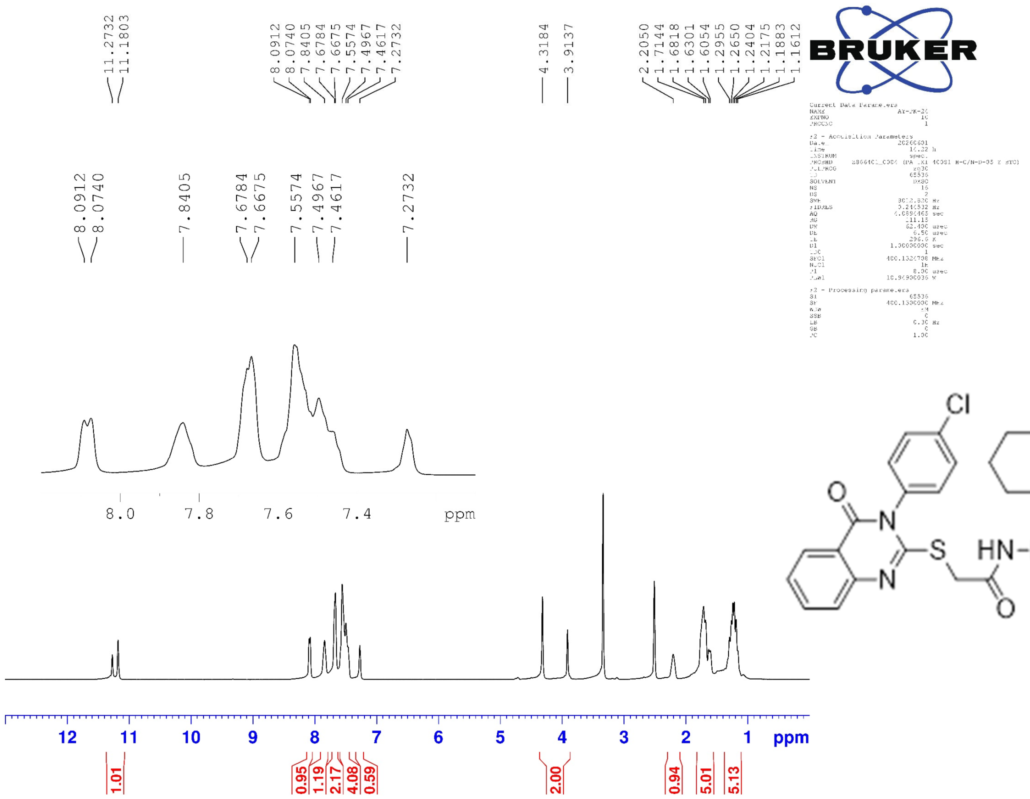

Figure S44. ^{1}H-NMR spectrum of compound **4o**

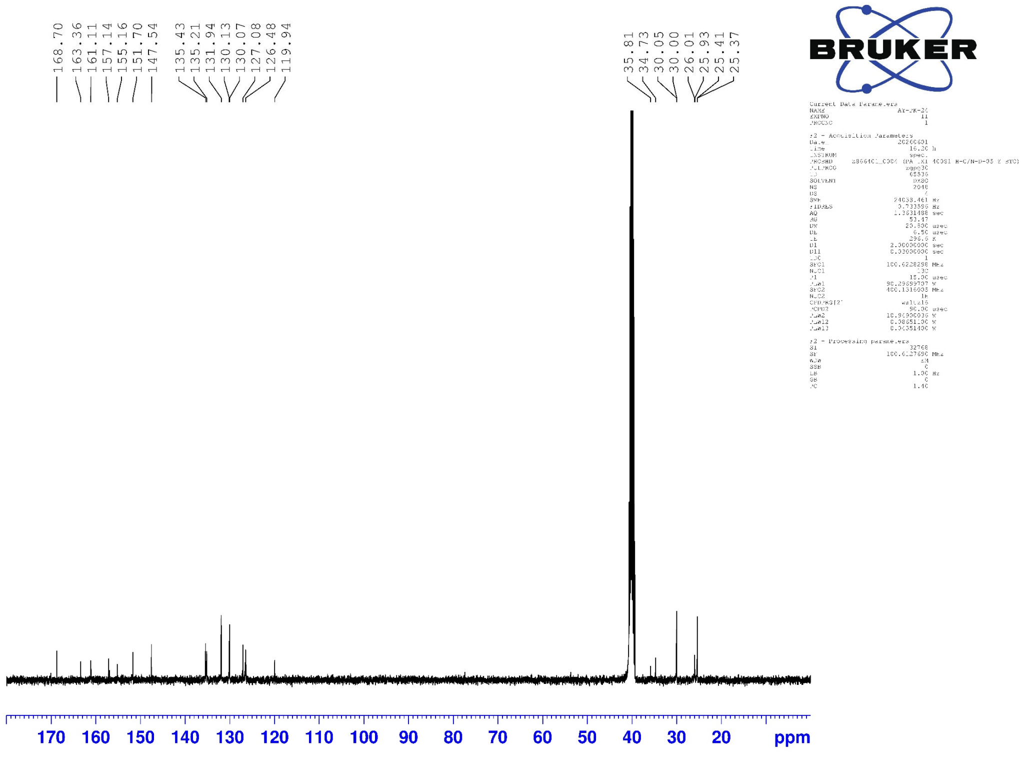

Figure S45. ^{13}C-NMR spectrum of compound **4o**

Data File: C:\LabSolutions\Data\Analiz\Asaf\AY-PK-11_3.lcd

Elmt	Val.	Min	Max	Elmt	Val.	Min	Max	Elmt	Val.	Min	Max	Elmt	Val.	Min	Max	Use Adduct
H	1	8	35	O	2	0	2	S	2	0	2	Ru	2	0	0	H
C	4	9	30	F	1	1	1	Cl	1	0	0	Pd	2	0	0	Na
N	3	5	5	P	3	0	0	Br	1	0	0	I	3	0	0	

Error Margin (ppm):	20	DBE Range:	0.0 - 20.0	Electron Ions:	both
HC Ratio:	unlimited	Apply N Rule:	no	Use MSn Info:	yes
Max Isotopes:	3	Isotope RI (%):	1.00	Isotope Res:	9000
MSn Iso RI (%):	10.00	MSn Logic Mode:	AND	Max Results:	50

Event#: 1 MS(E+) Ret. Time : 3.200 Scan# : 481

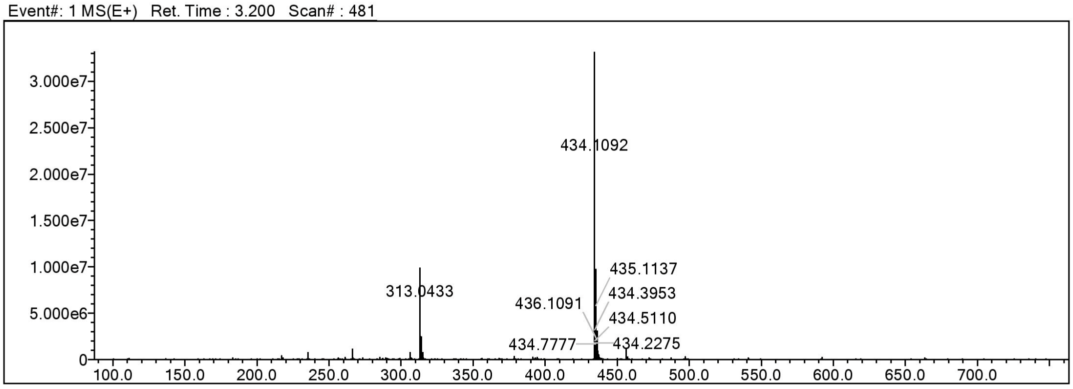

Measured region for 434.1092 m/z

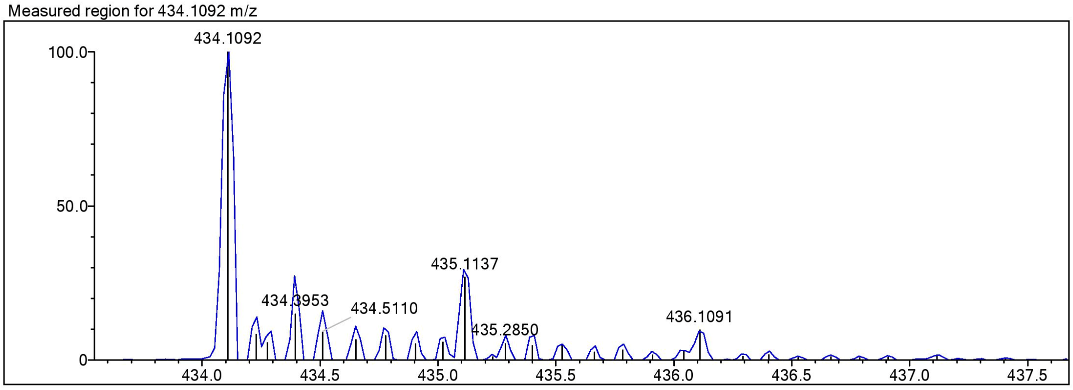

C22 H16 N5 O2 F S [M+H]+ : Predicted region for 434.1082 m/z

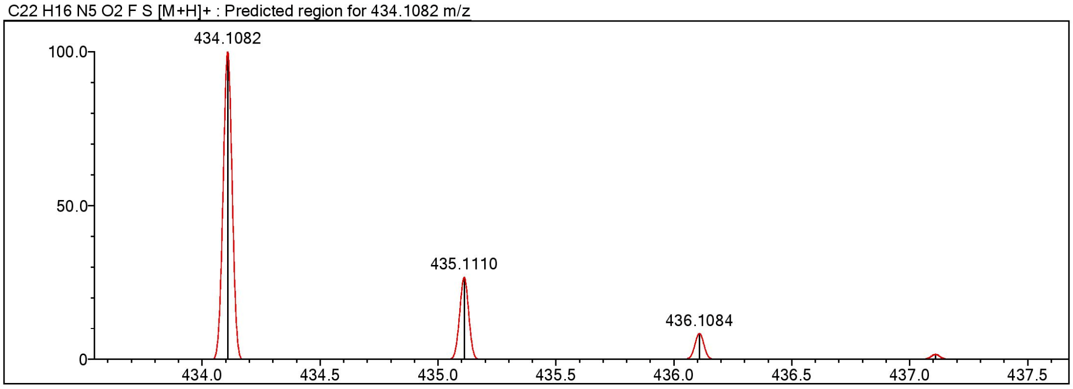

Rank	Score	Formula (M)	Ion	Meas. m/z	Pred. m/z	Df. (mDa)	Df. (ppm)	Iso	DBE
1	77.22	C22 H16 N5 O2 F S	[M+H]+	434.1092	434.1082	1.0	2.30	79.82	17.0

Figure S46. HRMS spectrum of compound **4p**

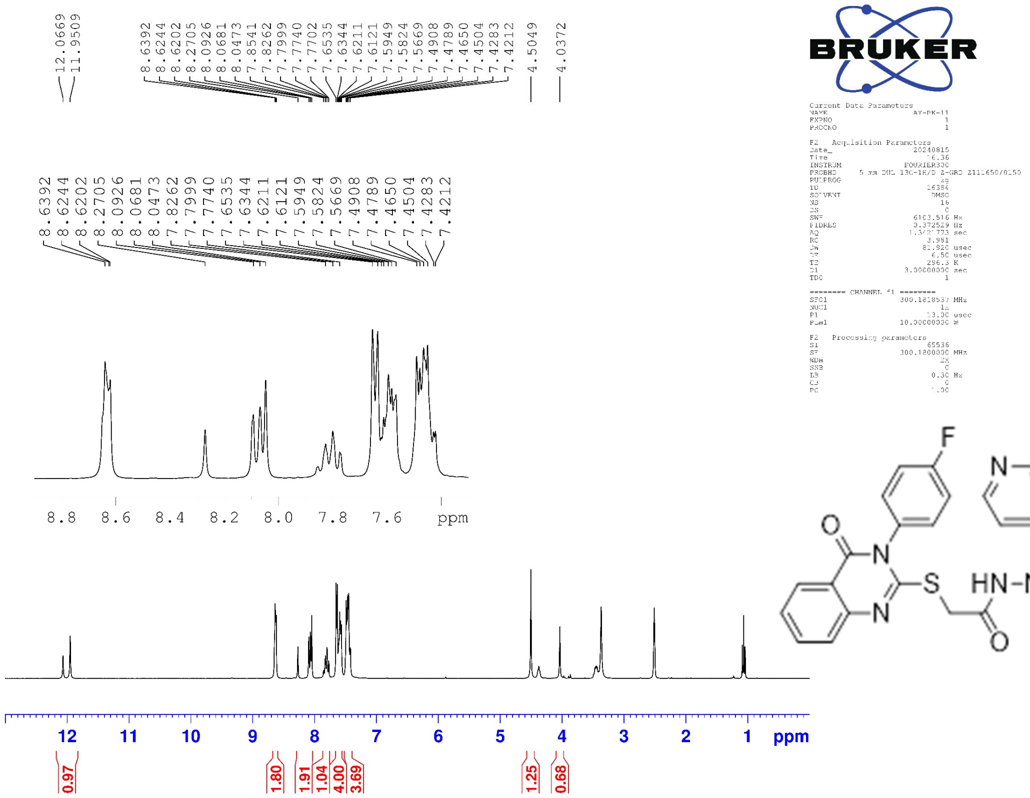

Figure S47. ^{1}H-NMR spectrum of compound **4p**

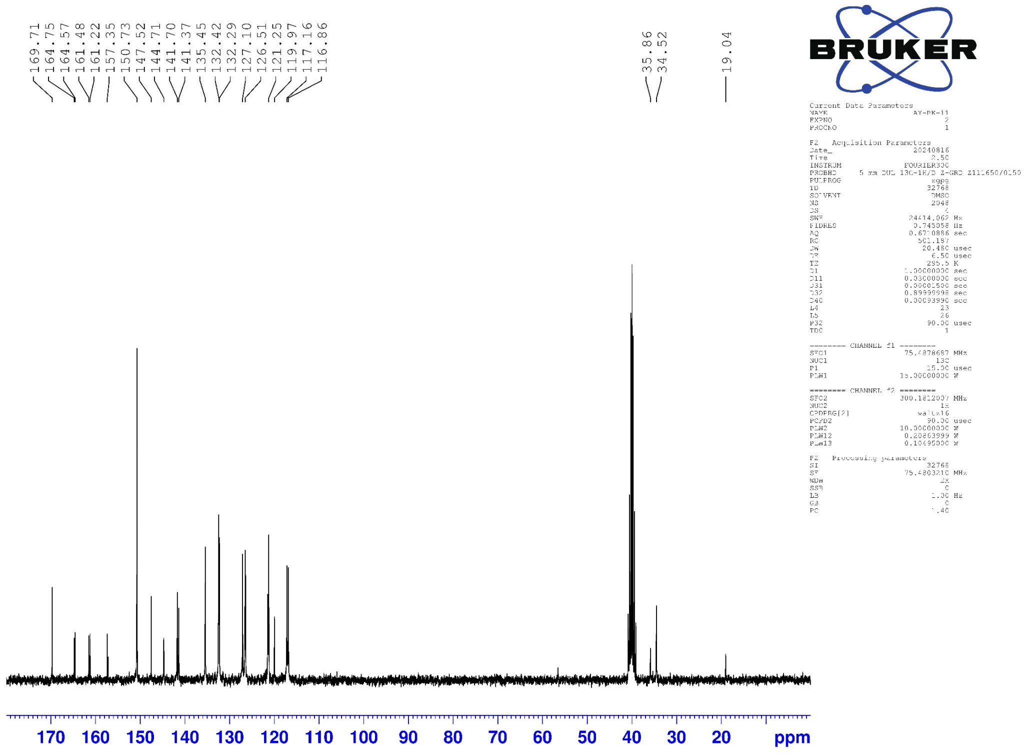

Figure S48. ^{13}C-NMR spectrum of compound **4p**

Data File: C:\LabSolutions\Data\Analiz\Asaf\AY-PK-12_507.lcd

Elmt	Val.	Min	Max	Elmt	Val.	Min	Max	Elmt	Val.	Min	Max	Elmt	Val.	Min	Max	Use Adduct
H	1	10	33	O	2	0	2	S	2	0	2	Ru	2	0	0	H
C	4	4	32	F	1	1	1	Cl	1	0	0	Pd	2	0	0	Na
N	3	0	5	P	3	0	0	Br	1	0	0	I	3	0	0	

Error Margin (ppm):	5	DBE Range:	0.0 - 30.0	Electron Ions:	both
HC Ratio:	unlimited	Apply N Rule:	no	Use MSn Info:	yes
Max Isotopes:	3	Isotope RI (%):	1.00	Isotope Res:	9000
MSn Iso RI (%):	10.00	MSn Logic Mode:	AND	Max Results:	50

Event#: 1 MS(E+) Ret. Time : 5.213 Scan# : 783

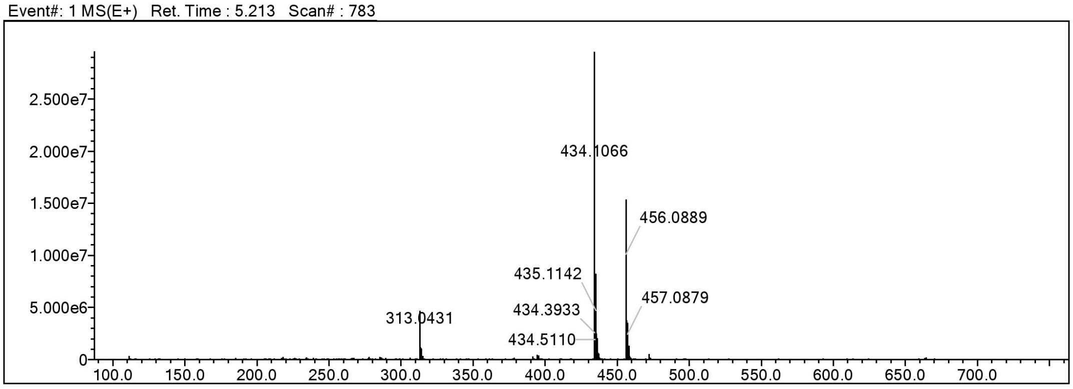

Measured region for 434.1066 m/z

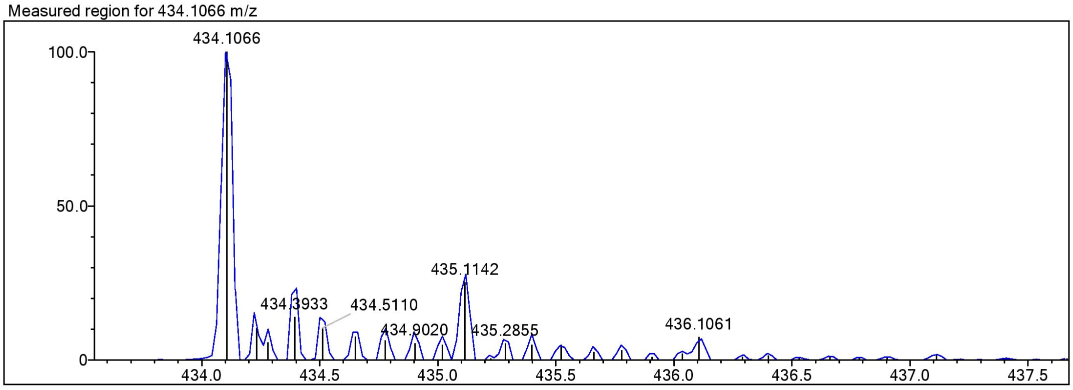

C22 H16 N5 O2 F S [M+H]+ : Predicted region for 434.1082 m/z

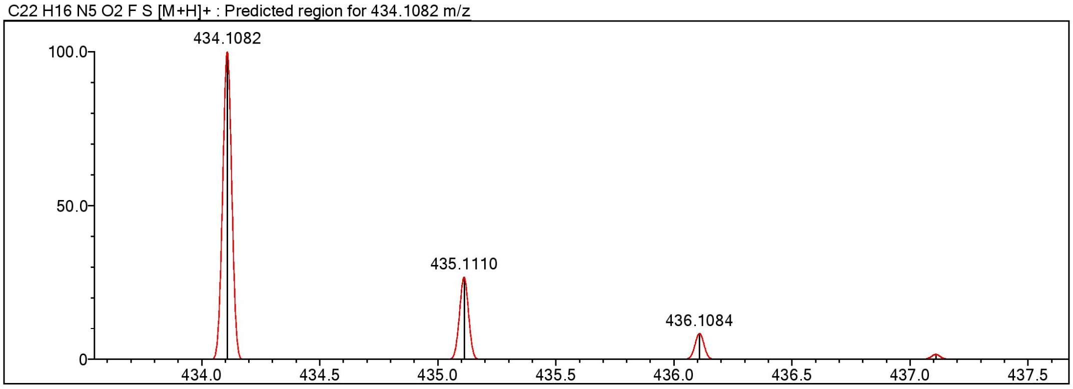

Rank	Score	Formula (M)	Ion	Meas. m/z	Pred. m/z	Df. (mDa)	Df. (ppm)	Iso	DBE
1	89.37	C22 H16 N5 O2 F S	[M+H]+	434.1066	434.1082	-1.6	-3.69	95.81	17.0

Figure S49. HRMS spectrum of compound **4q**

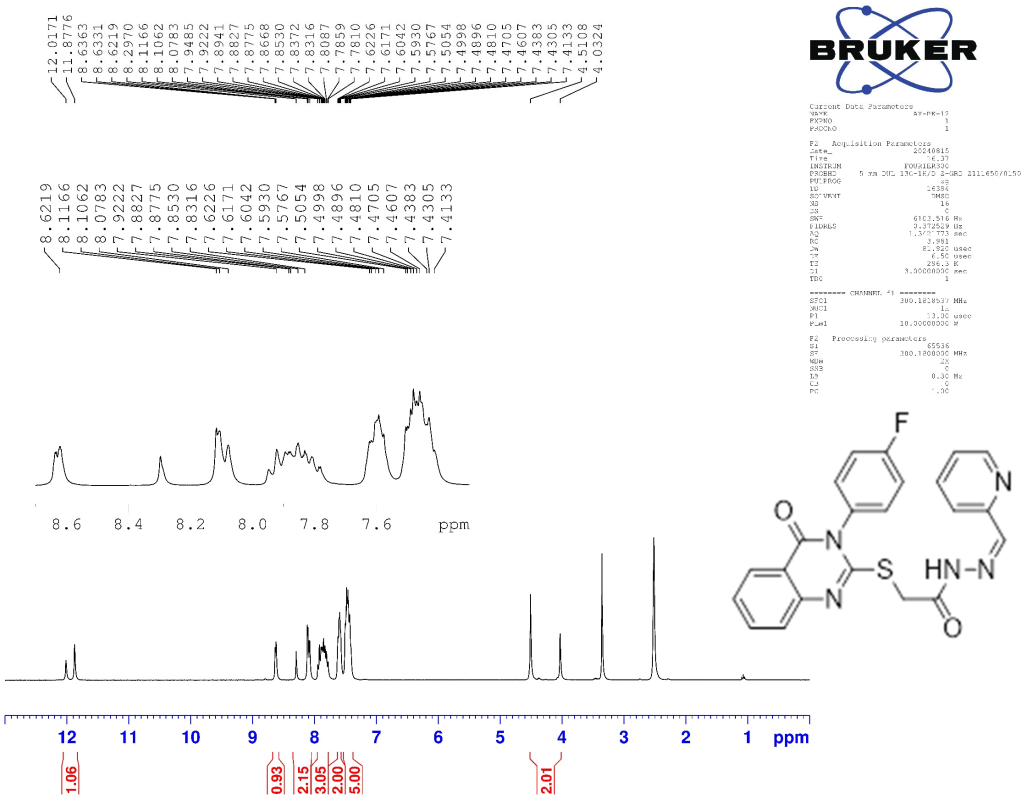

Figure S50. ^{1}H-NMR spectrum of compound **4q**

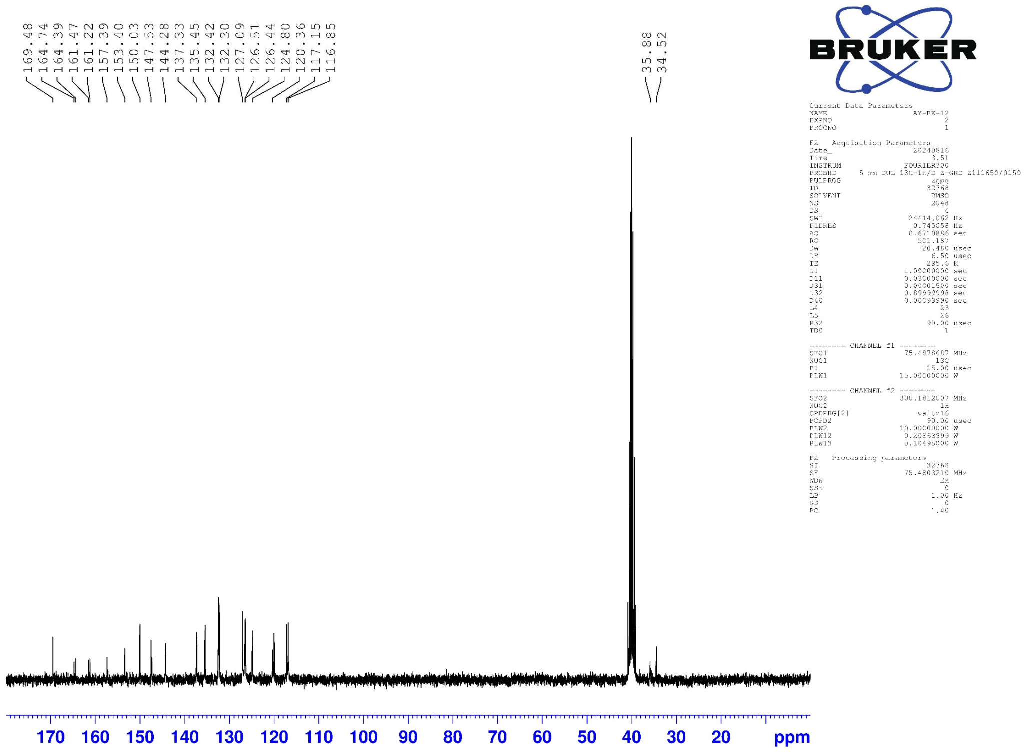

Figure S51. ^{13}C-NMR spectrum of compound **4q**

Data File: C:\LabSolutions\Data\Analiz\Asaf\AY-PK-13_4.lcd

Elmt	Val.	Min	Max	Elmt	Val.	Min	Max	Elmt	Val.	Min	Max	Elmt	Val.	Min	Max	Use Adduct
H	1	8	35	O	2	0	2	S	2	0	2	Ru	2	0	0	H
C	4	9	30	F	1	1	1	Cl	1	0	0	Pd	2	0	0	Na
N	3	0	5	P	3	0	0	Br	1	0	0	I	3	0	0	

Error Margin (ppm):	20	DBE Range:	0.0 - 20.0	Electron Ions:	both
HC Ratio:	unlimited	Apply N Rule:	no	Use MSn Info:	yes
Max Isotopes:	3	Isotope RI (%):	1.00	Isotope Res:	9000
MSn Iso RI (%):	10.00	MSn Logic Mode:	AND	Max Results:	50

Event#: 1 MS(E+) Ret. Time : 4.960 Scan# : 745

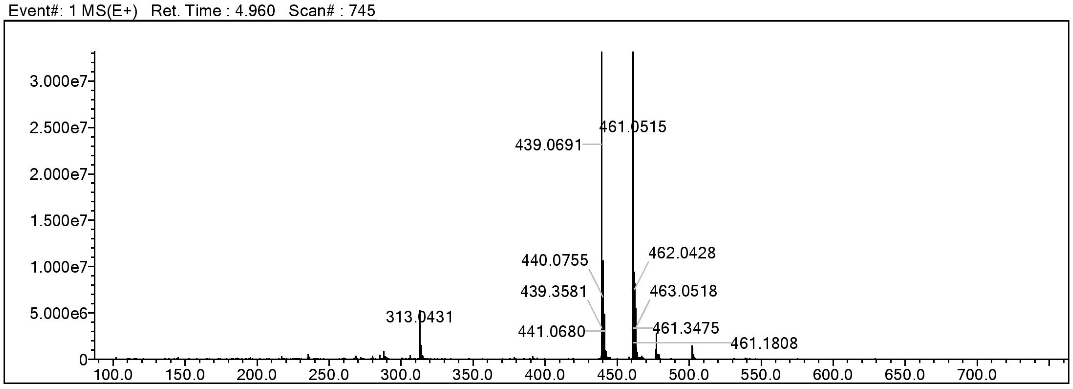

Measured region for 439.0691 m/z

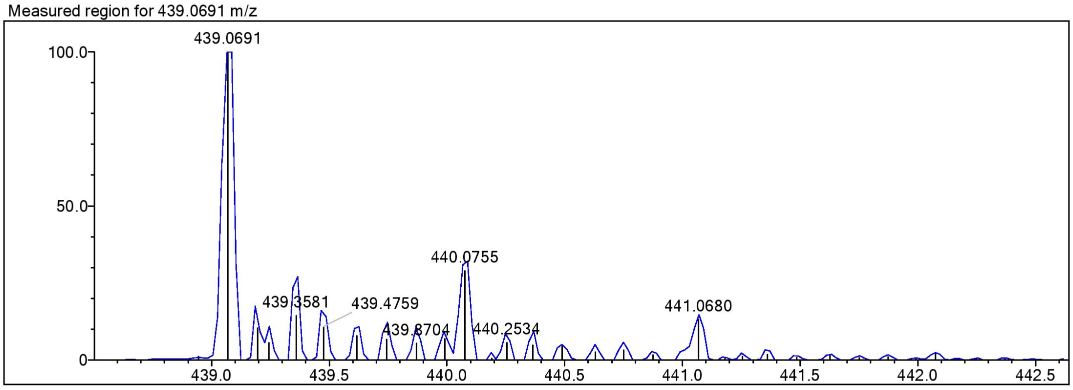

C21 H15 N4 O2 F S2 [M+H]+ : Predicted region for 439.0693 m/z

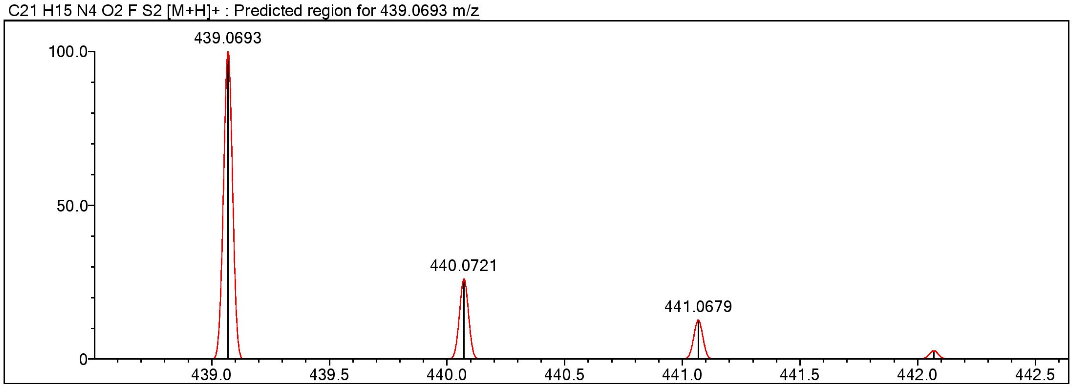

Rank	Score	Formula (M)	Ion	Meas. m/z	Pred. m/z	Df. (mDa)	Df. (ppm)	Iso	DBE
2	74.14	C21 H15 N4 O2 F S2	[M+H]+	439.0691	439.0693	-0.2	-0.46	74.14	16.0

Figure S52. HRMS spectrum of compound **4r**

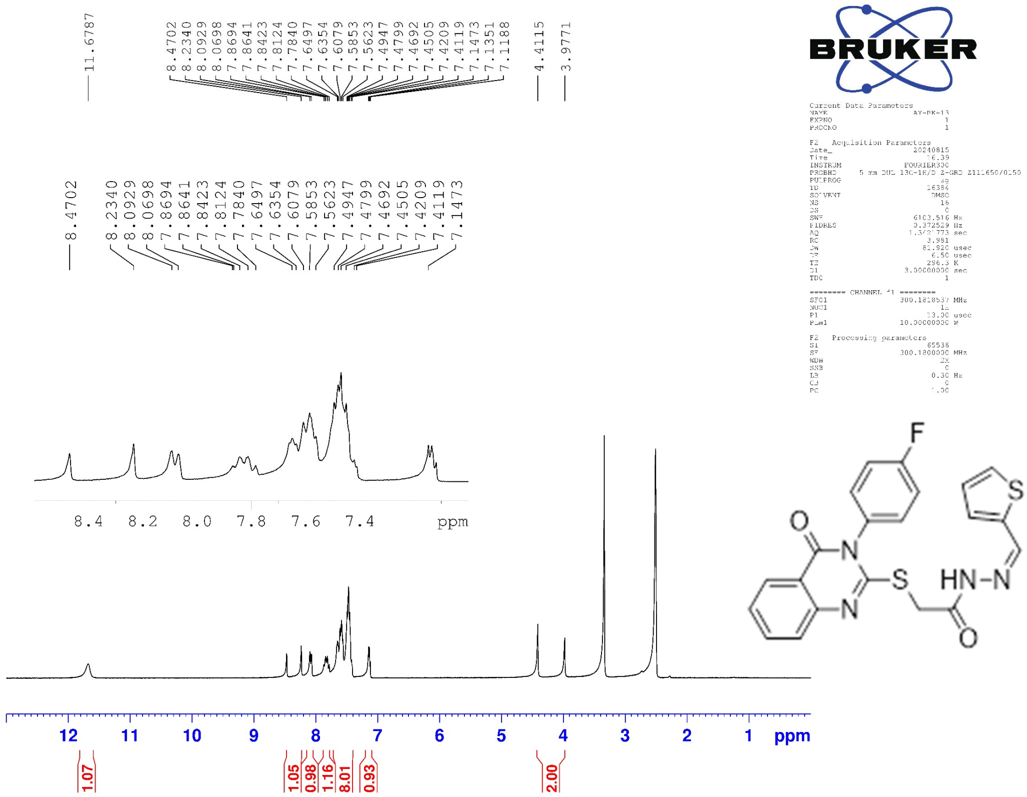

Figure S53. ^{1}H-NMR spectrum of compound **4r**

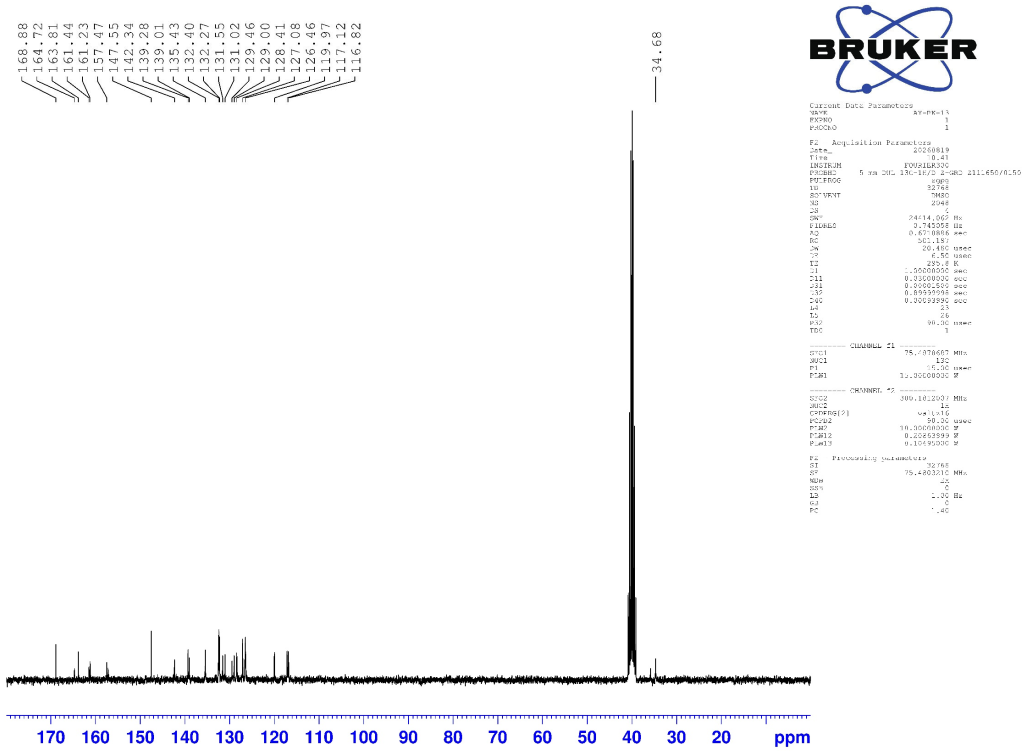

Figure S54. ^{13}C-NMR spectrum of compound **4r**

Data File: C:\LabSolutions\Data\Analiz\Asaf\AY-PK-14_5.lcd

Elmt	Val.	Min	Max	Elmt	Val.	Min	Max	Elmt	Val.	Min	Max	Elmt	Val.	Min	Max	Use Adduct
H	1	8	35	O	2	0	2	S	2	0	2	Ru	2	0	0	H
C	4	9	30	F	1	1	1	Cl	1	0	0	Pd	2	0	0	Na
N	3	0	5	P	3	0	0	Br	1	0	0	I	3	0	0	

Error Margin (ppm):	20	DBE Range:	0.0 - 20.0	Electron Ions:	both
HC Ratio:	unlimited	Apply N Rule:	no	Use MSn Info:	yes
Max Isotopes:	3	Isotope RI (%):	1.00	Isotope Res:	9000
MSn Iso RI (%):	10.00	MSn Logic Mode:	AND	Max Results:	50

Event#: 1 MS(E+) Ret. Time : 4.173 Scan# : 627

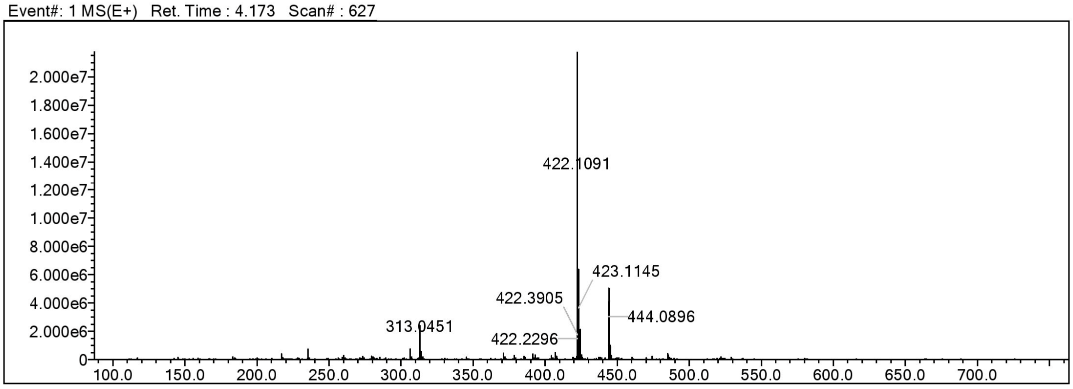

Measured region for 422.1091 m/z

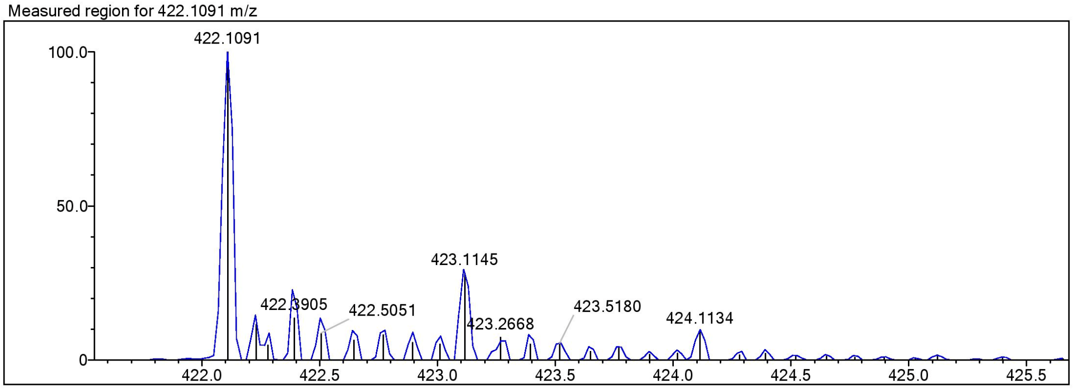

C21 H16 N5 O2 F S [M+H]+ : Predicted region for 422.1082 m/z

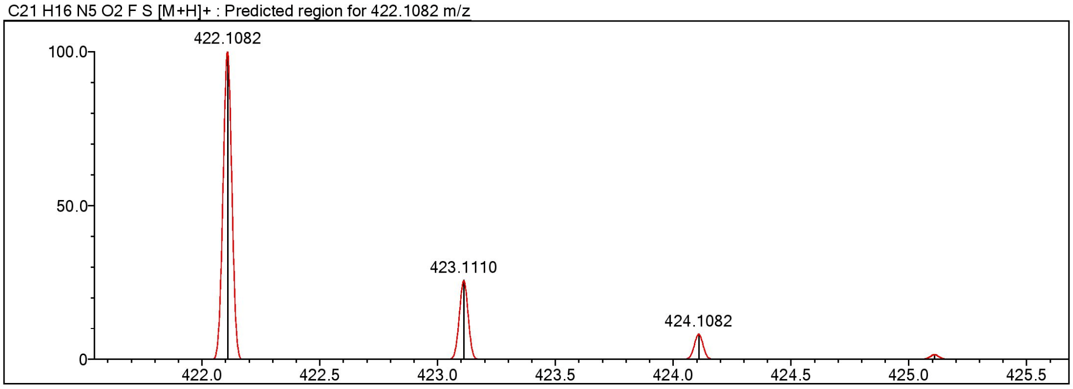

Rank	Score	Formula (M)	Ion	Meas. m/z	Pred. m/z	Df. (mDa)	Df. (ppm)	Iso	DBE
3	74.62	C21 H16 N5 O2 F S	[M+H]+	422.1091	422.1082	0.9	2.13	76.79	16.0

Figure S55. HRMS spectrum of compound **4s**

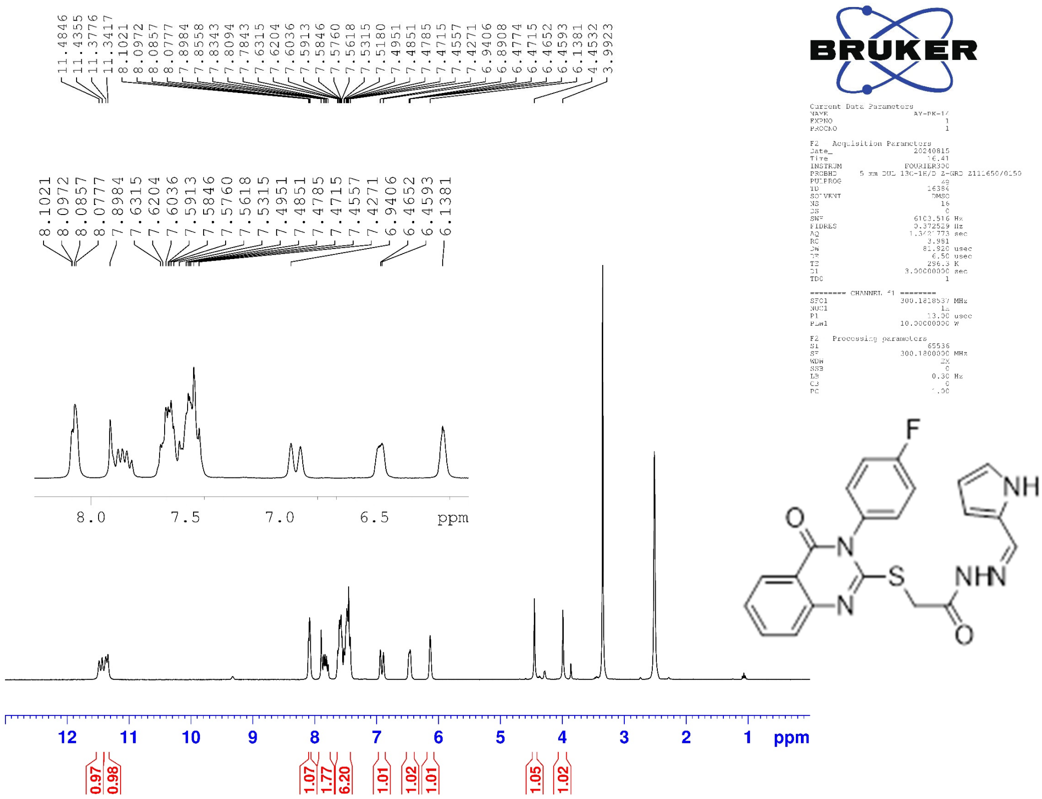

Figure S56. ^{1}H-NMR spectrum of compound **4s**

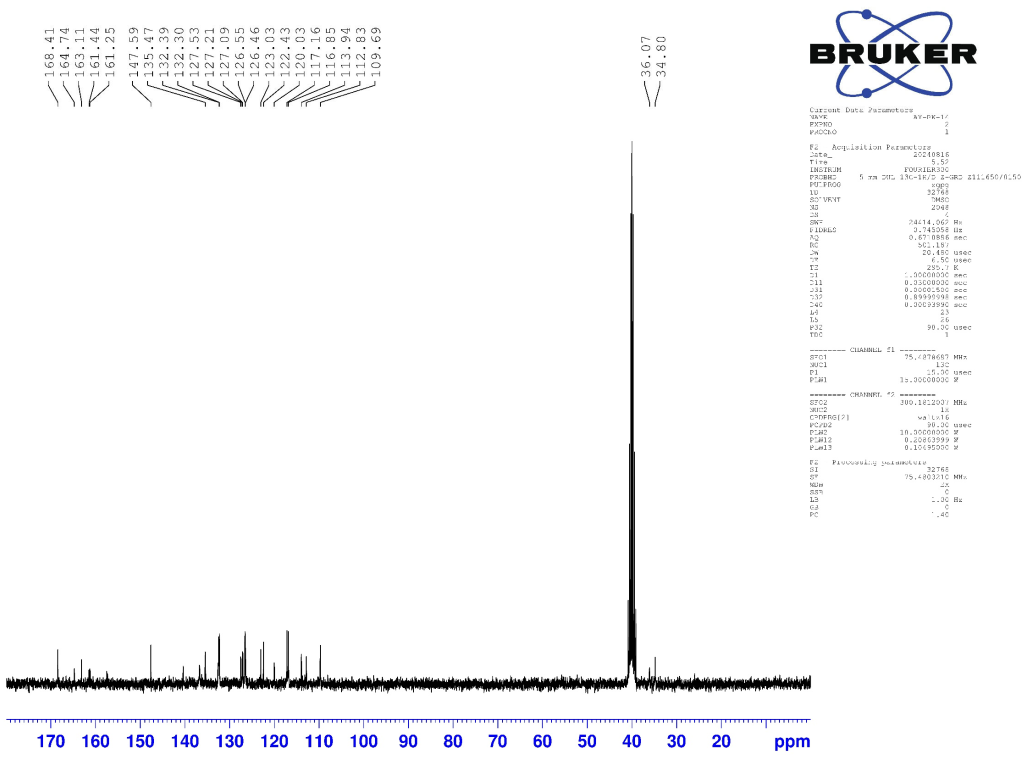

Figure S57. ^{13}C-NMR spectrum of compound **4s**

Data File: C:\LabSolutions\Data\Analiz\Asaf\AY-PK-18_526.lcd

Elmt	Val.	Min	Max	Elmt	Val.	Min	Max	Elmt	Val.	Min	Max	Elmt	Val.	Min	Max	Use Adduct
H	1	8	35	O	2	0	3	S	2	0	2	Ru	2	0	0	H
C	4	9	30	F	1	1	1	Cl	1	0	0	Pd	2	0	0	Na
N	3	0	5	P	3	0	0	Br	1	0	0	I	3	0	0	

Error Margin (ppm): 5
HC Ratio: unlimited
Max Isotopes: 3
MSn Iso RI (%): 10.00

DBE Range: 0.0 - 20.0
Apply N Rule: no
Isotope RI (%): 1.00
MSn Logic Mode: AND

Electron Ions: both
Use MSn Info: yes
Isotope Res: 9000
Max Results: 50

Event#: 1 MS(E+) Ret. Time : 4.467 Scan# : 671

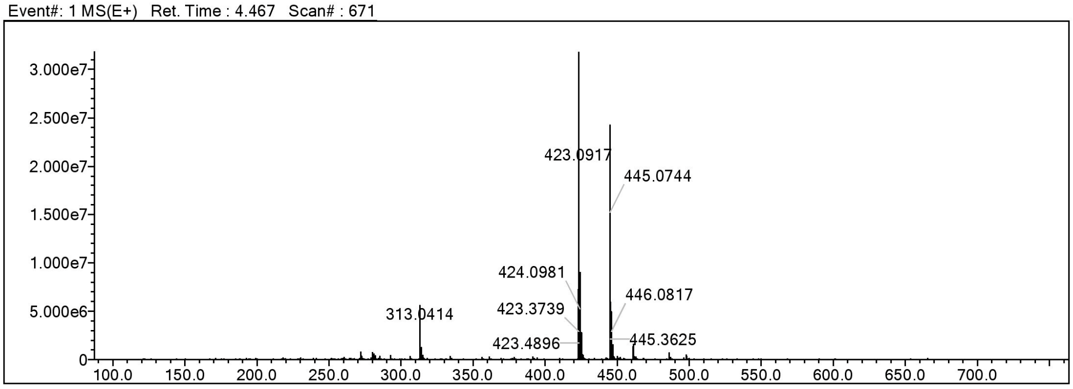

Measured region for 423.0917 m/z

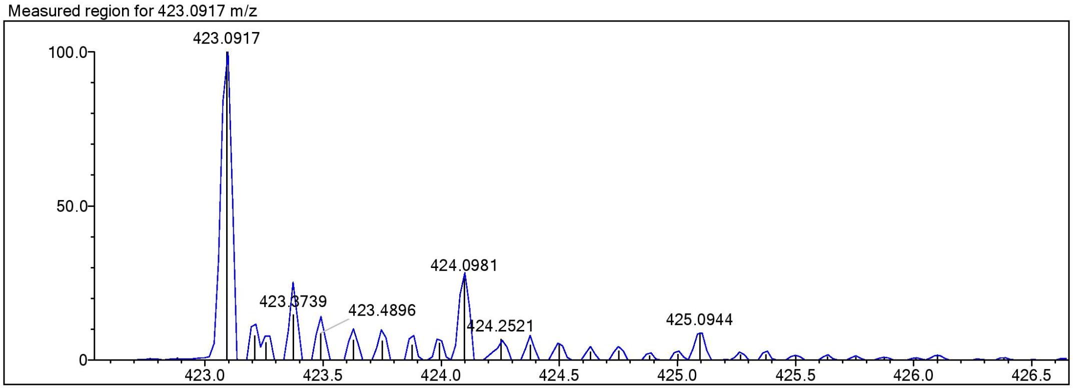

C21 H15 N4 O3 F S [M+H]+ : Predicted region for 423.0922 m/z

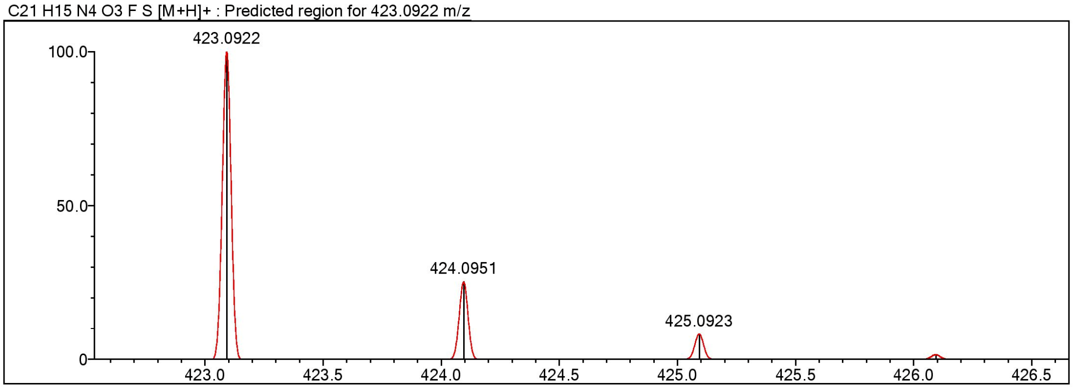

Rank	Score	Formula (M)	Ion	Meas. m/z	Pred. m/z	Df. (mDa)	Df. (ppm)	Iso	DBE
1	87.70	C21 H15 N4 O3 F S	[M+H]+	423.0917	423.0922	-0.5	-1.18	88.09	16.0

Figure S58. HRMS spectrum of compound **4t**

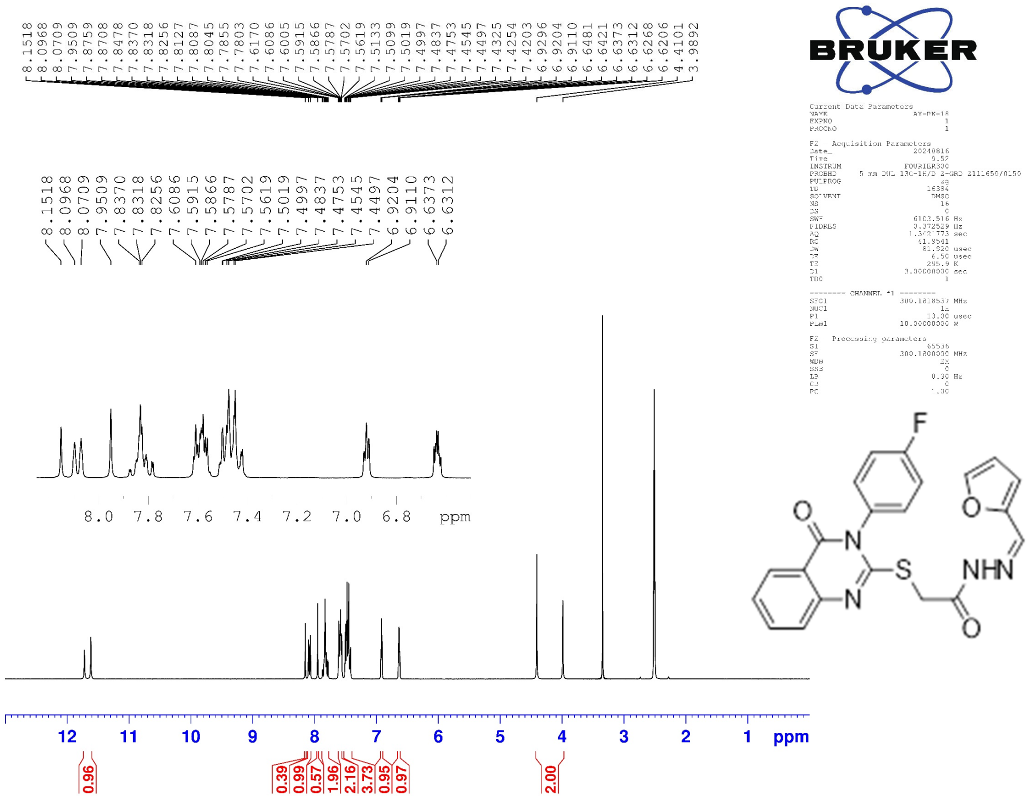

Figure S59. ^{1}H-NMR spectrum of compound **4t**

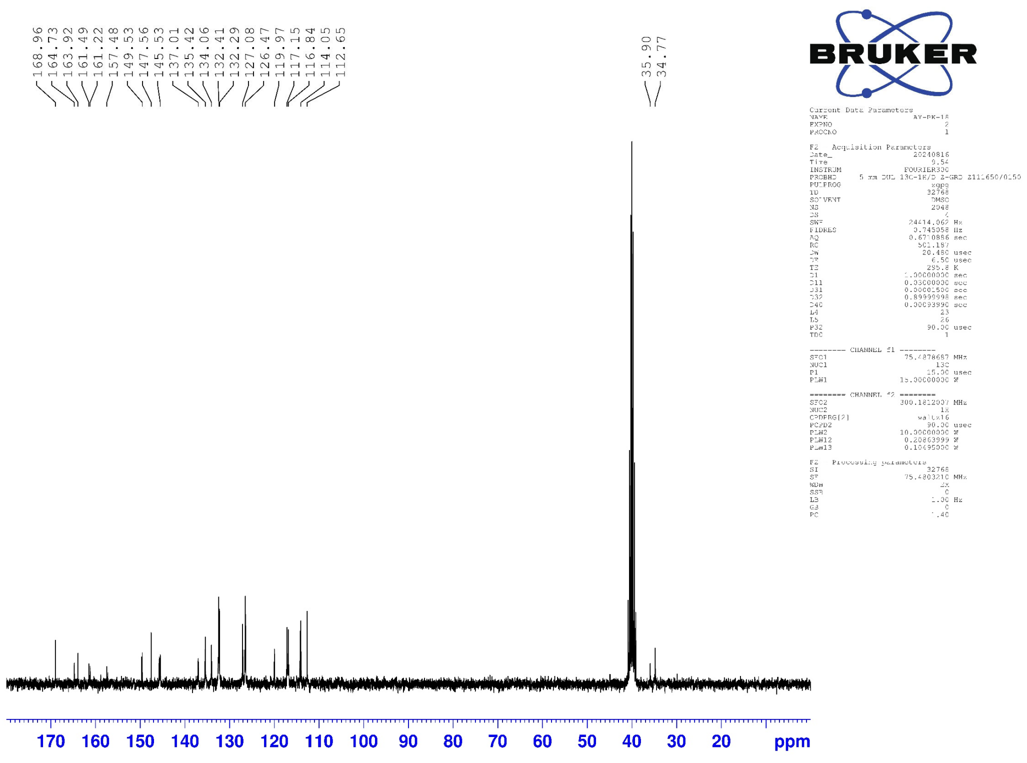

Figure S60. ^{13}C-NMR spectrum of compound **4t**

Data File: C:\LabSolutions\Data\Analiz\Asaf\AY-PK-17_525.lcd

Elmt	Val.	Min	Max	Elmt	Val.	Min	Max	Elmt	Val.	Min	Max	Elmt	Val.	Min	Max	Use Adduct
H	1	8	35	O	2	0	3	S	2	0	2	Ru	2	0	0	H
C	4	9	30	F	1	1	1	Cl	1	0	0	Pd	2	0	0	Na
N	3	0	5	P	3	0	0	Br	1	0	0	I	3	0	0	

Error Margin (ppm):	5	DBE Range:	0.0 - 20.0	Electron Ions:	both
HC Ratio:	unlimited	Apply N Rule:	no	Use MSn Info:	yes
Max Isotopes:	3	Isotope RI (%):	1.00	Isotope Res:	9000
MSn Iso RI (%):	10.00	MSn Logic Mode:	AND	Max Results:	50

Event#: 1 MS(E+) Ret. Time : 4.387 -> 5.147 - 8.520 -> 9.853 Scan# : 659 -> 773 - 1279 -> 1479

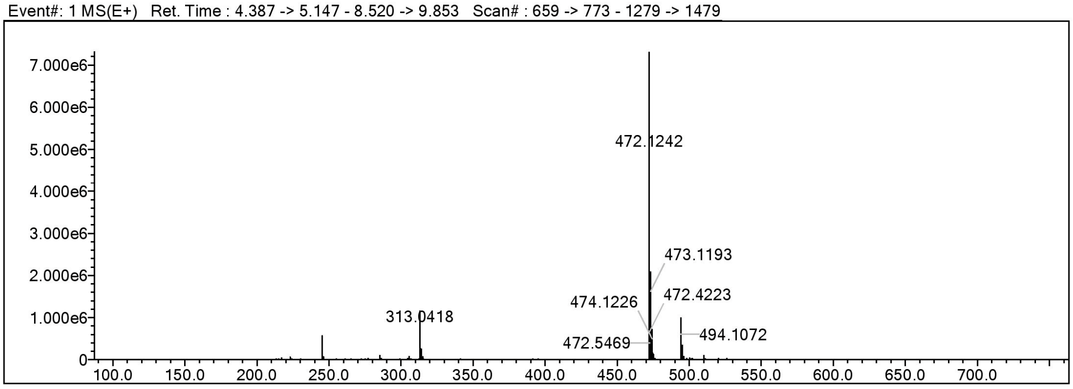

Measured region for 472.1242 m/z

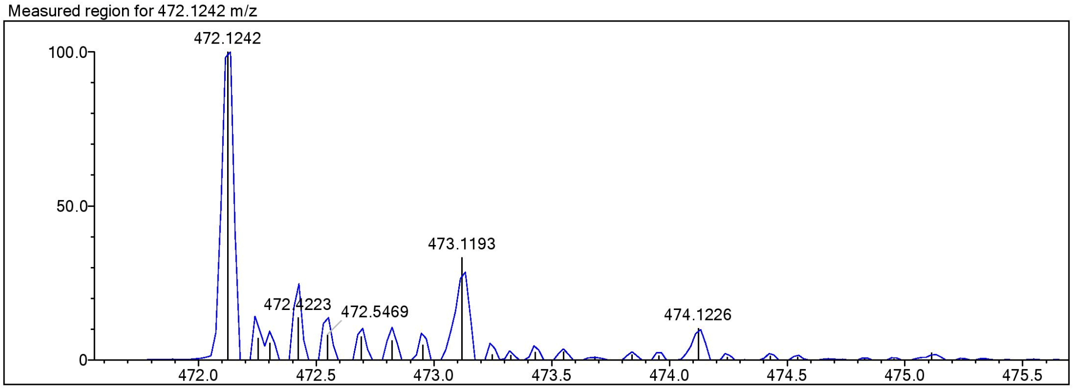

C25 H18 N5 O2 F S [M+H]+ : Predicted region for 472.1238 m/z

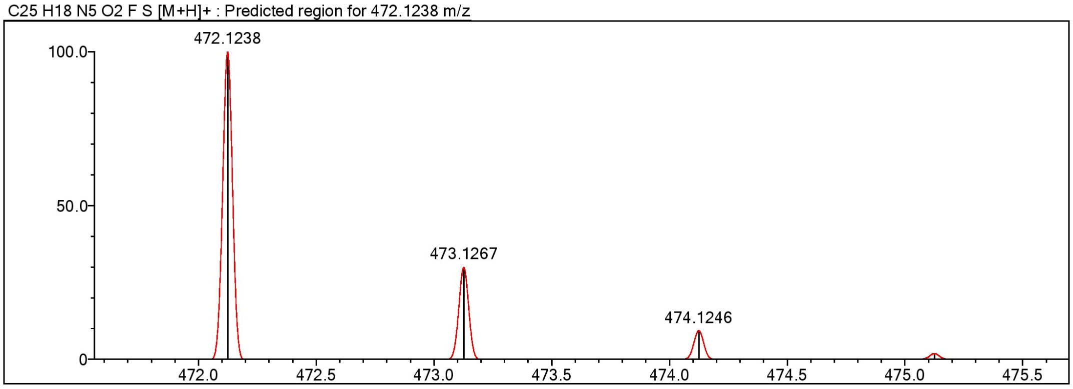

Rank	Score	Formula (M)	Ion	Meas. m/z	Pred. m/z	Df. (mDa)	Df. (ppm)	Iso	DBE
1	79.29	C25 H18 N5 O2 F S	[M+H]+	472.1242	472.1238	0.4	0.85	79.29	19.0

Figure S61. HRMS spectrum of compound **4u**

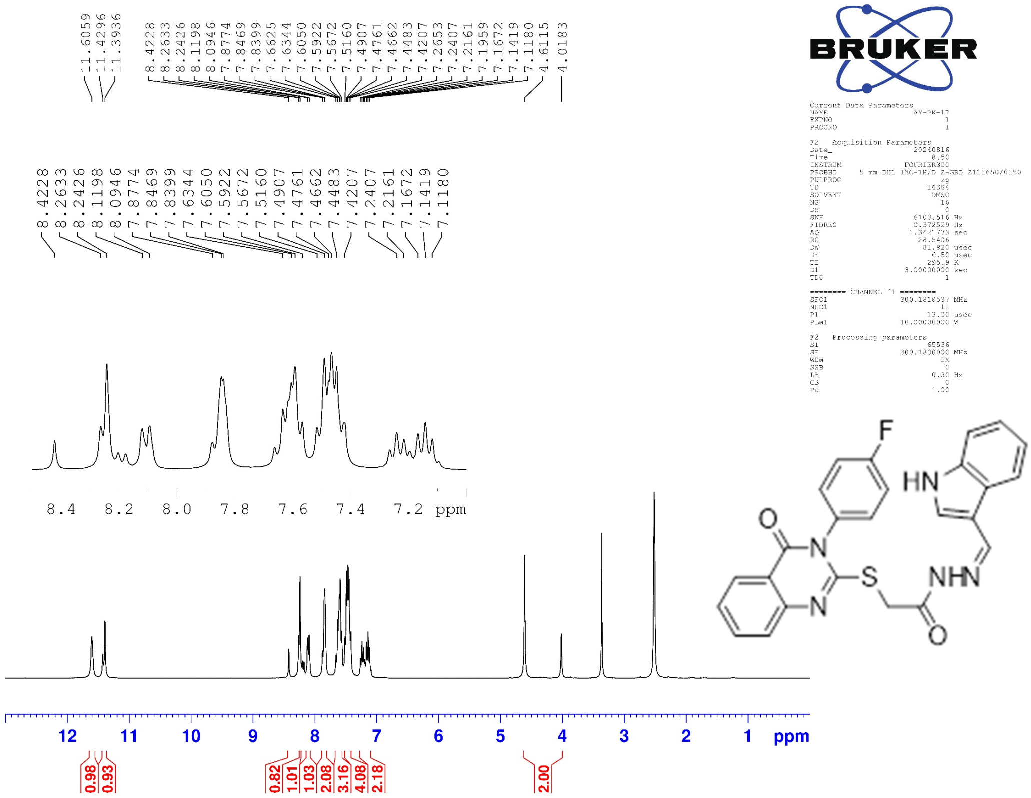

Figure S62. ^{1}H-NMR spectrum of compound **4u**

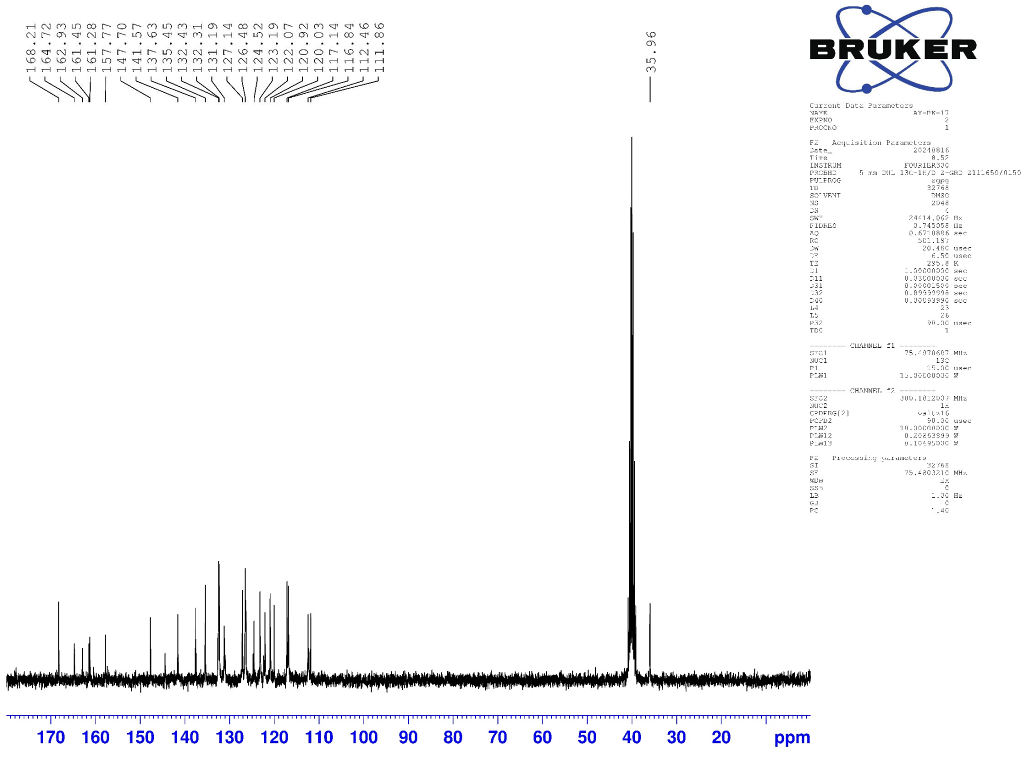

Figure S63. ^{13}C-NMR spectrum of compound **4u**

Data File: C:\LabSolutions\Data\Analiz\Asaf\AY-PK-15_523.lcd

Elmt	Val.	Min	Max	Elmt	Val.	Min	Max	Elmt	Val.	Min	Max	Elmt	Val.	Min	Max	Use Adduct
H	1	8	35	O	2	0	3	S	2	0	2	Ru	2	0	0	H
C	4	9	30	F	1	1	1	Cl	1	0	0	Pd	2	0	0	Na
N	3	0	5	P	3	0	0	Br	1	0	0	I	3	0	0	

Error Margin (ppm):	5	DBE Range:	0.0 - 20.0	Electron Ions:	both
HC Ratio:	unlimited	Apply N Rule:	no	Use MSn Info:	yes
Max Isotopes:	3	Isotope RI (%):	1.00	Isotope Res:	9000
MSn Iso RI (%):	10.00	MSn Logic Mode:	AND	Max Results:	50

Event#: 1 MS(E+) Ret. Time : 7.413 Scan# : 1113

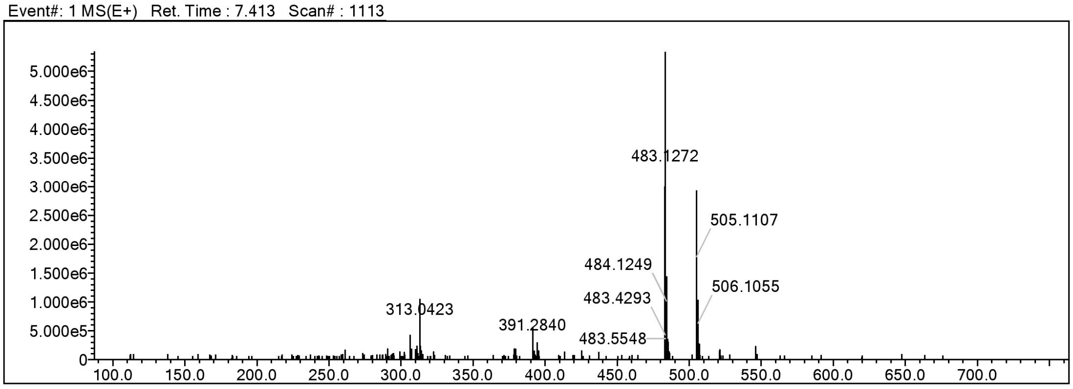

Measured region for 483.1272 m/z

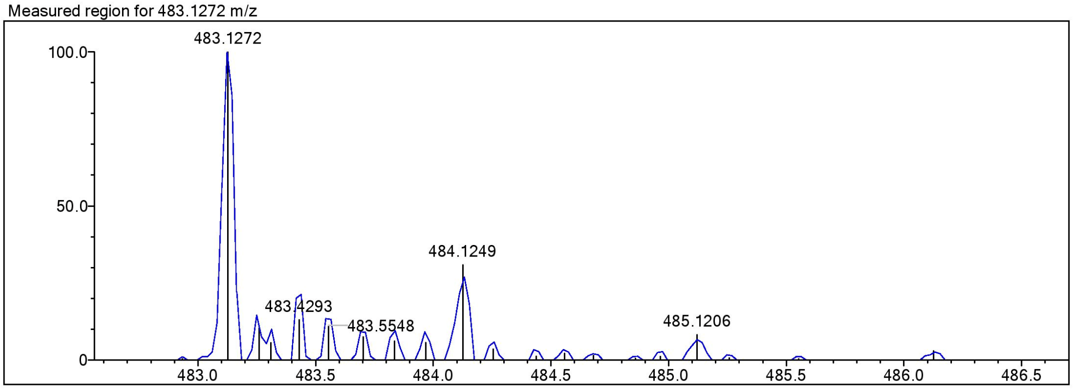

C27 H19 N4 O2 F S [M+H]+ : Predicted region for 483.1286 m/z

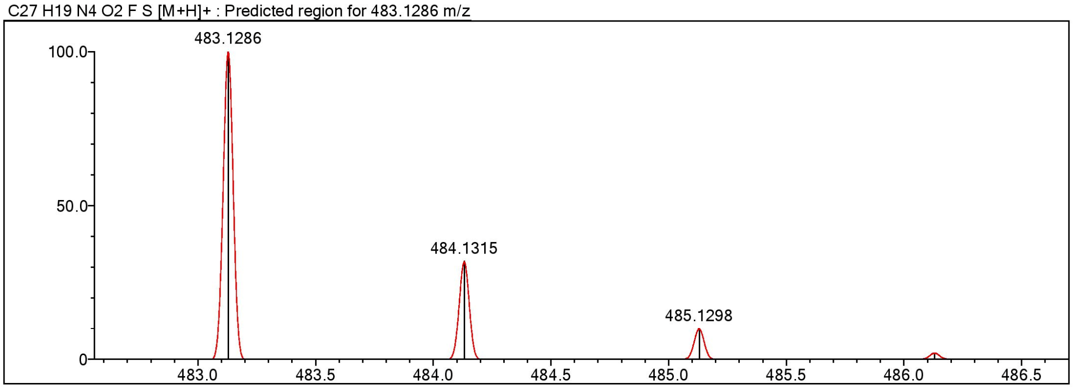

Rank	Score	Formula (M)	Ion	Meas. m/z	Pred. m/z	Df. (mDa)	Df. (ppm)	Iso	DBE
3	67.05	C27 H19 N4 O2 F S	[M+H]+	483.1272	483.1286	-1.4	-2.90	70.39	20.0

Figure S64. HRMS spectrum of compound **4v**

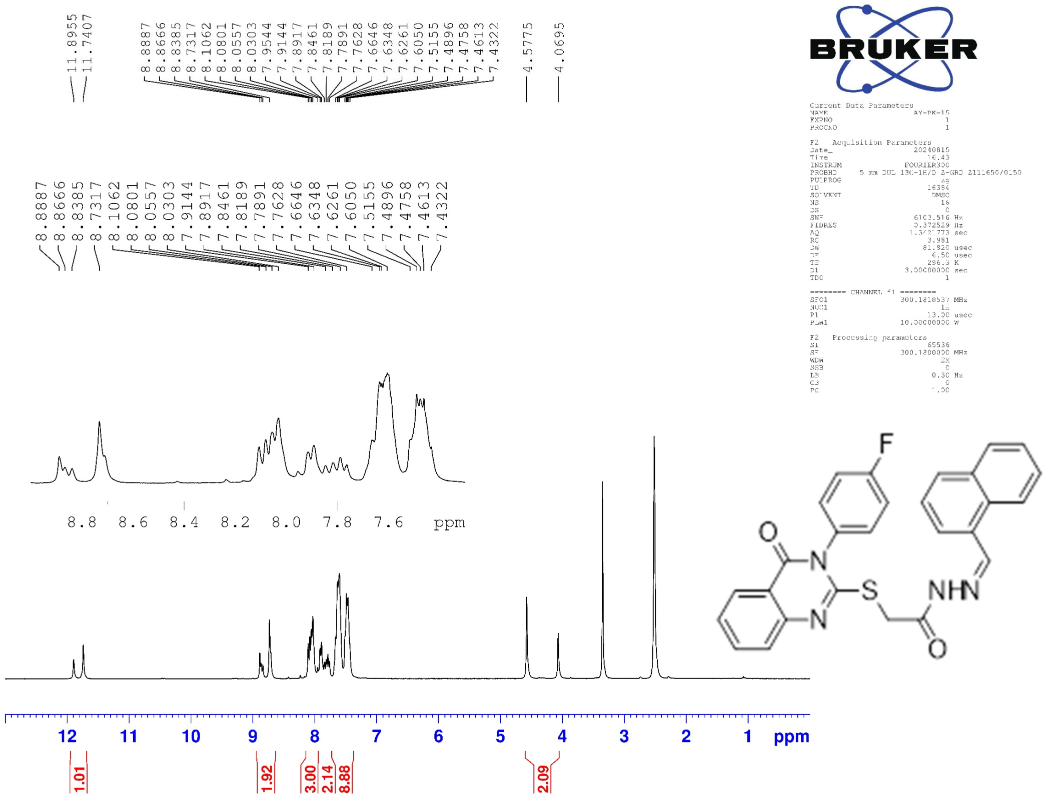

Figure S65. ^{1}H-NMR spectrum of compound **4v**

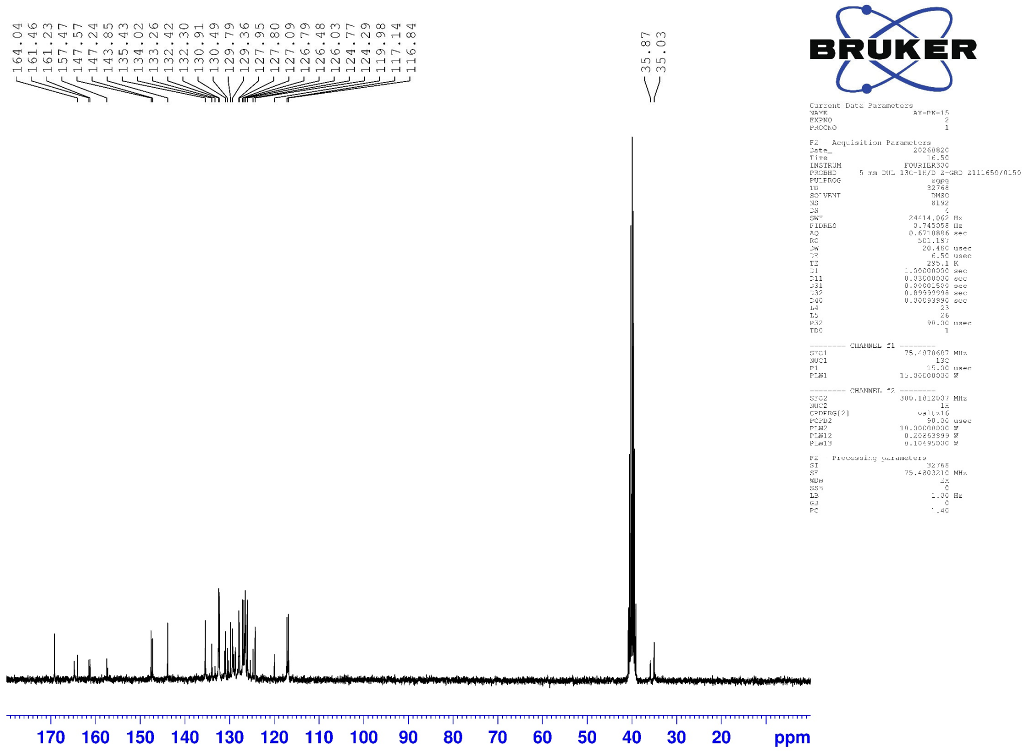

Figure S66. ^{13}C-NMR spectrum of compound **4v**

Data File: C:\LabSolutions\Data\Analiz\Asaf\AY-PK-16_524.lcd

Elmt	Val.	Min	Max	Elmt	Val.	Min	Max	Elmt	Val.	Min	Max	Elmt	Val.	Min	Max	Use Adduct
H	1	8	35	O	2	0	3	S	2	0	2	Ru	2	0	0	H
C	4	9	30	F	1	1	1	Cl	1	0	0	Pd	2	0	0	Na
N	3	0	5	P	3	0	0	Br	1	0	0	I	3	0	0	

Error Margin (ppm):	5	DBE Range:	0.0 - 20.0	Electron Ions:	both
HC Ratio:	unlimited	Apply N Rule:	no	Use MSn Info:	yes
Max Isotopes:	3	Isotope RI (%):	1.00	Isotope Res:	9000
MSn Iso RI (%):	10.00	MSn Logic Mode:	AND	Max Results:	50

Event#: 1 MS(E+) Ret. Time : 6.920 -> 7.627 - 1.853 -> 2.541 Scan# : 1039 -> 1145 - 279 -> 383

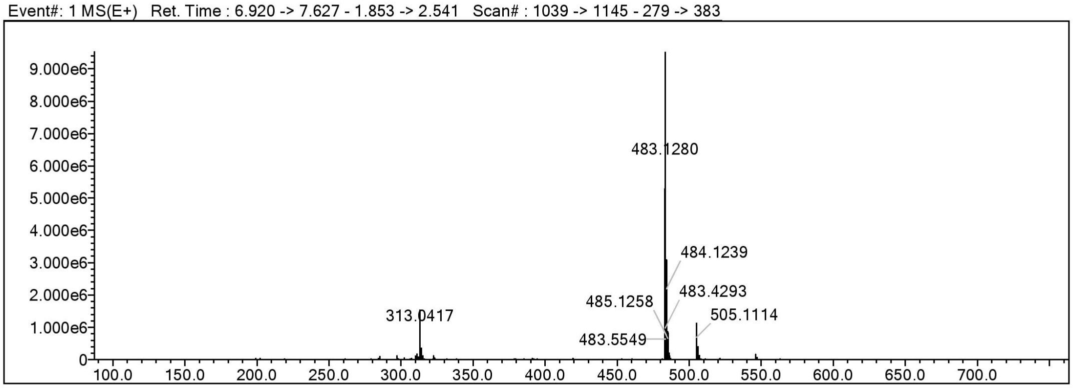

Measured region for 483.1280 m/z

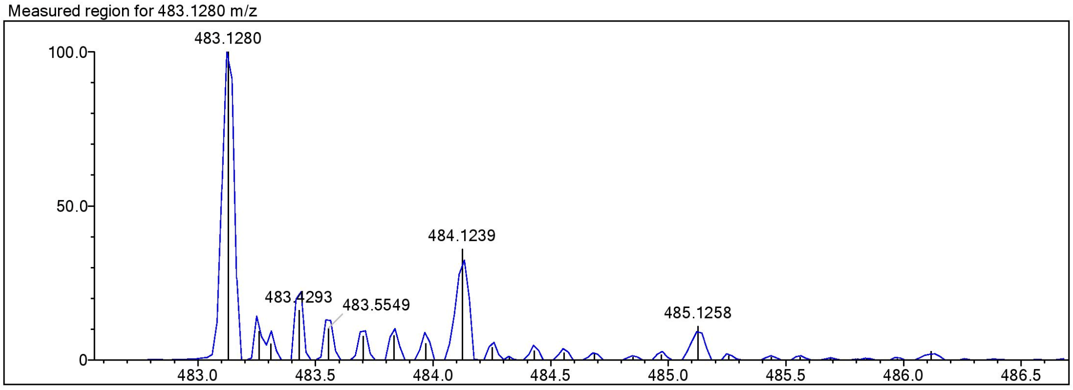

C27 H19 N4 O2 F S [M+H]+ : Predicted region for 483.1286 m/z

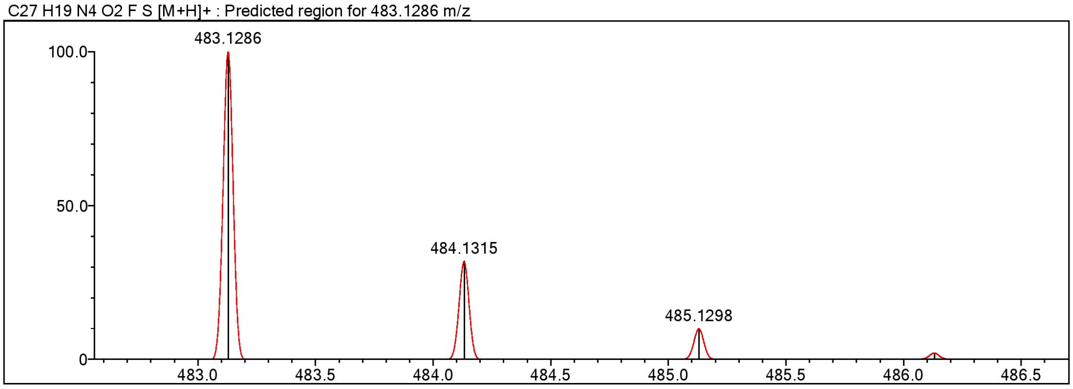

Rank	Score	Formula (M)	Ion	Meas. m/z	Pred. m/z	Df. (mDa)	Df. (ppm)	Iso	DBE
2	77.35	C27 H19 N4 O2 F S	[M+H]+	483.1280	483.1286	-0.6	-1.24	77.82	20.0

Figure S67. HRMS spectrum of compound **4w**

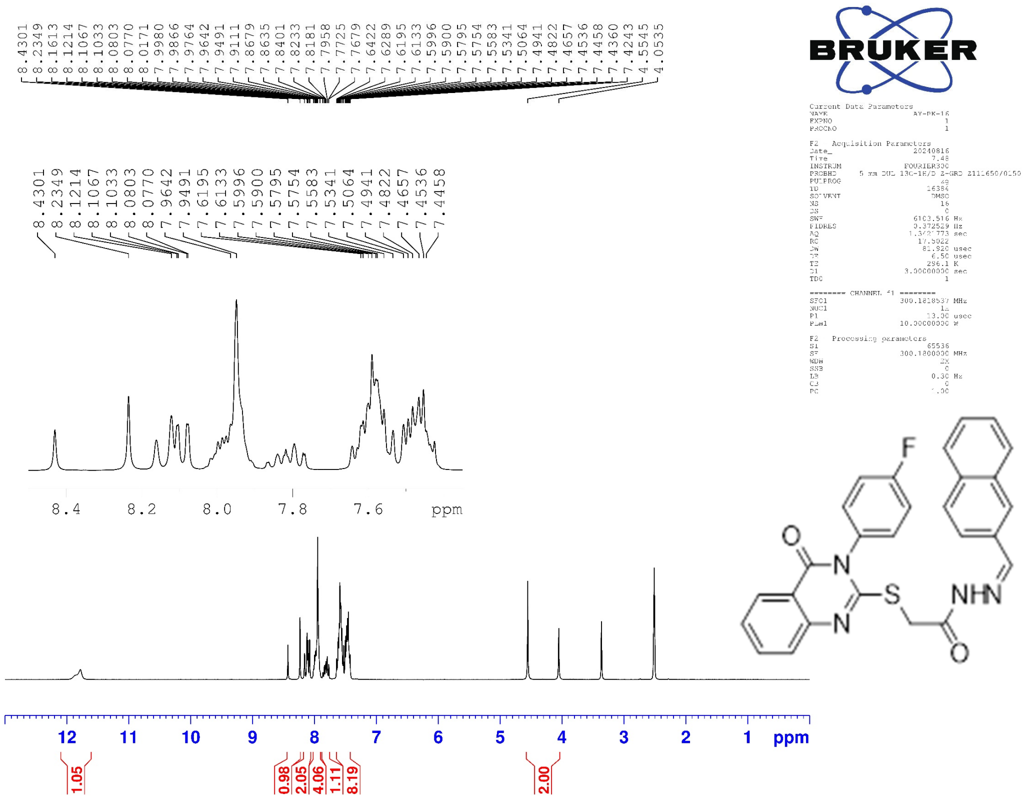

Figure S68. ^{1}H-NMR spectrum of compound **4w**

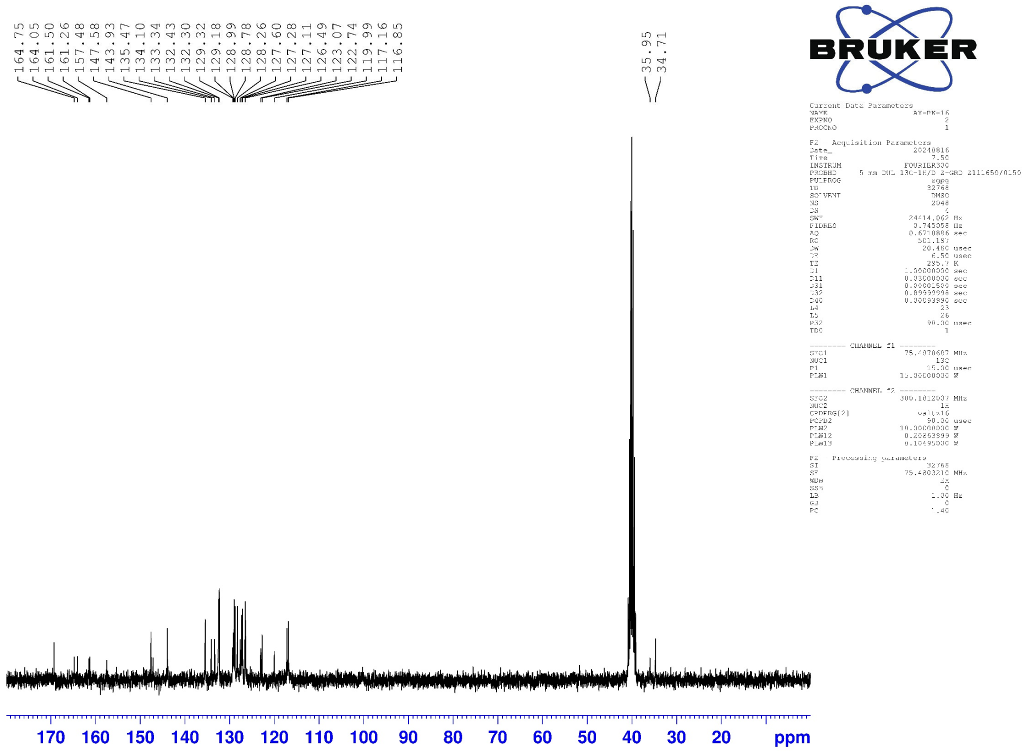

Figure S69. ^{13}C-NMR spectrum of compound **4w**

DOPNALAB

	Item	Value
	Acquired Date&Time	21.08.2026 11:28:52
	Acquired by	System Administrator
	Filename	C:\Users\dopnalab\Desktop\MASAÜSTÜ\FARMASÖTİK\aybuke\AY-PK-191.ispd
	Spectrum name	AY-PK-191
	Sample name	AY-PK-19
	Sample ID	
	Option	
	Comment	
	No. of Scans	45
	Resolution	4 [cm-1]
	Apodization	Happ-Genzel

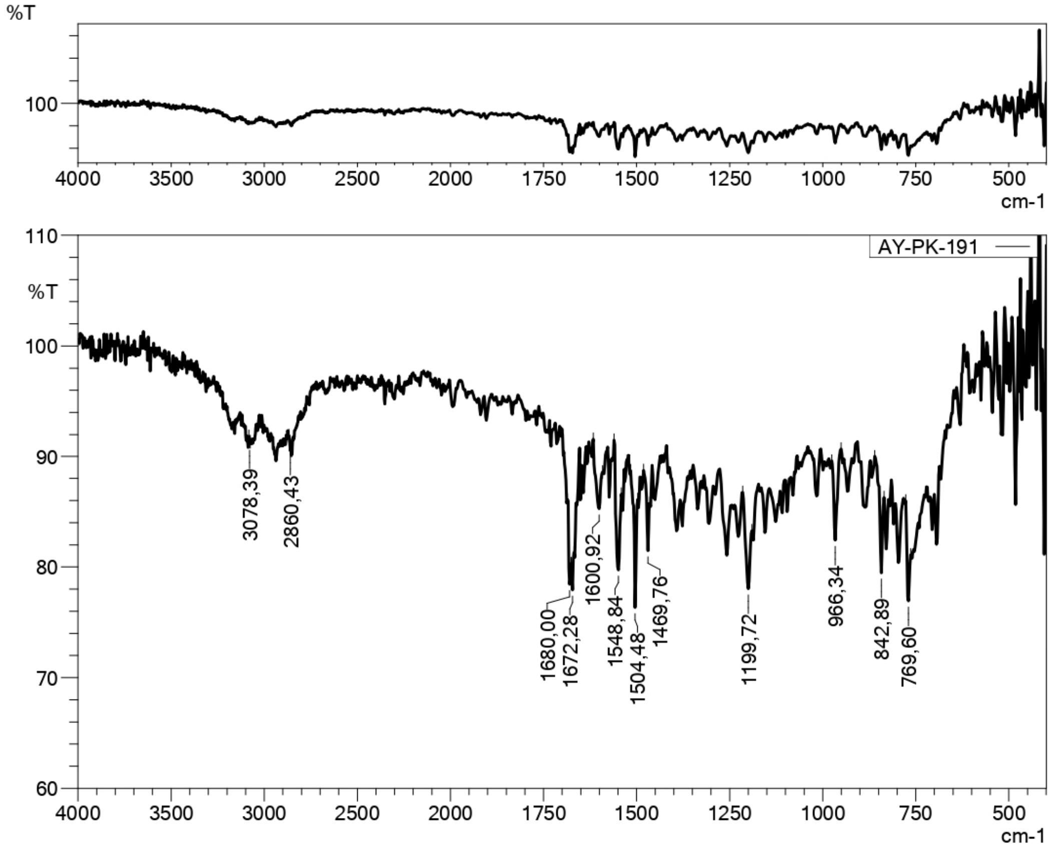

Figure S70. IR spectrum of compound **4x**

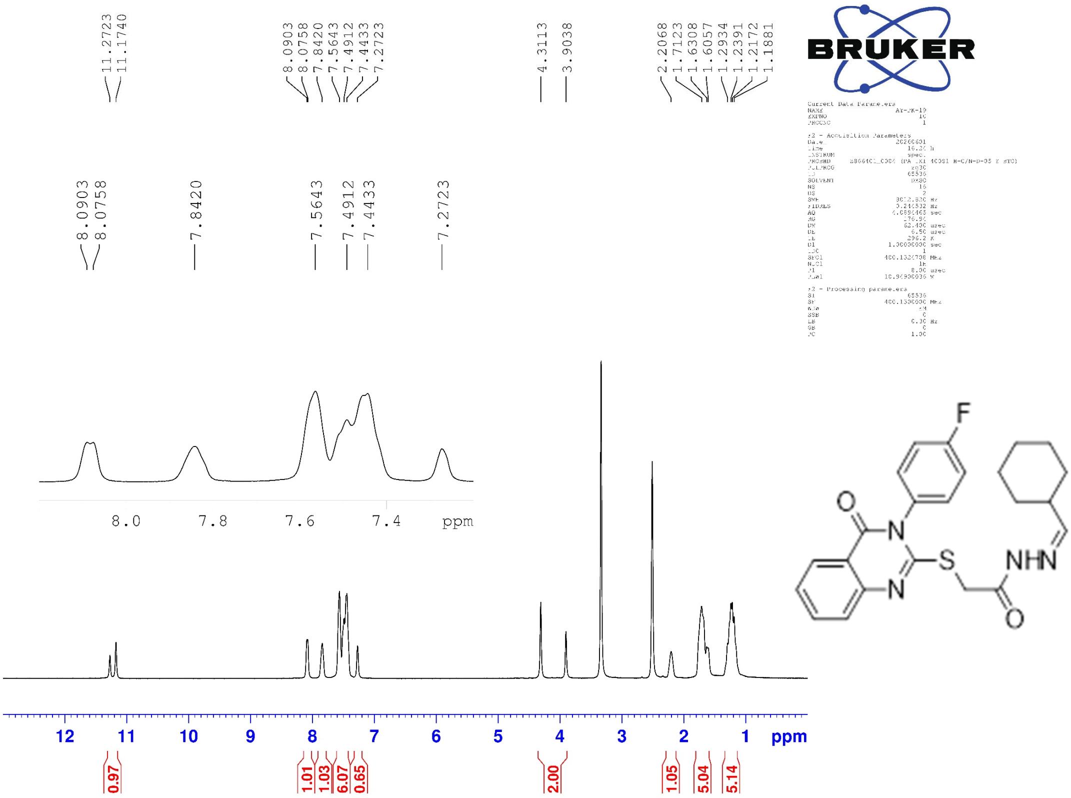

Figure S71. ^{1}H-NMR spectrum of compound **4x**

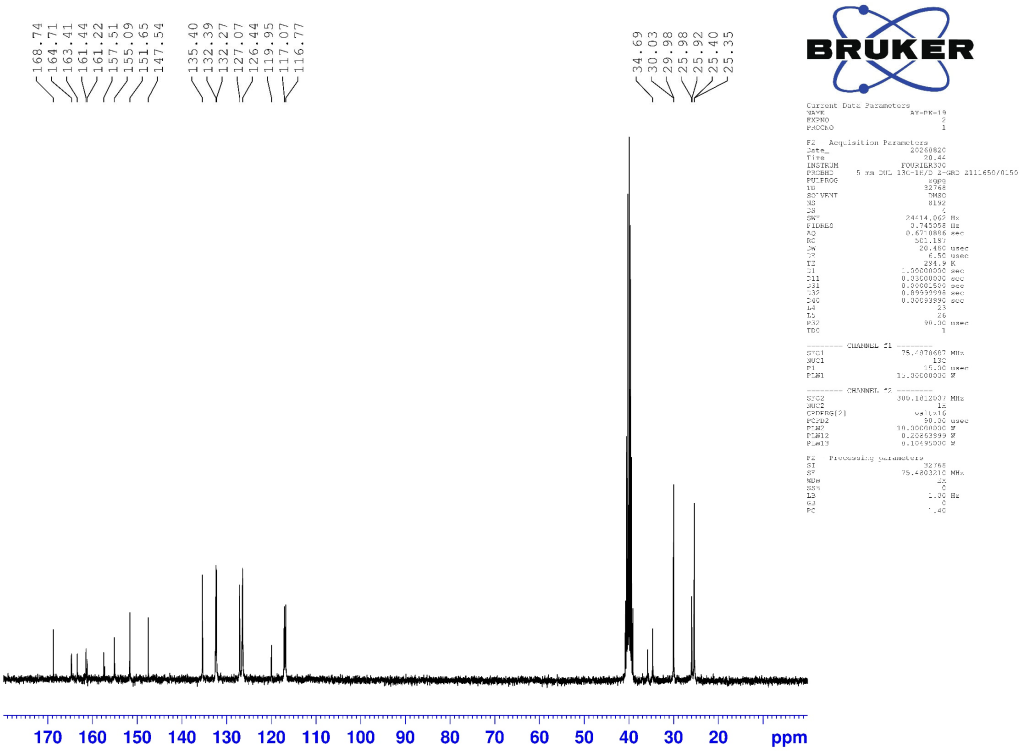

Figure S72. ^{13}C-NMR spectrum of compound **4x**
